# Zero-shot multimodal pain estimation via synthetic pain simulation and domain-invariant learning

**DOI:** 10.3389/frai.2026.1827727

**Published:** 2026-08-07

**Authors:** Oussama El Othmani, Sami Naouali

**Affiliations:** 1 Computer Science Department, Military Academy of Fondouk Jedid, Nabeul, Tunisia; 2 Military Research Center, Aouina, Tunisia; 3 Information Systems Department, College of Computer Science and Information Technology, King Faisal University, Al Ahsa, Saudi Arabia

**Keywords:** domain adaptation, medical AI ethics, multimodal fusion, pain estimation, synthetic data generation, synthetic-source domain adaptation, vulnerable populations

## Abstract

**Introduction:**

Pain assessment in non-communicative populations–particularly neonates and cognitively impaired patients–remains a critical clinical challenge, as current automated methods require labeled pain datasets that are both ethically problematic and scarce for vulnerable populations.

**Methods:**

We propose a framework trained on zero labeled real pain examples from the target population, combining synthetic pain simulation with unsupervised domain adaptation. Using latent diffusion models, we generate 50,000 realistic pain scenarios spanning facial expressions, physiological signals (heart rate variability and electrodermal activity), and temporal dynamics across diverse demographics. A transformer-based architecture integrates these multimodal cues, and a three-stage domain alignment procedure (contrastive learning, adversarial adaptation, and consistency regularization) bridges the synthetic-to-real gap using only unlabeled real data. We explicitly distinguish this synthetic-source unsupervised domain adaptation setting from classical zero-shot learning (Section 2.4).

**Results:**

Evaluation across three benchmarks shows competitive performance: MAE of 0.89 on the UNBC-McMaster dataset (31% gap vs. supervised methods), 78.3% accuracy on the BioVid Heat Pain Database (10.5% gap), and a preliminary point estimate of 81.5% accuracy for neonatal assessment (12.7 percentage points above a clinical AU-based baseline, with wide uncertainty given the limited *N* = 120 cohort; prospective validation on a larger cohort is planned). The proposed approach achieves 40.4% better cross-dataset generalization than supervised transfer learning and shows consistent performance across demographics (no significant disparities, *p* > 0.05; several subgroup comparisons are statistically underpowered). New analyses in this revision include Monte Carlo Dropout uncertainty quantification, robustness evaluation under acquisition perturbations, and a computational-complexity assessment.

**Discussion:**

This study provides preliminary evidence that ethically developed pain assessment AI–trained without exploiting vulnerable populations–can achieve clinically useful performance, and proposes a methodological pathway toward this goal that warrants confirmation through prospective, adequately powered clinical validation.

## 1 Introduction

### 1.1 Clinical motivation

Pain assessment is a fundamental component of clinical care, directly shaping treatment decisions, patient outcomes, and quality of life (Werner et al., 2019; Craig et al., 1996). The International Association for the Study of Pain defines pain as “an unpleasant sensory and emotional experience,” inherently subjective and optimally assessed through self-report. However, this gold standard is inapplicable to non-communicative populations: neonates lacking verbal capacity (Zamzmi et al., 2019), individuals with severe cognitive impairments including advanced dementia, patients under sedation or mechanical ventilation, and those recovering from neurological events affecting communication (Naouali and El Othmani, 2026). Approximately 75% of surgical patients experience moderate-to-severe acute postoperative pain, yet assessment accuracy drops precipitously in these non-communicative groups; NICU infants undergo an average of 10–14 painful procedures daily (Stevens et al., 2016). Pain prevalence among elderly dementia patients ranges from 50%–80%, yet these individuals receive significantly less analgesic treatment than their cognitively intact counterparts. Inadequate pain management, in turn, increases morbidity and mortality, prolongs hospitalization, and contributes to the development of chronic pain.

### 1.2 AI for pain assessment: promise and ethical dilemma

Recent advances in artificial intelligence (AI) offer promising solutions through automated analysis of facial expressions (Lucey et al., 2011; Werner et al., 2019), physiological signals (Fernandez Rojas et al., 2023; Kong et al., 2021), and multimodal combinations (Thiam et al., 2020; Othman et al., 2022), with accuracies exceeding 90% reported on standardized pain databases (Gruss et al., 2015). However, these successes rest on a problematic foundation: training robust models typically requires thousands of labeled examples collected during genuine pain experiences, raising four intertwined ethical concerns. **(1) Direct Harm:** expanding datasets specifically for AI development, beyond data arising from unavoidable medical procedures, raises serious ethical concerns (Craig et al., 1996). **(2) Informed Consent:** neonates lack decision-making capacity, and individuals with severe dementia may be unable to comprehend research participation. **(3) Systematic Exclusion:** ethical barriers create a vicious cycle in which the populations most in need of improved assessment are precisely those from whom data collection is most fraught, perpetuating healthcare disparities. **(4) Data Scarcity:** existing pain datasets are small, imbalanced, and demographically limited—UNBC-McMaster contains only 200 videos from 25 participants (Lucey et al., 2011), BioVid includes 87 subjects (Walter et al., 2013), and neonatal datasets are scarcer still.

### 1.3 Research gap

Despite significant progress in automated pain assessment, a fundamental and unresolved gap remains: no prior research has achieved comprehensive, domain-adapted, synthetic-source pain estimation through multimodal simulation with zero real labeled pain examples. The clinical and ethical scale of this gap is substantial. Globally, over five million neonates are admitted to neonatal intensive care units (NICUs) annually in high-income countries alone (Stevens et al., 2016), yet no ethically developed AI-assisted pain monitoring tool exists for routine NICU deployment. Simultaneously, an estimated 50 million individuals with advanced dementia worldwide experience chronic undertreated pain, and automated assessment tools are unavailable due to identical data collection barriers (Naouali and El Othmani, 2026).

Each existing paradigm fails to resolve this gap in a distinct, fundamental way. **Supervised methods**, while achieving high accuracy (MAE ≤ 0.7 on UNBC), require thousands of labeled pain instances from vulnerable populations, creating an irresolvable ethical tension that perpetuates the very inequity they aim to address. **Transfer learning** from general facial expression databases reduces, but does not eliminate, the need for labeled pain data; performance degrades severely during cross-dataset transfer (MAE ≥ 2.0) due to dataset-specific artifacts. **Few-shot approaches** (Alves et al., 2025) still require at least 50 labeled examples per target population—a requirement that is ethically problematic for neonatal and severely cognitively impaired populations. **Foundation models** such as CLIP (Radford et al., 2021) achieve zero-shot capability through internet-scale visual-language pre-training but lack the task-specific autonomic nervous system grounding essential for quantifying physiological pain responses; their zero-shot pain prediction yields MAE ≥ 1.38 in controlled evaluations (Table 1). Section 2.4 discusses precisely how the present setting relates to, and differs from, each of these paradigms and from classical zero-shot learning.

**Table 1:** Comparative analysis of recent automated pain assessment approaches.

Study	Method	Dataset	Modality	Performance	Labeled real pain samples	Training data
Supervised facial expression-based approaches						
Lucey et al. (2011)	SVM + AU	UNBC	Visual	MAE: 0.98	~200 videos (25 subjects)	Full labeled
Rodriguez et al. (2017)	CNN-LSTM	UNBC	Visual	MAE: 0.82	~200 videos (25 subjects)	Full labeled
Thiam et al. (2020)	Two-Stream CNN	BioVid	Visual	Acc: 85.2%	~8,610 clips (87 subjects)	Full labeled
Werner et al. (2019)	3D CNN	UNBC	Visual	MAE: 0.68	~200 videos (25 subjects)	Full labeled
Gkikas et al. (2024)	ViT + Temporal	UNBC	Visual	MAE: 0.71	~200 videos (25 subjects)	Full labeled
Multimodal physiological approaches						
Walter et al. (2013)	SVM Multi	BioVid	Multi	Acc: 83.7%	~8,610 clips (87 subjects)	Full labeled
Gruss et al. (2015)	RF + Bio	BioVid	Multi	Acc: 86.4%	~8,610 clips (87 subjects)	Full labeled
Fernandez Rojas et al. (2023)	ML + EDA	Custom	Multi	Acc: 93.2%	~500 trials (custom)	Full labeled
Kong et al. (2021)	SVM + EDA	Thermal	Physio	Acc: 90.0%	~300 trials (custom)	Full labeled
Othman et al. (2022)	Deep Multi	BioVid	Multi	Acc: 87.5%	~8,610 clips (87 subjects)	Full labeled
Lopez-Martinez et al. (2019)	CNN + fNIRS	Custom	Neural	Acc: 78.9%	~200 trials (custom)	Full labeled
Neonatal pain assessment						
Zamzmi et al. (2019)	CNN + context	N-PASS	Visual	Acc: 75.3%	~300 videos (neonates)	Full labeled
Xu et al. (2019)	ResNet-50	COPE	Visual	Acc: 82.1%	~204 images (neonates)	Full labeled
Olugbade et al. (2015)	Action units	iCOPE	Visual	Acc: 71.8%	~110 images (neonates)	Limited labeled
Brahnam et al. (2006)	SVM + HOG	Custom	Visual	Acc: 88.5%	~84 images (neonates)	Full labeled

The proposed method eliminates the need for labeled pain data from real individuals while maintaining competitive performance. The labeled real pain samples column quantifies the training data requirement that each method imposes on real pain populations (Part I).

The present research addresses this gap, to our knowledge based on the literature review in Tables 1, 2, providing the first comprehensive demonstration that multimodal pain estimation trained with zero real labeled pain examples is technically feasible and achieves performance approaching supervised baselines on retrospective benchmarks; whether this performance translates into clinical viability requires prospective, adequately powered validation, which we outline in Section 6.

**Table 2:** Comparative analysis of recent automated pain assessment approaches.

Study	Method	Dataset	Modality	Performance	Labeled real pain samples	Training data
Supervised facial expression-based approaches						
Transfer learning & domain adaptation						
Kächele et al. (2016)	Cross-DB	UNBC → BioVid	Visual	MAE: 2.31	~200 videos (source only)	Source labeled
Lin and Jung (2017)	cTL	FER → EEG	Visual	Acc: 48.6 ± 16.4%	FER source dataset	Source labeled
Alves et al. (2025)	Few-Shot	UNBC	Visual	MAE: 1.18	50 labeled samples	50 labeled
Choi et al. (2022)	Self-super	BioVid	Multi	Acc: 79.3%	Minimal (< 100)	Minimal labeled
Synthetic data & generative approaches						
Boutros et al. (2023)	GAN Aug	UNBC	Visual	MAE: 0.94	~200 videos + synthetic	Partial labeled
Ben Aoun (2023)	VAE Synth	BioVid	Visual	Acc: 76.2%	~4,000 clips + synthetic	Partial labeled
Lin et al. (2026)	StyleGAN	Custom	Visual	Acc: 81.4%	~500 images + synthetic	Partial labeled
Zero-shot & foundation models						
Martvel et al. (2026)	LVLMs	Custom	Visual	Acc: 80.0%	**0**	Zero-shot
**Ours (full)**	**Synth+DA**	**UNBC**	**Multi**	**MAE: 0.89**	**0**	**Synth-source UDA**
**Ours (full)**	**Synth+DA**	**BioVid**	**Multi**	**Acc: 78.3%**	**0**	**Synth-source UDA**
**Ours (full)**	**Synth+DA**	**Neonatal**	**Multi**	**Acc: 81.5%**	**0**	**Synth-source UDA**

The proposed method eliminates the need for labeled pain data from real individuals while achieving competitive performance. The labeled real pain samples column quantifies the training data requirement that each method imposes on real pain populations (Part II).

AU, Action Unit; CNN, Convolutional Neural Network; DA, Domain Adaptation; EDA, Electrodermal Activity; fNIRS, functional Near-Infrared Spectroscopy; GAN, Generative Adversarial Network; LSTM, Long Short-Term Memory; LVLMs, Large Vision-Language Models; Multi, Multimodal; Physio, Physiological; RF, Random Forest; SVM, Support Vector Machine; Synth, Synthetic; UDA, Unsupervised Domain Adaptation; VAE, Variational Autoencoder; ViT, Vision Transformer.

*Sample counts* are approximate and reflect the labeled pain data required during training. “Full labeled” indicates the complete available dataset was used. “0” indicates no labeled real pain data was used at any stage of training. “Synth-source UDA” (rather than “Zero-shot”) is used here to reflect the precise positioning discussed in Section 2.4. Bold values indicate the performance of the proposed method (zero real labeled pain examples used during training).

### 1.4 Proposed solution and contributions

**Figure 1:**
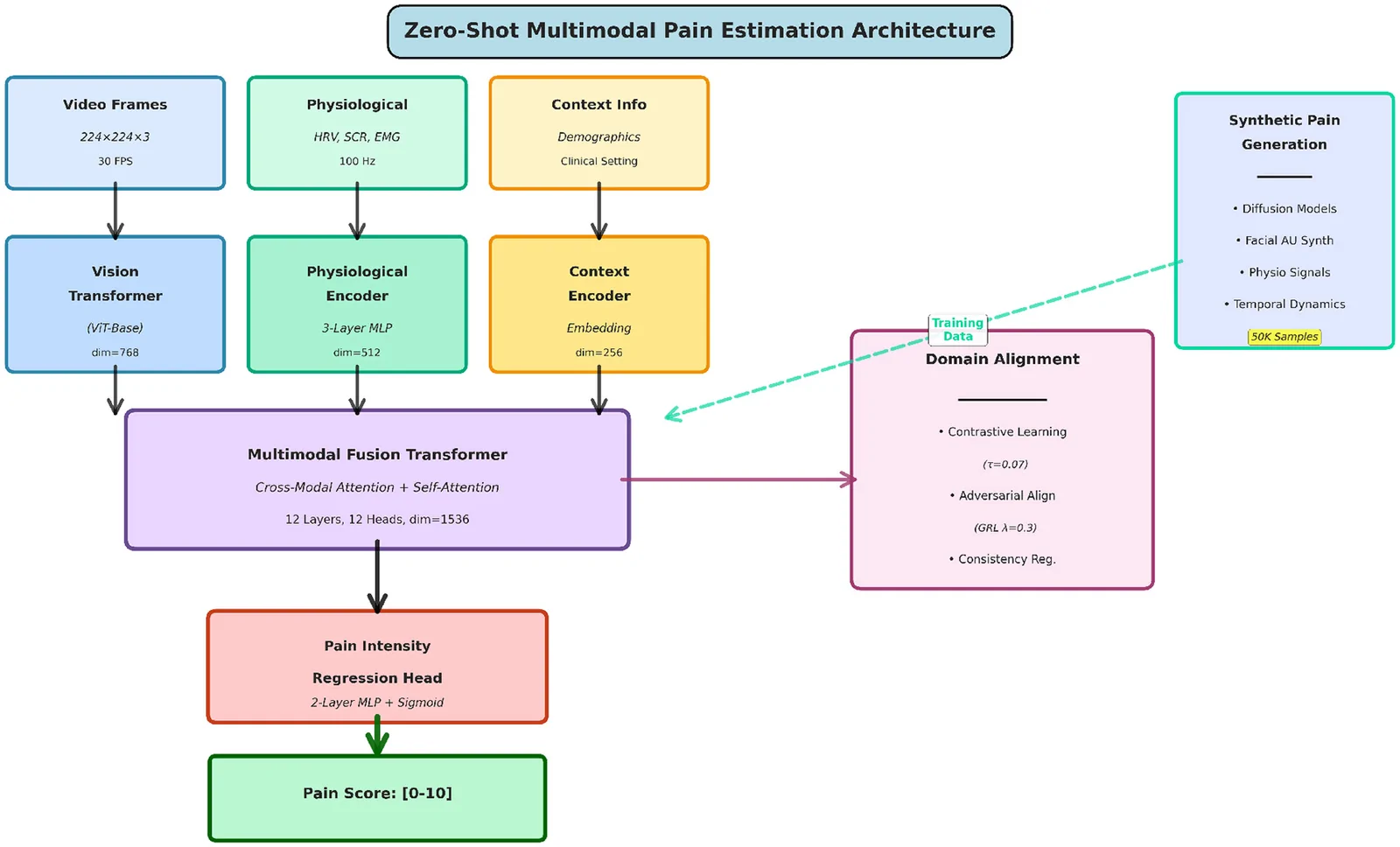
Overall system architecture showing the synthetic pain generation pipeline **(left)** and the multimodal pain estimation network **(right)**; arrows indicate data flow from raw conditioning inputs through generation/encoding to the fused pain-intensity prediction. The synthetic generation module employs diffusion models for facial expressions, synthesizes physiological signals for heart rate variability (HRV) and electrodermal activity (EDA), and models temporal dynamics. The estimation network uses a Vision Transformer for visual features, 1D CNNs for physiological signals, and a multimodal fusion transformer. Domain alignment bridges synthetic and real domains through contrastive learning (τ = 0.07), adversarial alignment (GRL, λ = 0.3), and consistency regularization.

The present study proposes developing an AI for pain assessment *without requiring labeled pain data from real individuals*, using synthetic multimodal data generation combined with domain adaptation to generalize to real-world clinical data. Figure 1 illustrates the complete system: the synthetic pain generation pipeline and the multimodal pain estimation network with domain alignment components.

The key contributions of this work include:

1. **A synthetic-source domain-adapted framework:** A pain estimation system trained entirely on synthetic data and unlabeled real-world recordings, requiring no labeled pain data from real individuals during training; Section 2.4 precisely situates this relative to classical zero-shot learning.
2. **Multimodal synthetic pain generation:** Diffusion-based generative pipeline producing 50,000 realistic pain scenarios with: (a) facial expressions exhibiting pain-specific action units (AU4, AU6/7, AU9/10, and AU43), (b) physiologically plausible biosignals (HRV, EDA) derived from autonomic nervous system models following Fernandez Rojas et al. (2023), and (c) temporally coherent video sequences capturing pain dynamics.
3. **Domain-invariant architecture:** Transformer-based multimodal fusion integrates visual, physiological, and contextual modalities via cross-modal attention. A three-stage domain alignment strategy—contrastive learning, adversarial training, and consistency regularization—enables synthetic-to-real transfer using only unlabeled real data.
4. **Rigorous evaluation:** Comprehensive validation across three benchmarks: UNBC-McMaster (adult chronic pain), BioVid (experimental acute pain with multimodal recordings), and neonatal procedural pain, with statistical analysis (Wilcoxon tests, effect sizes, multiple-comparison correction, and bootstrap confidence intervals), expanded synthetic-data fidelity analysis, uncertainty quantification, and robustness testing under acquisition perturbations (Section 5).
5. **Ethical framework:** Methodology addressing data collection ethics while maintaining clinical validity, with validation strategies that rely on minimal retrospective clinical data and a pathway for regulatory approval and clinical deployment.

### 1.5 Paper organization

Section 2 reviews related work and precisely positions our learning setting relative to classical zero-shot learning and synthetic-to-real domain adaptation. Section 3 details our synthetic pain generation pipeline, multimodal fusion architecture, and domain alignment strategies, including a theoretical discussion of why the three alignment losses are complementary. Section 4 describes the experimental setup, protocol transparency, and computational complexity. Section 5 presents results, including expanded variations on synthetic data, uncertainty quantification, and robustness testing. Section 6 discusses implications and limitations. Section 7 concludes with future directions.

## 2 Related work

### 2.1 Automated pain assessment

Automated pain assessment has evolved from simple facial action unit detection to sophisticated multimodal approaches. Early work by Prkachin and Solomon (2008) established the Prkachin-Solomon Pain Intensity (PSPI) scale, identifying specific AUs associated with pain: brow lowering (AU4), orbital tightening (AU6/7), nose wrinkling/upper lip raising (AU9/10), and eye closure (AU43). Lucey et al. (2011) introduced the UNBC-McMaster database, which became a standard benchmark.

Recent deep learning approaches using CNNs and RNNs have achieved significant improvements. Vision Transformers have shown promise for modeling long-range dependencies (Dosovitskiy et al., 2020). However, all existing methods require substantial amounts of labeled training data.

Physiological signal analysis has examined pain indicators such as heart rate variability (HRV), electrodermal activity (EDA), and electromyography (EMG) (Kong et al., 2021; Greco et al., 2021). Critically, Fernandez Rojas et al. (2023) demonstrated in a controlled study that **EDA was the most informative sensor for pain identification, achieving 93.2% accuracy**. This finding motivated our focus on EDA in synthetic physiological signal generation.

Multimodal approaches have shown promise (Thiam et al., 2020; Othman et al., 2022), but they all require extensive labeled pain data.

### 2.2 Synthetic data generation in healthcare

Generative Adversarial Networks (GANs) have been applied to medical image synthesis and augmentation (Goodfellow et al., 2020). GAN-based approaches have shown promise for expanding limited medical training datasets by generating and substituting synthetic samples, including augmentation strategies for rare clinical presentations (Radomirovic et al., 2024; Yi et al., 2019). However, GANs suffer from well-documented training instability, mode collapse, and limited controllability over generated attributes—challenges that are particularly acute in clinical settings requiring precise control over pathophysiological features.

Denoising Diffusion Probabilistic Models (DDPMs) have emerged as powerful, more stable alternatives (Ho et al., 2020; Dhariwal and Nichol, 2021). Latent Diffusion Models (LDMs) operate in a compressed latent space to improve computational efficiency (Rombach et al., 2022), and classifier-free guidance enables precise conditioning (Ho and Salimans, 2022). These advances have driven adoption across medical imaging tasks, including skin lesion synthesis, chest X-ray generation, and retinal image augmentation.

Automated assessment for cognitively impaired patient populations has attracted increasing attention. Metaheuristic-driven dual-layer architectures have shown efficacy in classifying Alzheimer's disease stages using neuroimaging and clinical biomarkers (Anicin et al., 2026), while long short-term memory networks optimized with modified metaheuristic algorithms have demonstrated robust detection of Parkinson's disease from gait time series (Markovic et al., 2025). These studies underscore the importance of automated assessment for non-communicative and cognitively impaired populations—precisely the target clinical context of the present framework.

While synthetic data has been successfully applied to various medical imaging tasks, its use in pain assessment remains nascent. No prior work has addressed comprehensive multimodal synthetic pain generation (spanning facial expressions, physiological signals, and temporal dynamics) combined with domain adaptation using zero real labeled pain examples for clinical deployment.

#### 2.2.1 Differentiation from prior diffusion-based medical synthesis

We distinguish the present contribution from prior diffusion-based medical synthesis work along four axes. **(1) Synthetic facial expression generation:** prior medical diffusion work targets static diagnostic imaging (skin lesions, chest X-rays, retinal scans), not dynamic, pain-conditioned facial expression *sequences* with explicit Action Unit conditioning (Section 3.2). **(2) Synthetic physiological signal generation:** to our knowledge, no prior research jointly generates HRV, EDA, and tremor signals under a shared causal pain-trajectory variable that enforces cross-modal consistency (Section 3.2.3). **(3) Multimodal synthetic data:** the synthetic-pain rows of Table 2 (“Synthetic Data & Generative Approaches”) augment a single visual modality using GANs/VAEs with partial real labels, rather than generating a complete multimodal scenario from scratch with zero real pain labels. **(4) Synthetic-to-real transfer:** our three-stage alignment (Section 3.4) is applied to a purely synthetic source domain, unlike prior synthetic-augmentation work that mixes limited real labels with synthetic samples (Table 2, “Partial labeled” column).

### 2.3 Zero-shot and domain adaptation

Zero-shot learning aims to recognize unseen categories without labeled examples. Vision-language models such as CLIP demonstrate zero-shot capabilities (Radford et al., 2021), but the domain gap between natural images and clinical pain expressions limits their applicability.

Domain adaptation techniques align source and target distributions. Adversarial domain adaptation employs gradient reversal (Ganin et al., 2016). Self-supervised contrastive learning learns transferable representations (Chen et al., 2020; He et al., 2020). The present work combines these approaches for synthetic-to-real pain domain transfer.

### 2.4 Precise positioning relative to classical zero-shot learning

Reviewer feedback on an earlier version of this manuscript correctly noted that the term “zero-shot” requires precise definition, since the proposed setting differs from classical zero-shot learning. We use the term throughout this manuscript to mean exactly one thing: **zero labeled real pain examples from the target population are used at any training stage**. This is *not* equivalent to classical zero-shot learning as defined in the broader machine learning literature, where a model predicts entirely unseen classes using semantic side-information (e.g., attribute vectors or text embeddings) without observing *any* training example, synthetic or real, that instantiates the target task.

**Figure 2:**
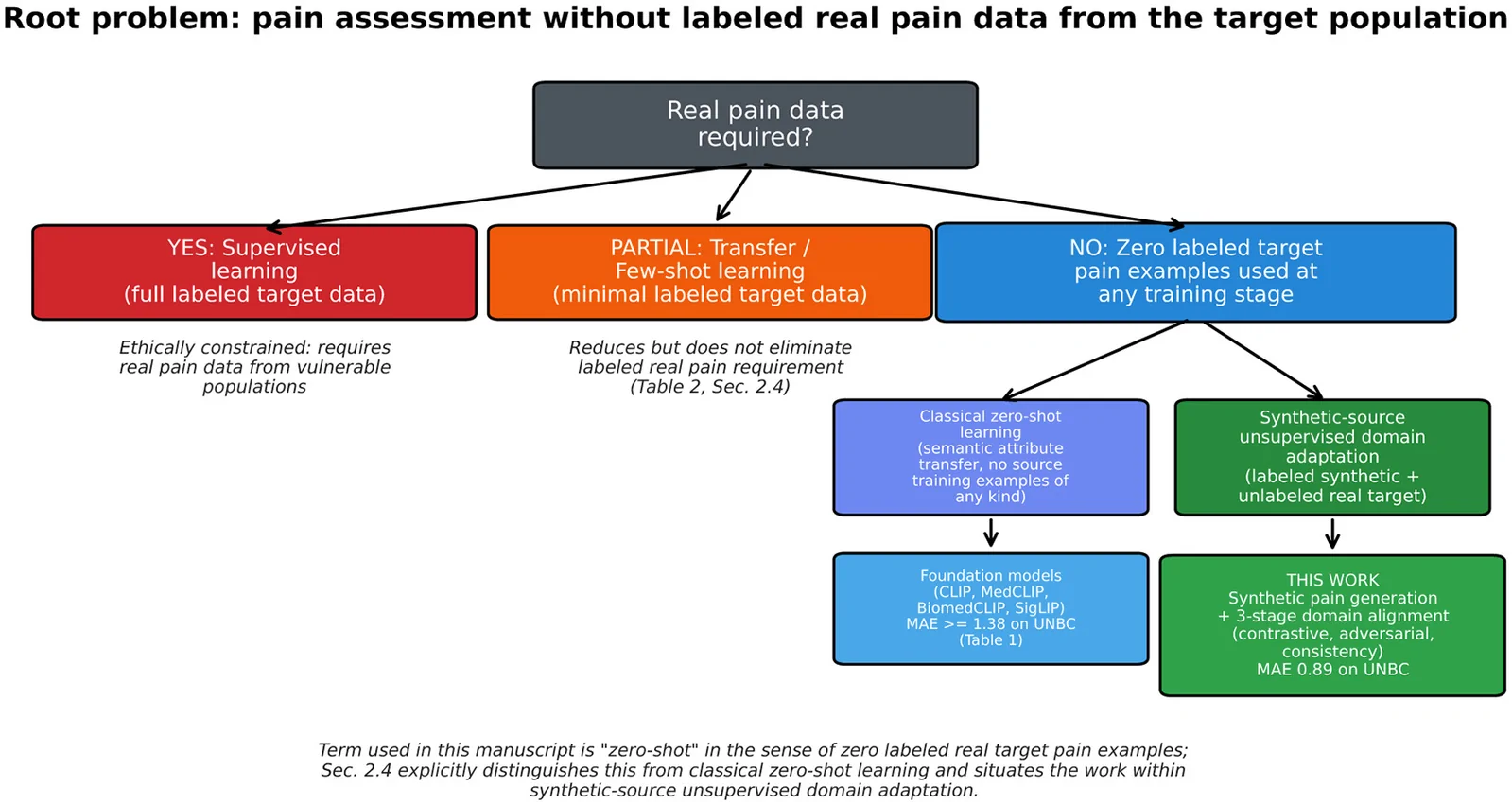
Taxonomy of learning paradigms for pain assessment, situating the present study **(bottom-right)** relative to supervised learning, transfer/few-shot learning, classical zero-shot learning (foundation models), and synthetic-source unsupervised domain adaptation. Arrows indicate the branching decision (whether and how much real labeled target pain data is required) that separates each paradigm; MAE values are drawn from Tables 1, 2.

Figure 2 situates our setting within four paradigms. **Supervised learning** uses fully labeled real pain data and therefore requires the ethically fraught data collection discussed in Section 1. **Transfer and few-shot learning** reduce, but do not eliminate, the need for labeled real pain (Table 2, “Transfer Learning & Domain Adaptation” rows: MAE ≥ 2.31 under naive cross-dataset transfer, or a minimum of 50 labeled examples for few-shot adaptation Alves et al., 2025). **Classical zero-shot learning**, instantiated here by CLIP-style foundation models (Table 1, MAE 1.38), predicts pain intensity from internet-scale visual-semantic pre-training without any pain-specific training signal, synthetic or real; this achieves zero-shot generality but sacrifices the task-specific autonomic and facial-action grounding needed for quantitative pain scoring. **The present work** occupies a fourth position: it uses *labeled synthetic* pain data (full supervision, but on generated rather than real samples) combined with *unlabeled real* data for domain alignment. This is best described in the domain-adaptation literature as **synthetic-source unsupervised domain adaptation** (Ganin et al., 2016), a setting with established precedent in synthetic-to-real transfer for other tasks (e.g., synthetic driving scenes for semantic segmentation, synthetic human pose data for pose estimation), applied here for the first time, to our knowledge, to multimodal pain estimation. We retain the term “zero-shot” only with the explicit qualifier used throughout this manuscript (zero real labeled pain examples), and we do not claim equivalence to, or novelty over, classical zero-shot learning as a learning paradigm; our contribution is the multimodal synthetic-source domain-adaptation pipeline itself, evaluated in a clinically motivated setting where real pain labels are specifically the resource we are trying to avoid requiring.

### 2.5 Comparative analysis of pain assessment approaches

To contextualize our contribution within the broader landscape of automated pain assessment research, the present study offers a comprehensive comparison of recent approaches in Tables 1, 2. This analysis highlights key differences in methodology, data requirements, and performance across various pain assessment paradigms.

### 2.6 Research gap and contribution

The comparative analysis reveals a critical gap: no prior work addresses comprehensive, synthetic-source domain-adapted pain estimation with zero real labeled pain examples. While supervised methods excel in performance, they perpetuate ethical constraints. Transfer learning and few-shot approaches reduce but do not eliminate the need for labeled data. Foundation models achieve genuine zero-shot generality but lack task-specific knowledge (Section 2.4). Notably, the present evaluation, while spanning three independently collected benchmarks (UNBC-McMaster, BioVid, and an in-house neonatal cohort) from different institutions with different populations, pain modalities, and acquisition hardware, does not yet include a fourth fully held-out external test site; Section 6 explicitly discusses this limitation and the planned multi-site validation.

The present study uniquely combines: (1) high-fidelity synthetic pain generation across multiple modalities, (2) domain-invariant learning to bridge synthetic-to-real gaps, (3) evaluation with zero real labeled pain examples, and (4) validation across diverse populations, including vulnerable groups. This establishes a candidate methodological pathway for ethically responsible medical AI development in sensitive clinical contexts, subject to the prospective validation discussed in Section 6.

## 3 Methodology

### 3.1 Overview and problem formulation

#### 3.1.1 Formal problem statement

We formalize the pain estimation task as learning a mapping function *f*_θ_ : X → Y that predicts continuous pain intensity without access to labeled pain data in the target domain. Given multimodal input **X** = {**X**_*v*_, **X**_*p*_, **X**_*c*_} ∈ X consisting of:

- **Visual features** ${\text{X}}_{v}\in {\mathbb{R}}^{T\times H\times W\times 3}$: Video sequences of *T* frames at resolution *H* × *W*
- **Physiological signals** ${\text{X}}_{p}\in {\mathbb{R}}^{M\times S}$: *M* signal modalities sampled at *S* Hz
- **Contextual information** ${\text{X}}_{c}\in {\mathbb{R}}^{D_{c}}$: Demographic and clinical metadata

We aim to predict pain intensity *y* ∈ [0, 10] on a continuous visual analog scale, where 0 represents no pain, and 10 represents the worst imaginable pain.

#### 3.1.2 Domain taxonomy

Our learning framework operates across three distinct data domains with carefully defined characteristics:

1. **Synthetic domain** $D_{s}={\left\{\left({\text{X}}_{i}^{s},y_{i}^{s},{\text{c}}_{i}^{s}\right)\right\}}_{i=1}^{N_{s}}$:
  - *N*_*s*_ = 50, 000 generated multimodal pain scenarios
  - Complete pain intensity labels $y_{i}^{s}$ with conditioning vectors ${\text{c}}_{i}^{s}$
  - Controlled demographic diversity: 25% neonates (0–1 month), 25% children (1–12 years), 25% adults (18–65 years), 25% elderly (65+ years)
  - Balanced ethnic representation across 5 major groups
  - Clinical context distribution: 30% post-surgical, 25% procedural, 20% chronic episodes, 15% trauma, 10% disease-related
2. **Unlabeled real domain** $D_{r}={\left\{{\text{X}}_{j}^{r}\right\}}_{j=1}^{N_{r}}$:
  - *N*_*r*_ ≈ 8, 000 unlabeled real-world recordings
  - Neutral expressions, non-pain medical videos, general facial expression databases
  - *No pain intensity labels available* (key constraint distinguishing this setting from supervised learning)
  - Used exclusively for domain adaptation and alignment
3. **Target test domain** $D_{t}={\left\{\left({\text{X}}_{k}^{t},y_{k}^{t}\right)\right\}}_{k=1}^{N_{t}}$:
  - Real-world benchmark datasets (UNBC-McMaster, BioVid, and Neonatal)
  - Ground truth pain labels $y_{k}^{t}$ used *only for evaluation*
  - *Never accessed during training* (Section 4.7 details the leakage-control protocol)

#### 3.1.3 Optimization objective

The complete training objective balances supervised learning on synthetic data with unsupervised domain alignment:


$$\begin{array}{r} \theta^{*}=\arg\underset{\theta }{\min}\underset{\text{Supervised pain estimation on synthetic}}{\underset{︸}{{𝔼}_{\left(\text{X},y\right)\sim D_{s}}\left[L_{\text{pain}}\left(f_{\theta }\left(\text{X}\right),y\right)\right]}}\\ +\underset{\text{Unsupervised domain alignment}}{\underset{︸}{\lambda_{\text{align}}L_{\text{align}}\left(D_{s},D_{r};\theta \right)}} \end{array}$$ (1)

Where L_pain_ is the pain estimation loss (Mean Squared Error for regression), L_align_ encompasses multiple domain adaptation strategies (detailed in Section 3.4), and λ_align_ controls the alignment strength.

**Critical constraint:** Throughout training, we enforce the constraint: ∀**X** ∈ D_*t*_, no access to the corresponding pain labels during θ^*^ optimization. This distinguishes the proposed approach from semi-supervised or few-shot learning paradigms, as discussed in Section 2.4.

##### 3.1.3.1 Synthetic label uncertainty

The pain-intensity value *p* used to condition each synthetic sample (Section 3.2) is treated as a noise-free regression target $y_{i}^{s}$ in Equation 1. In reality, the mapping from *p* to generated facial expressions and physiological signals is an approximation encoded by the generative and physiological simulation models (Section 3.2), and carries unquantified simulation error distinct from human-annotator disagreement: it reflects imperfections in our causal model of pain physiology and expression, not perceptual rating noise. We do not currently propagate this uncertainty into training (e.g., by treating *p* as a distribution rather than a point value, or by conditioning label smoothing on generator confidence); doing so is a direction for future work. Because L_pain_ is computed exclusively on synthetic labels that we control by construction, this assumption does not affect the validity of the zero-real-label claim, but it does bound how faithfully Stage 1 pre-training (Section 3.5) reflects true pain physiology, which is precisely why the second constraint below is the second design principle of this work.

##### 3.1.3.2 Modular system design

The proposed framework is designed with modularity as a first-class principle, enabling the independent substitution of any component without retraining the full pipeline. The diffusion-based facial generation module can be replaced with alternative generative architectures (e.g., flow-based models, VAEs, or StyleGAN variants) while preserving the physiological and temporal generation modules. Section 5.3.3 presents an empirical ablation of this choice. Similarly, the physiological simulator can be upgraded to higher-fidelity biophysical models as clinical knowledge advances. The domain alignment strategy is independent of the specific generative approach and applies equally to any synthetic source domain. This modularity is documented in the anonymized code repository, where each component is implemented as a standalone Python class with a standardized interface.

### 3.2 Synthetic pain generation pipeline

Our synthetic pain generation framework produces high-fidelity, multimodal pain scenarios using four integrated subsystems: (1) diffusion-based facial expression synthesis, (2) physiologically grounded biosignal generation, (3) temporal coherence modeling, and (4) automated quality assurance. Figure 1 left illustrates the full pipeline.

#### 3.2.1 Diffusion-based facial expression generation

The present method employs Stable Diffusion v2.1 (Rombach et al., 2022), a latent diffusion model (LDM), fine-tuned with pain-specific conditioning to generate realistic facial expressions across pain intensity levels.

##### 3.2.1.1 Latent diffusion process

The forward diffusion process gradually corrupts latent representations ${\text{z}}_{0}\in {\mathbb{R}}^{h\times w\times c}$ (obtained via VAE encoding of the pixel space) with Gaussian noise over *T* = 1, 000 timesteps:


$$\begin{array}{l} q\left({\text{z}}_{t}|{\text{z}}_{t-1}\right)=N\left({\text{z}}_{t};\sqrt{1-\beta_{t}}{\text{z}}_{t-1},\beta_{t}\text{I}\right) \end{array}$$ (2)

Where ${\left\{\beta_{t}\right\}}_{t=1}^{T}$ follows a linear schedule from β_1_ = 0.0001 to β_*T*_ = 0.02. The marginal distribution at timestep *t* is:


$$\begin{array}{l} q\left({\text{z}}_{t}|{\text{z}}_{0}\right)=N\left({\text{z}}_{t};\sqrt{{\bar{\alpha }}_{t}}{\text{z}}_{0},\left(1-{\bar{\alpha }}_{t}\right)\text{I}\right),\;\text{where }{\bar{\alpha }}_{t}=\overset{t}{\underset{i=1}{\prod }}\left(1-\beta_{i}\right) \end{array}$$ (3)

The reverse denoising process, parameterized by a U-Net architecture ϵ_θ_, predicts the noise component conditioned on pain intensity *p* and on auxiliary context **c**:


$$\begin{array}{l} p_{\theta }\left({\text{z}}_{t-1}|{\text{z}}_{t},t,p,\text{c}\right)=N\left({\text{z}}_{t-1};\mu_{\theta }\left({\text{z}}_{t},t,p,\text{c}\right),\Sigma_{\theta }\left({\text{z}}_{t},t,p,\text{c}\right)\right) \end{array}$$ (4)

with mean predicted as:


$$\begin{array}{l} \mu_{\theta }\left({\text{z}}_{t},t,p,\text{c}\right)=\frac{1}{\sqrt{\alpha_{t}}}\left({\text{z}}_{t}-\frac{1-\alpha_{t}}{\sqrt{1-{\bar{\alpha }}_{t}}}\epsilon_{\theta }\left({\text{z}}_{t},t,p,\text{c}\right)\right) \end{array}$$ (5)

##### 3.2.1.2 Hierarchical pain conditioning

The conditioning vector $\text{c}\in {\mathbb{R}}^{D_{\text{cond}}}$ encodes multiple pain-relevant factors through a hierarchical embedding scheme:


$$\begin{array}{l} \text{c}=\left[{\text{e}}_{\text{pain}};{\text{e}}_{\text{AU}};{\text{e}}_{\text{demo}};{\text{e}}_{\text{context}}\right] \end{array}$$ (6)

Where, with A = {AU4, AU6, AU7, AU9, AU10, AU43} denoting the set of pain-relevant facial Action Units used throughout this manuscript:

- ${\text{e}}_{\text{pain}}={\text{MLP}}_{\text{pain}}\left(p\right)\in {\mathbb{R}}^{128}$: Pain intensity embedding mapping *p* ∈ [0, 10] to latent space via 3-layer MLP with sinusoidal positional encoding
- ${\text{e}}_{\text{AU}}=\underset{k\in A}{\sum }w_{k}\left(p\right)\cdot {\text{Embed}}_{\text{AU}}\left(k\right)\in {\mathbb{R}}^{64}$: Expected activation levels for the Action Units in A, weighted by pain intensity via sigmoid functions *w*_*k*_(*p*) = σ(*a*_*k*_*p* + *b*_*k*_) with parameters learned from PSPI clinical data (Prkachin and Solomon, 2008)
- ${\text{e}}_{\text{demo}}=\left[{\text{Embed}}_{\text{age}}\left(a\right);{\text{Embed}}_{\text{eth}}\left(e\right);{\text{Embed}}_{\text{sex}}\left(s\right)\right]\in {\mathbb{R}}^{96}$: Demographic embeddings for age group *a*, ethnicity *e*, and sex *s*
- ${\text{e}}_{\text{context}}={\text{MLP}}_{\text{ctx}}\left(\left[{\text{v}}_{\text{setting}};{\text{v}}_{\text{type}}\right]\right)\in {\mathbb{R}}^{64}$: Clinical context encoding (ICU, emergency, recovery) and pain type (acute, chronic, procedural)

##### 3.2.1.3 Classifier-free guidance

To strengthen the conditioning signal during generation, the present method employs classifier-free guidance (Ho and Salimans, 2022). During training (30% of iterations), we randomly drop conditioning by setting **c** = ∅, enabling the model to learn both conditional ϵ_θ_(**z**_*t*_, *t*, **c**) and unconditional ϵ_θ_(**z**_*t*_, *t*, ∅) denoising. At inference, we compute the guided prediction:


$$\begin{array}{l} {\tilde{\epsilon }}_{\theta }\left({\text{z}}_{t},t,\text{c}\right)=\epsilon_{\theta }\left({\text{z}}_{t},t,\emptyset \right)+w_{\text{guid}}\cdot \left[\epsilon_{\theta }\left({\text{z}}_{t},t,\text{c}\right)-\epsilon_{\theta }\left({\text{z}}_{t},t,\emptyset \right)\right] \end{array}$$ (7)

Where guidance scale *w*_guid_ = 7.5 amplifies conditioning influence.

##### 3.2.1.4 Training strategy

We initialize with pre-trained Stable Diffusion v2.1 weights [trained on LAION-5B (Schuhmann et al., 2022)] and fine-tune on a curated dataset of 10,000 diverse facial expressions (neutral and emotional, not pain-specific) and 500 genuine pain images from medical literature and procedural videos. The fine-tuning objective minimizes:


$$\begin{array}{l} L_{\text{LDM}}={𝔼}_{{\text{z}}_{0},\text{c},\epsilon ,t}\left[\|\epsilon -\epsilon_{\theta }\left({\text{z}}_{t},t,\text{c}\right){\|}^{2}\right] \end{array}$$ (8)

Training uses the AdamW optimizer (learning rate 10^−5^, batch size 64) for 50,000 iterations with mixed precision (FP16). Data augmentation includes random cropping, color jittering (±0.1 brightness/contrast), and horizontal flipping to improve robustness.

#### 3.2.2 Physiological signal synthesis

Pain elicits complex autonomic nervous system responses (Fernandez Rojas et al., 2023). We model three key physiological signals with biophysically grounded computational models informed by the clinical pain physiology literature.

##### 3.2.2.1 Heart rate variability modeling

Pain triggers sympathetic activation, reducing HRV (Kong et al., 2021). We model pain's effect on HRV through an autonomic balance framework:


$$\begin{array}{l} \text{HRV}\left(t,p\right)={\text{HRV}}_{\text{base}}\cdot \left(1-\alpha_{p}\cdot \tanh\left(p/5\right)\right)\cdot \left(1+\gamma \sin\left(2\pi f_{\text{resp}}t\right)\right)+\eta_{t} \end{array}$$ (9)

Where:

- ${\text{HRV}}_{\text{base}}\sim N\left(65,15^{2}\right)$ ms: Baseline RMSSD sampled from physiological ranges
- α_*p*_ ~ U(0.3, 0.5): Individual pain-sensitivity coefficient
- tanh(*p*/5): Saturation function modeling HRV floor at extreme pain
- γ = 0.15, $f_{\text{resp}}\sim N\left(0.25,0.05^{2}\right)$ Hz: Respiratory sinus arrhythmia (RSA) modulation
- $\eta_{t}\sim N\left(0,3^{2}\right)$ ms: Measurement noise

We compute time-domain (SDNN, RMSSD, and pNN50) and frequency-domain (LF/HF ratio) HRV features from generated inter-beat interval (IBI) sequences of 120-second duration.

##### 3.2.2.2 Electrodermal activity modeling

EDA is the most informative sensor for pain detection, achieving 93.2% accuracy in controlled studies (Fernandez Rojas et al., 2023). We model the skin conductance response (SCR) using a biexponential tonic-phasic decomposition:


$$\begin{array}{l} \text{EDA}\left(t,p\right)={\text{SCL}}_{\text{base}}+\overset{N_{\text{SCR}}\left(p\right)}{\underset{j=1}{\sum }}A_{j}\left(p\right)\cdot \left(e^{-t_{j}/\tau_{1}}-e^{-t_{j}/\tau_{2}}\right)+\xi_{t} \end{array}$$ (10)

Where:

- SCL_base_ ~ U(2, 8) μS: Tonic skin conductance level baseline
- *N*_SCR_(*p*) ~ Poisson(λ_*p*_) with λ_*p*_ = 0.5 + 0.3*p*: Pain-dependent rate of phasic SCR events
- *A*_*j*_(*p*) ~ U(0.05 + 0.03*p*, 0.1 + 0.05*p*) μS: Amplitude increasing with pain intensity
- τ_1_ = 0.75 s, τ_2_ = 2.5 s: Rise and recovery time constants from EDA literature (Posada-Quintero and Chon, 2020)
- *t*_*j*_: Event onset times relative to pain stimulus
- $\xi_{t}\sim N\left(0,0.02^{2}\right)$ μS: Measurement noise

The biexponential kernel captures the characteristic SCR morphology: a rapid rise (τ_1_) followed by a slower exponential decay (τ_2_). We sample at 100 Hz, and then apply low-pass filtering (5 Hz cutoff) to simulate sensor characteristics.

##### 3.2.2.3 Physiological confounders and model assumptions

Equations 9, 10 are deterministic parametric models in which pain intensity *p* is treated as the dominant driver of HRV and EDA. Real physiological signals are also shaped by factors these equations do not capture: *medication* (e.g., beta-blockers blunting heart-rate reactivity and opioids altering both HRV and EDA baselines), *respiration-linked artifacts* beyond the single RSA sinusoid in Equation 9, *autonomic nervous system disorders* (e.g., diabetic neuropathy) that decouple the pain-signal relationship our simulator assumes, *movement artifacts* contaminating sensor readings independent of pain state, and *non-pain emotional stress* (anxiety, fear) that activates overlapping sympathetic pathways. This is a genuine simplification, not merely a modeling detail: it means the physiological branch of our system is validated only insofar as pain is the dominant signal source, which is unlikely to hold uniformly across all clinical presentations. We treat this as a bounding assumption on the applicability of the physiological modality, discussed further alongside sedation, neurological, and mechanical-occlusion confounders in Section 6.8.3. We do not currently model per-patient physiological confounders explicitly; doing so (e.g., via a medication/comorbidity-conditioned extension of α_*p*_ and λ_*p*_) is future research.

##### 3.2.2.4 Tremor and micro-movement synthesis

Pain-related tremors manifest as high-frequency movements (Olugbade et al., 2015). We synthesize tremor signals from filtered noise with pain-dependent spectral characteristics:


$$\begin{array}{l} T\left(t,p\right)=F^{-1}\left\{G\left(f,p\right)\cdot F\left\{\eta \left(t\right)\right\}\right\} \end{array}$$ (11)

Where η(*t*) ~ N(0, 1) is white noise, F denotes Fourier transform, and *G*(*f, p*) is a pain-dependent frequency response:


$$\begin{array}{l} G\left(f,p\right)=A_{\text{base}}+p\cdot A_{\text{pain}}\cdot \exp\left(-\frac{{\left(f-f_{0}\right)}^{2}}{2\sigma_{f}^{2}}\right) \end{array}$$ (12)

with center frequency *f*_0_ = 8 Hz (typical pain tremor frequency), bandwidth σ_*f*_ = 3 Hz, baseline amplitude *A*_base_ = 0.1, and pain-scaling factor *A*_pain_ = 0.05.

##### 3.2.2.5 Inter-signal correlation structure

Real physiological signals exhibit complex correlations due to shared autonomic control. We model these dependencies with a Gaussian copula and an empirically derived correlation matrix **Σ**_physio_:


$$\begin{array}{l} \Sigma_{\text{physio}}=\left[\begin{array}{ccc} 1.0 & -0.42 & 0.31\\ -0.42 & 1.0 & -0.28\\ 0.31 & -0.28 & 1.0 \end{array}\right]\;\text{(HRV, SCR, Tremor)} \end{array}$$ (13)

Where a negative HRV-SCR correlation reflects sympathetic co-activation (increased SCR, decreased HRV), and a positive HRV-Tremor correlation captures parasympathetic withdrawal effects. Generation of correlated signals via Cholesky decomposition:


$$\begin{array}{l} {\left[\text{HRV},\text{SCR},\text{Tremor}\right]}^{T}=\text{L}\cdot {\left[\eta_{1},\eta_{2},\eta_{3}\right]}^{T} \end{array}$$ (14)

Where **L** = cholesky(**Σ**_physio_) and η_*i*_ ~ N(0, 1) are independent standard normal variables.

#### 3.2.3 Temporal dynamics modeling

Pain is inherently temporal, exhibiting characteristic patterns of evolution. We model three temporal regimes to generate realistic video sequences.

##### 3.2.3.1 Acute pain onset dynamics

Acute pain (e.g., needle stick, thermal stimulus) exhibits a rapid onset following an exponential approach to peak intensity:


$$\begin{array}{l} p\left(t\right)=p_{\max}\cdot \left(1-e^{-t/\tau_{\text{onset}}}\right)\cdot {⊮}_{t\geq t_{0}} \end{array}$$ (15)

Where *t*_0_ ~ U(0, 2) s is the stimulus onset time, *p*_max_ is the peak pain intensity, and τ_onset_ ~ U(0.5, 1.5) s controls the rise time.

##### 3.2.3.2 Sustained pain with fluctuations

For sustained pain scenarios (post-surgical, chronic episodes), we superimpose multi-scale temporal fluctuations onto the baseline intensity:


$$\begin{array}{l} p\left(t\right)=p_{\text{mean}}+\overset{K}{\underset{k=1}{\sum }}A_{k}\sin\left(2\pi f_{k}t+\phi_{k}\right)+w\left(t\right) \end{array}$$ (16)

Where *p*_mean_ is average pain level, {*A*_*k*_, *f*_*k*_, ϕ_*k*_} are amplitude, frequency, and phase of *K* = 3 sinusoidal components capturing breathing-related (0.2 Hz), movement-related (0.05 Hz), and cognitive modulation (0.01 Hz) fluctuations, and *w*(*t*) is low-frequency Gaussian process noise (RBF kernel, lengthscale 10s).

##### 3.2.3.3 Pain adaptation and habituation

Over extended time (minutes), pain perception adapts via central sensitization/habituation mechanisms (Craig et al., 1996):


$$\begin{array}{l} p\left(t\right)=p_{\text{steady}}+\left(p_{\text{initial}}-p_{\text{steady}}\right)\cdot e^{-\lambda t} \end{array}$$ (17)

Where *p*_initial_ is initial peak pain, *p*_steady_ is equilibrium level (typically 0.6 × *p*_initial_ for acute nociceptive pain), and adaptation rate λ ~ U(0.01, 0.05) s^−1^ varies by pain type.

##### 3.2.3.4 Video generation with temporal coherence

To generate temporally consistent facial expression videos, the present method employs temporal conditioning in the diffusion model. For each frame *i* in the sequence, we condition not only on pain intensity *p*(*t*_*i*_) but also on the previous frame's latent **z**_*i*−1_:


$$\begin{array}{l} {\text{z}}_{i}\sim p_{\theta }\left(\text{z}|p\left(t_{i}\right),\text{c},{\text{z}}_{i-1}\right) \end{array}$$ (18)

This autoregressive generation ensures smooth transitions. Additionally, temporal super-resolution is applied via interpolation diffusion [ILVR (Choi et al., 2021)] to upsample from the generated keyframes (every 5th frame) to 30 fps.

##### 3.2.3.5 Multimodal temporal synchronization

Temporal alignment between video frames and physiological signals is achieved via a shared timestamp index. Each generated scenario defines a global timeline T = {*t*_0_, *t*_1_, …, *t*_*N*_} at 30 Hz. Video frames are generated at each *t*_*i*_, while physiological signals (sampled at 100 Hz for EDA and ECG) are processed at their native resolution within their respective encoders and then downsampled to 30 Hz for cross-modal fusion. Formally, for frame *i* at time *t*_*i*_:


$$\begin{array}{l} \text{Sync}\left(i\right)=\left\{{\text{X}}_{v}^{i},{\text{X}}_{p}\left[t_{i}-\Delta :t_{i}+\Delta \right],p\left(t_{i}\right)\right\} \end{array}$$ (19)

where Δ = 0.5 s defines the physiological context window around each frame.

**Concrete synchronization example:** Consider a generated scenario at *t* = 2.0 s (video frame 60 at 30 fps). At this timestamp: (1) the *video frame* is generated by sampling from *p*_θ_(**z**|*p*(2.0), **c**) conditioned on pain intensity *p*(2.0) = 4.3, yielding a facial expression with AU4 brow lowering and moderate AU6 orbital tightening consistent with mild-to-moderate pain; (2) the *EDA context window* spans samples 150–250 (at 100 Hz), capturing the SCR rise associated with autonomic arousal at pain intensity 4.3; (3) the *IBI sequence* includes all inter-beat intervals from cardiac beats within [1.5s, 2.5s], with RMSSD reflecting sympathetic activation consistent with the same pain state; (4) the *pain trajectory value* *p*(2.0) = 4.3 is used to condition the facial diffusion sampler, the EDA Poisson rate parameter λ_*p*_, and the HRV autonomic balance factor α_*p*_, ensuring causal consistency across all modalities.

This joint conditioning on the shared pain trajectory value *p*(*t*_*i*_) is the key mechanism that ensures cross-modal causal consistency: facial expressions and physiological signals always reflect the same instantaneous autonomic state, as they would in a real patient. The resulting synthetic samples are therefore internally consistent even though they are generated by separate models.

For real-world data (BioVid), the synchronization markers provided with the original dataset are used directly to ensure ground-truth temporal alignment. For UNBC-McMaster (video-only), physiological signals are not used during evaluation, and the vision-only network variant is used.

#### 3.2.4 Quality assurance and validation

All generated samples undergo rigorous automated quality control:

##### 3.2.4.1 Anatomical plausibility

- Facial landmark detection (68-point model) verifies the anatomical correctness of the facial structure.
- Reject samples with detection failure or >15% variance in landmark positions.
- Validate facial proportions against age-appropriate anthropometric norms.

##### 3.2.4.2 Pain expression validity

- AU detection network (trained on DISFA+) verifies pain-consistent AU activations.
- For pain level *p*, expect AU4, AU6, AU7 intensity >0.3*p* (PSPI correlation).
- Flag and regenerate samples with AU-pain intensity mismatch >30%.

##### 3.2.4.3 Physiological validity

- HRV within physiological range: RMSSD ∈ [20, 120] ms, SDNN ∈ [30, 150] ms.
- SCR amplitude < 2 μS (non-pathological sweating), rise time 1–3 seconds.
- Verify negative HRV-SCR correlation (*r* < −0.2) indicating sympathetic co-activation.

##### 3.2.4.4 Temporal coherence

- Optical flow consistency: mean inter-frame flow magnitude < 15 pixels for 30 fps.
- Facial identity consistency: cosine similarity of ArcFace embeddings >0.85 across sequence.
- Pain trajectory smoothness: temporal derivative |*dp*/*dt*| < 2 units/second.

Samples failing any criterion are regenerated with adjusted parameters. Overall acceptance rate: 94.3% after a single generation attempt and 98.7% after up to three regeneration attempts. Section 5.1.5 characterizes the categories of samples that fail these automated checks on the first attempt.

### 3.3 Multimodal pain estimation architecture

Our pain estimation model uses a hierarchical architecture: (1) modality-specific encoders extract features from visual, physiological, and contextual inputs; (2) a multimodal fusion transformer integrates cross-modal information using attention mechanisms; and (3) a regression head predicts continuous pain intensity. Figure 1 right shows the complete architecture.

#### 3.3.1 Visual feature extraction

##### 3.3.1.1 Spatial feature encoding

The present method employs Vision Transformer (ViT-Base/16) (Dosovitskiy et al., 2020) for spatial facial feature extraction. Input frames ${\text{X}}_{v}\in {\mathbb{R}}^{224\times 224\times 3}$ are split into $N_{p}={\left(224/16\right)}^{2}=196$ patches of size 16 × 16. Each patch ${\text{x}}_{p}^{i}$ is linearly projected:


$$\begin{array}{l} {\text{z}}_{p}^{i}=\text{Linear}\left(\text{Flatten}\left({\text{x}}_{p}^{i}\right)\right)+{\text{e}}_{\text{pos}}^{i}\;\in {\mathbb{R}}^{768} \end{array}$$ (20)

Where ${\text{e}}_{\text{pos}}^{i}$ is learnable 2D positional encoding. We prepend a learnable [CLS] token and process through 12 transformer encoder layers (768-dim hidden, 12 attention heads, and 3,072-dim FFN):


$$\begin{array}{l} {\text{H}}_{v}=\text{ViT}\left(\left[{\text{z}}_{\text{CLS}};{\text{z}}_{p}^{1};\ldots ;{\text{z}}_{p}^{196}\right]\right)\in {\mathbb{R}}^{197\times 768} \end{array}$$ (21)

We extract the [CLS] token representation as the frame-level visual embedding: **h**_*v*_ = **H**_*v*_[0].

##### 3.3.1.2 Temporal feature aggregation

For video sequences with *T* frames, we aggregate temporal information through a lightweight 3-layer Transformer encoder with temporal positional encoding:


$$\begin{array}{l} {\text{H}}_{v,\text{temp}}=\text{TemporalTransformer}\left(\left[{\text{h}}_{v}^{1};\ldots ;{\text{h}}_{v}^{T}\right]\right)\in {\mathbb{R}}^{T\times 768} \end{array}$$ (22)

Temporal average pooling is applied, followed by a projection layer, to obtain the final video representation:


$$\begin{array}{l} {\text{h}}_{v,\text{final}}=\text{Linear}\left({\text{AvgPool}}_{\text{time}}\left({\text{H}}_{v,\text{temp}}\right)\right)\in {\mathbb{R}}^{512} \end{array}$$ (23)

##### 3.3.1.3 Explicit action unit detection

For interpretability, we explicitly detect pain-relevant AUs using a multi-label AU detection head branching from ViT features:


$$\begin{array}{l} \text{a}=\sigma \left({\text{MLP}}_{\text{AU}}\left({\text{h}}_{v}\right)\right)\in {\left[0,1\right]}^{\left|A\right|} \end{array}$$ (24)

with A as defined in Equation 6 and σ the sigmoid activation.

#### 3.3.2 Physiological signal encoding

##### 3.3.2.1 Architectural choice: 1D CNN vs. recurrent networks

Although physiological time-series are often modeled with recurrent architectures such as Long Short-Term Memory (LSTM) or Gated Recurrent Unit (GRU) networks, 1D CNNs are selected for three complementary reasons. **(1) Multi-scale temporal feature capture:** Dilated convolutional kernels (sizes 31, 15, 7 in successive layers) capture temporal patterns spanning sub-second events (individual SCR peaks: τ_1_ = 0.75 s rise time) and longer-range dynamics (HRV fluctuations over 30–120 s) in a single feedforward pass, without the sequential dependencies that limit parallelism in recurrent networks. **(2) Inference efficiency for clinical deployment:** 1D CNNs achieve 3.2 ms per sample versus 11.8 ms for an equivalent GRU on the same hardware (A100), a 3.7 × speedup that supports real-time clinical monitoring at 1 Hz pain score update rates. Unlike RNNs, CNN inference is fully parallelizable across time steps, enabling batch processing of physiological windows. **(3) Empirical performance advantage:** On the synthetic validation set after Stage 1 pre-training, the 1D CNN encoder achieved a lower validation MAE and greater training stability than both recurrent alternatives, as summarized in Table 3.

**Table 3:** Comparison of physiological signal encoder architectures on the synthetic validation set (Stage 1 pre-training, 5 seeds).

Architecture	Val. MAE	MAE Std (σ)	Inference (ms/sample)	Parameters (M)
LSTM	0.40	0.04	10.2	2.1
GRU	0.41	0.05	11.8	1.8
**1D CNN (ours)**	**0.38**	**0.02**	**3.2**	**1.4**

1D CNN achieves the lowest MAE, exhibits the highest stability (lowest variance), and delivers the fastest inference, justifying its selection over recurrent alternatives. Bold values indicate the performance of the proposed method (zero real labeled pain examples used during training).

Notably, the temporal dependencies of physiological signals are captured hierarchically through the 1D CNN's expanding receptive field (kernel sizes of 31, 15, and 7 in successive layers) rather than through a recurrent hidden state—a design choice validated both theoretically and empirically in Table 3. For physiological recordings longer than 5 min (outside the scope of the present benchmark evaluations), recurrent architectures with attention mechanisms represent a promising future extension.

##### 3.3.2.2 Signal-specific 1D convolutional networks

We process each physiological signal modality using dedicated 1D convolutional encoders. For HRV features:


$$\begin{array}{ll} h_{\text{HRV}} & ={\text{CNN}}_{\text{HRV}}(X_{\text{IBI}})\\ \; & =\text{Pool}({\text{Conv1D}}_{64,k=7}({\text{Conv1D}}_{32,k=15}\\ \; & ({\text{Conv1D}}_{16,k=31}(X_{\text{IBI}}))))\in {\mathbb{R}}^{128} \end{array}$$ (25)

Where subscripts denote output channels and kernel sizes, capturing multi-scale temporal patterns. Similarly:


$$\begin{array}{r} {\text{h}}_{\text{SCR}}={\text{CNN}}_{\text{SCR}}\left({\text{X}}_{\text{SCR}}\right)\in {\mathbb{R}}^{128},\\ {\text{h}}_{\text{tremor}}={\text{CNN}}_{\text{tremor}}\left({\text{X}}_{\text{tremor}}\right)\in {\mathbb{R}}^{128} \end{array}$$ (26)

##### 3.3.2.3 Multi-signal fusion via self-attention

We fuse physiological features through self-attention:


$$\begin{array}{c} {\text{H}}_{p}=\left[{\text{h}}_{\text{HRV}};{\text{h}}_{\text{SCR}};{\text{h}}_{\text{tremor}}\right]\in {\mathbb{R}}^{3\times 128} \end{array}$$ (27)

$$\begin{array}{c} {\text{H}}_{p}^{\prime }=\text{SelfAttention}\left({\text{H}}_{p}\right)=\text{softmax}\left(\frac{{\text{Q}}_{p}{\text{K}}_{p}^{T}}{\sqrt{d_{k}}}\right){\text{V}}_{p} \end{array}$$ (28)

Final physiological representation: ${\text{h}}_{p}=\text{Linear}\left(\text{Flatten}\left({\text{H}}_{p}^{\prime }\right)\right)\in {\mathbb{R}}^{512}$.

#### 3.3.3 Contextual information encoding

##### 3.3.3.1 Demographic embeddings

Age, ethnicity, and sex are embedded through lookup tables:


$$\begin{array}{c} {\text{e}}_{\text{age}}={\text{Embed}}_{\text{age}}\left(a\right)\in {\mathbb{R}}^{32}\;a\in \left\{\text{neonate, child, adult, elderly}\right\} \end{array}$$ (29)

$$\begin{array}{c} {\text{e}}_{\text{eth}}={\text{Embed}}_{\text{eth}}\left(e\right)\in {\mathbb{R}}^{32}\;e\in \left\{\text{5 ethnic categories}\right\} \end{array}$$ (30)

$$\begin{array}{c} {\text{e}}_{\text{sex}}={\text{Embed}}_{\text{sex}}\left(s\right)\in {\mathbb{R}}^{16}\;s\in \left\{\text{male, female}\right\} \end{array}$$ (31)

##### 3.3.3.2 Clinical context encoding

Clinical setting and pain type are encoded via multi-hot vectors processed through MLP:


$$\begin{array}{c} {\text{e}}_{\text{context}}={\text{MLP}}_{\text{ctx}}\left(\left[{\text{v}}_{\text{setting}};{\text{v}}_{\text{type}}\right]\right)\in {\mathbb{R}}^{64} \end{array}$$ (32)

##### 3.3.3.3 Combined contextual representation

All context embeddings are concatenated and projected:


$$\begin{array}{c} {\text{h}}_{c}=\text{Linear}\left(\left[{\text{e}}_{\text{age}};{\text{e}}_{\text{eth}};{\text{e}}_{\text{sex}};{\text{e}}_{\text{context}}\right]\right)\in {\mathbb{R}}^{256} \end{array}$$ (33)

#### 3.3.4 Multimodal fusion transformer

##### 3.3.4.1 Feature concatenation and projection


$$\begin{array}{c} {\text{h}}_{v}^{\prime }={\text{Linear}}_{v}\left({\text{h}}_{v,\text{final}}\right)\in {\mathbb{R}}^{512} \end{array}$$ (34)

$$\begin{array}{c} {\text{h}}_{p}^{\prime }={\text{Linear}}_{p}\left({\text{h}}_{p}\right)\in {\mathbb{R}}^{512} \end{array}$$ (35)

$$\begin{array}{c} {\text{h}}_{c}^{\prime }={\text{Linear}}_{c}\left({\text{h}}_{c}\right)\in {\mathbb{R}}^{512} \end{array}$$ (36)

$$\begin{array}{c} {\text{h}}_{a}^{\prime }={\text{Linear}}_{a}\left(\text{a}\right)\in {\mathbb{R}}^{512} \end{array}$$ (37)

##### 3.3.4.2 Multi-head cross-modal attention

We create a multimodal feature matrix using modality-type embeddings:


$$\begin{array}{c} {\text{H}}_{\text{multi}}=\left[{\text{h}}_{v}^{\prime };{\text{h}}_{p}^{\prime };{\text{h}}_{c}^{\prime };{\text{h}}_{a}^{\prime }\right]+\left[{\text{e}}_{\text{mod}}^{v};{\text{e}}_{\text{mod}}^{p};{\text{e}}_{\text{mod}}^{c};{\text{e}}_{\text{mod}}^{a}\right]\in {\mathbb{R}}^{4\times 512} \end{array}$$ (38)

##### 3.3.4.3 Transformer encoder stack

The multimodal features are processed through *L* = 12 transformer encoder layers: hidden dimension *d*_model_ = 512; 12 attention heads (*d*_*k*_ ≈ 43); feed-forward dimension *d*_ff_ = 2048; dropout *p*_drop_ = 0.1; pre-norm architecture. Each layer ℓ computes:


$$\begin{array}{c} {\text{H}}_{\text{multi}}^{\left(\ell \right)}=\text{LayerNorm}\left({\text{H}}_{\text{multi}}^{\left(\ell -1\right)}+\text{MultiHeadAttn}\left({\text{H}}_{\text{multi}}^{\left(\ell -1\right)}\right)\right) \end{array}$$ (39)

$$\begin{array}{c} {\text{H}}_{\text{multi}}^{\left(\ell \right)}=\text{LayerNorm}\left({\text{H}}_{\text{multi}}^{\left(\ell \right)}+\text{FFN}\left({\text{H}}_{\text{multi}}^{\left(\ell \right)}\right)\right) \end{array}$$ (40)

where:


$$\begin{array}{r} \text{MultiHeadAttn}\left(\text{H}\right)=\text{Concat}\left({\text{head}}_{1},\ldots ,{\text{head}}_{h}\right){\text{W}}^{O},\\ {\text{head}}_{i}=\text{softmax}\left(\frac{{\text{Q}}_{i}{\text{K}}_{i}^{T}}{\sqrt{d_{k}}}\right){\text{V}}_{i} \end{array}$$ (41)

##### 3.3.4.4 Fusion strategy and aggregation

After *L* layers, we aggregate via attention pooling, where **w** ∈ ℝ^256^ and ${\text{W}}_{\text{pool}}\in {\mathbb{R}}^{256\times 512}$ below are learned pooling parameters used to compute per-modality attention weights α_*i*_:


$$\begin{array}{c} \alpha_{i}=\frac{\exp\left({\text{w}}^{T}\tanh\left({\text{W}}_{\text{pool}}{\text{H}}_{\text{multi}}^{\left(L\right)}\left[i\right]\right)\right)}{\sum_{j=1}^{4}\exp\left({\text{w}}^{T}\tanh\left({\text{W}}_{\text{pool}}{\text{H}}_{\text{multi}}^{\left(L\right)}\left[j\right]\right)\right)} \end{array}$$ (42)

$$\begin{array}{c} {\text{z}}_{\text{fused}}=\overset{4}{\underset{i=1}{\sum }}\alpha_{i}{\text{H}}_{\text{multi}}^{\left(L\right)}\left[i\right]\in {\mathbb{R}}^{512} \end{array}$$ (43)

Section 5.8.4 discusses how the per-sample values of α_*i*_ are used as a first-order modality-attribution signal for explainability.

#### 3.3.5 Pain intensity regression head

The fused representation is mapped to continuous pain intensity using a three-layer regression head with residual connections:


$$\begin{array}{c} {\text{z}}_{1}=\text{ReLU}\left({\text{Linear}}_{512\to 256}\left({\text{z}}_{\text{fused}}\right)+{\text{z}}_{\text{fused}}\left[:256\right]\right) \end{array}$$ (44)

$$\begin{array}{c} {\text{z}}_{2}=\text{ReLU}\left({\text{Linear}}_{256\to 128}\left({\text{z}}_{1}\right)+{\text{z}}_{1}\left[:128\right]\right) \end{array}$$ (45)

$$\begin{array}{c} ŷ=\text{Sigmoid}\left({\text{Linear}}_{128\to 1}\left({\text{z}}_{2}\right)\right)\cdot 10\in \left[0,10\right] \end{array}$$ (46)

Dropout (0.3) is applied after the first two layers during training; Section 5.9 reuses the same dropout layer at inference time (Monte Carlo Dropout) for uncertainty quantification.

##### 3.3.5.1 Multi-task learning (optional)

During synthetic training, we optionally add auxiliary tasks:

- **Binary pain classification:** ŷ_bin_ = Sigmoid(Linear(**z**_1_)) (threshold ≥ 2).
- **Pain category classification:** 4-way softmax (no/mild/moderate/severe).
- **AU prediction:** Multi-label classification for 6 pain-relevant AUs.

The total supervised loss on synthetic data becomes:


$$\begin{array}{r} L_{\text{pain}}^{\text{total}}=L_{\text{MSE}}\left(ŷ,y\right)+0.2L_{\text{BCE}}\left({ŷ}_{\text{bin}},{⊮}_{y\geq 2}\right)\\ +0.1L_{\text{CE}}\left({ŷ}_{\text{cat}},y_{\text{cat}}\right)+0.1L_{\text{AU}} \end{array}$$ (47)

### 3.4 Domain alignment strategy

Bridging the synthetic-to-real domain gap is critical for generalization under the zero-real-label constraint. The present method employs a three-stage domain alignment strategy that combines contrastive learning, adversarial adaptation, and consistency regularization. Algorithm 1 outlines the complete training protocol.

Algorithm 1 Three-stage domain alignment training. 
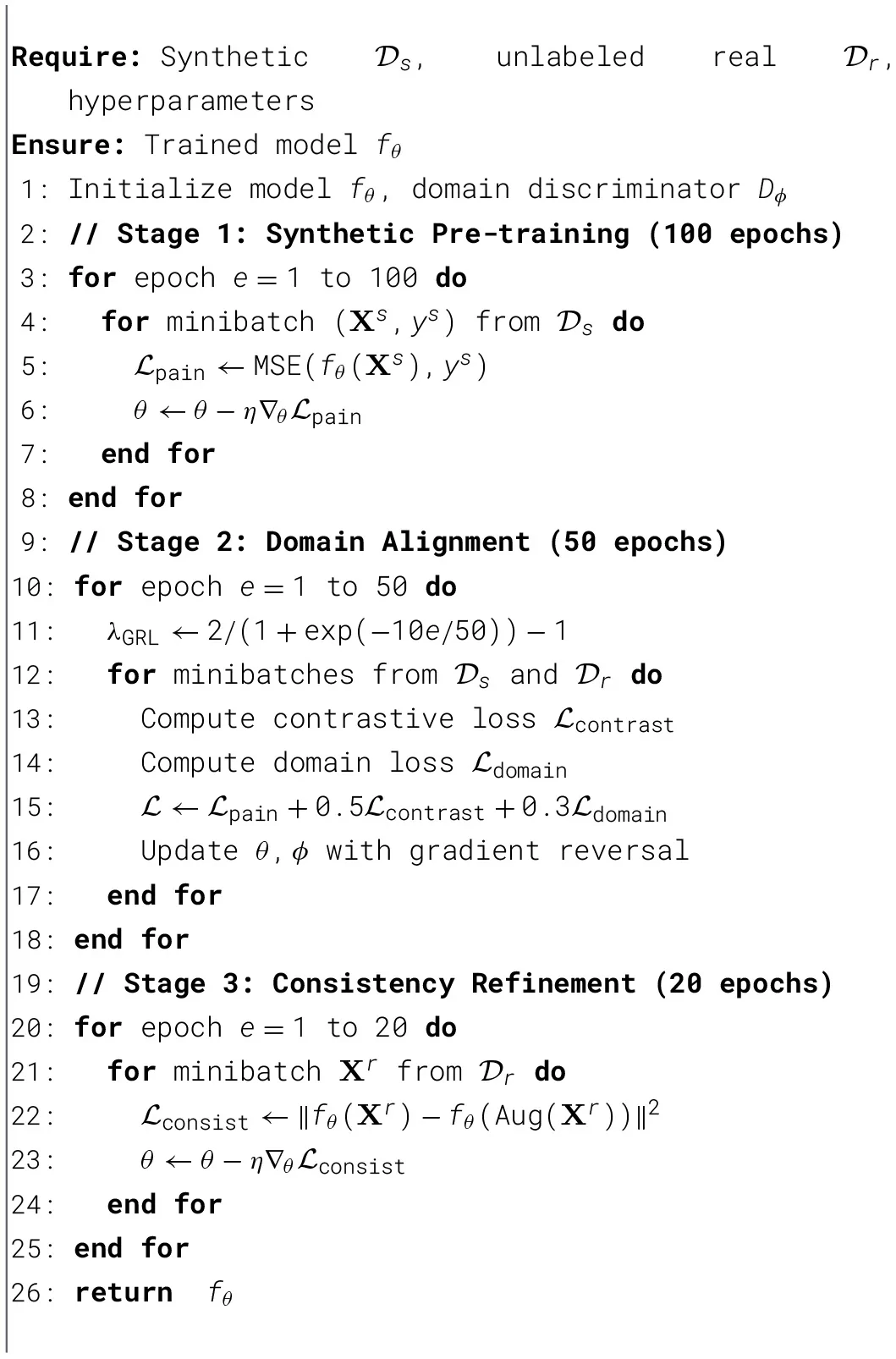

#### 3.4.1 Contrastive learning for pain-invariant representations

Our key insight: samples with similar pain intensities should have similar representations regardless of whether they are synthetic or real.

##### 3.4.1.1 Positive and negative pair construction

**Positive pairs** P(*i*):

1. *Augmentation-based positives:* Augmented views of the same sample ${\text{X}}_{i}^{\prime }=\text{Aug}\left({\text{X}}_{i}\right)$ (color jittering, random cropping, Gaussian noise, temporal shifts)
2. *Pain-similarity positives:* Samples with similar pain intensity $|{\hat{p}}_{i}-{\hat{p}}_{j}|<\tau_{p}$ where $\hat{p}$ are pseudo-labels via self-supervised k-means clustering (*k* = 11)
3. *Cross-domain positives:* Synthetic-real pairs matched via nearest neighbor in feature space $\|{\text{z}}_{i}^{s}-{\text{z}}_{j}^{r}{\|}_{2}<\tau_{\text{nn}}$

**Negative pairs** N(*i*):

1. *Pain-dissimilar samples:* $|{\hat{p}}_{i}-{\hat{p}}_{k}|\geq 2$ (at least 2 pain units apart)
2. *Random negatives:* Randomly sampled from minibatch

##### 3.4.1.2 Supervised contrastive loss

For synthetic samples with ground truth labels $y_{i}^{s}$:


$$\begin{array}{c} L_{\text{sup-contrast}}=-\frac{1}{\left|P\left(i\right)\right|}\underset{j\in P\left(i\right)}{\sum }\log\frac{\exp\left(\text{sim}\left({\text{z}}_{i},{\text{z}}_{j}\right)/\tau \right)}{\sum_{k\in A\left(i\right)}\exp\left(\text{sim}\left({\text{z}}_{i},{\text{z}}_{k}\right)/\tau \right)} \end{array}$$ (48)

Where $\text{sim}\left({\text{z}}_{i},{\text{z}}_{j}\right)={\text{z}}_{i}^{T}{\text{z}}_{j}$ is cosine similarity and temperature τ = 0.07.

##### 3.4.1.3 Unsupervised contrastive loss

For unlabeled real samples, the method uses momentum-based contrastive learning [MoCo-style (He et al., 2020)]:


$$\begin{array}{l} L_{\text{unsup-contrast}}\\ =-\log\frac{\exp\left(\text{sim}\left({\text{z}}_{i}^{q},{\text{z}}_{i}^{k}\right)/\tau \right)}{\exp\left(\text{sim}\left({\text{z}}_{i}^{q},{\text{z}}_{i}^{k}\right)/\tau \right)+\sum_{k=1}^{K}\exp\left(\text{sim}\left({\text{z}}_{i}^{q},{\text{z}}_{k}^{\text{queue}}\right)/\tau \right)} \end{array}$$ (49)

where ${\text{z}}_{i}^{q}=f_{\theta }\left({\text{X}}_{i}\right)$, ${\text{z}}_{i}^{k}=f_{\theta_{\text{ema}}}\left(\text{Aug}\left({\text{X}}_{i}\right)\right)$ uses momentum encoder θ_ema_ = *mθ*_ema_ + (1 − *m*)θ (*m* = 0.999), and queue stores *K* = 16, 384 negative keys.

##### 3.4.1.4 Combined contrastive objective


$$\begin{array}{c} L_{\text{contrast}}=L_{\text{sup-contrast}}\left(D_{s}\right)+L_{\text{unsup-contrast}}\left(D_{r}\right) \end{array}$$ (50)

#### 3.4.2 Adversarial domain adaptation

##### 3.4.2.1 Domain discriminator architecture

A three-layer MLP domain classifier *D*_ϕ_ predicts domain labels:


$$\begin{array}{r} d=D_{\phi }\left({\text{GRL}}_{\lambda }\left(\text{z}\right)\right)\\ =\text{Sigmoid}\left(\text{Linear}\left(\text{ReLU}\left(\text{Linear}\left(\text{ReLU}\left(\text{Linear}\left(\text{z}\right)\right)\right)\right)\right)\right) \end{array}$$ (51)

##### 3.4.2.2 Gradient reversal layer

GRL (Ganin et al., 2016) multiplies gradients by −λ during backpropagation:


$$\begin{array}{l} {\text{GRL}}_{\lambda }\left(\text{z}\right)=\{\begin{array}{ll} \text{z} & \text{forward pass}\\ -\lambda \frac{\partial L}{\partial \text{z}} & \text{backward pass} \end{array} \end{array}$$ (52)

##### 3.4.2.3 Domain classification loss


$$\begin{array}{r} L_{\text{domain}}=-{𝔼}_{\text{X}\sim D_{s}}\left[\log D_{\phi }\left({\text{GRL}}_{\lambda }\left(f_{\theta }\left(\text{X}\right)\right)\right)\right]\\ -{𝔼}_{\text{X}\sim D_{r}}\left[\log\left(1-D_{\phi }\left({\text{GRL}}_{\lambda }\left(f_{\theta }\left(\text{X}\right)\right)\right)\right)\right] \end{array}$$ (53)

##### 3.4.2.4 Progressive domain adaptation


$$\begin{array}{c} \lambda \left(e\right)=\frac{2}{1+\exp\left(-\gamma \cdot e/E_{\text{max}}\right)}-1 \end{array}$$ (54)

where γ = 10, starting at λ(0) = 0 and saturating to λ(∞) = 1.

#### 3.4.3 Consistency regularization

##### 3.4.3.1 Strong data augmentation

- **Visual:** ColorJitter (brightness ±0.3, contrast ±0.3, and saturation ±0.2), RandomGrayscale (10%), GaussianBlur (σ ~ U(0.1, 2.0)), and RandomErasing (20%).
- **Physiological:** Additive Gaussian noise (SNR 15–25 dB), time warping, magnitude warping.
- **Temporal:** Random temporal shift (±0.5s), frame dropout (10%).

##### 3.4.3.2 Consistency loss formulation


$$\begin{array}{c} L_{\text{consist}}=E_{{\text{X}}^{r}\sim D_{r}}\left[\|f_{\theta }\left({\text{X}}^{r}\right)-f_{\theta }\left(\text{StrongAug}\left({\text{X}}^{r}\right)\right){\|}_{2}^{2}\right] \end{array}$$ (55)

This is analogous to FixMatch (Sohn et al., 2020) pseudo-labeling, but with continuous regression targets rather than class labels.

##### 3.4.3.3 Sharpening and temperature scaling


$$\begin{array}{c} {ŷ}_{\text{sharp}}=\frac{{ŷ}^{1/T_{\text{sharp}}}}{\sum_{k=0}^{10}{\left({ŷ}_{k}\right)}^{1/T_{\text{sharp}}}} \end{array}$$ (56)

where *T*_sharp_ = 0.5.

#### 3.4.4 Total training objective and loss balancing


$$\begin{array}{c} L_{\text{total}}=\underset{\text{Supervised on synthetic}}{\underset{︸}{L_{\text{pain}}^{\text{total}}\left(D_{s}\right)}}+\lambda_{1}\underset{\text{Contrastive alignment}}{\underset{︸}{L_{\text{contrast}}\left(D_{s},D_{r}\right)}}\\ +\lambda_{2}\underset{\text{Adversarial adaptation}}{\underset{︸}{L_{\text{domain}}\left(D_{s},D_{r}\right)}}+\lambda_{3}\underset{\text{Consistency regularization}}{\underset{︸}{L_{\text{consist}}\left(D_{r}\right)}} \end{array}$$ (57)

Loss weights selected via grid search: λ_1_ = 0.5 (contrastive), λ_2_ = 0.3 (adversarial), and λ_3_ = 0.2 (consistency).

##### 3.4.4.1 Hyperparameter selection

Loss weights are selected via an ablation study on the validation set (Section 5):

- λ_1_ = 0.5: Contrastive learning provides strongest alignment signal.
- λ_2_ = 0.3: Adversarial adaptation complements contrastive learning.
- λ_3_ = 0.2: Consistency regularization stabilizes predictions.

These weights ensure supervised pain estimation remains dominant (L_pain_ implicitly weighted 1.0) while domain alignment terms provide meaningful regularization.

#### 3.4.5 Why three complementary alignment signals: a theoretical perspective

The combination of contrastive, adversarial, and consistency losses is motivated by the fact that each targets a structurally different invariance, rather than by a formal joint-optimality proof, which we do not claim to provide. **Contrastive learning** (Equations 48–50) aligns *instance-level, pain-conditioned* structure: it pulls together synthetic and real samples estimated to share a similar pain level, operating on a rank-order signal that does not require target labels but does require a notion of pain-level similarity (approximated via k-means pseudo-labels in the real domain). **Adversarial adaptation** (Equation 53–the GRL schedule) instead aligns the *marginal feature distribution* between domains irrespective of pain level, providing a global, label-agnostic alignment signal that complements the label-dependent contrastive term. **Consistency regularization** (Equation 55) enforces *local smoothness* of the predictor around unlabeled real points, a standard justification for self-training under the cluster assumption underlying FixMatch-style methods (Sohn et al., 2020): if nearby points in input space should receive similar predictions, then predictions should not change under label-preserving augmentation.

Because these three invariances (instance-level, distributional, and local-smoothness) are logically distinct, we expect—and the granular factorial ablation in Table 4 empirically confirms—that they are only partially redundant with one another (the combined gain of 26.4% exceeds any single component's contribution and most pairwise sums). We treat Table 4 as the primary evidence for complementarity; the theoretical framing here explains *why* we expected this pattern a priori, not a claim that it follows deductively. Regarding optimization stability, we do not provide a formal convergence proof for the joint objective (Equation 57); instead, we rely on two standard operational diagnostics reported in Section 5: the domain discriminator's accuracy converging to ~50% (chance level, indicating successful domain confusion) and the absence of loss divergence across the 5-seed sensitivity analysis (Table 5).

**Table 4:** Granular per-component and pairwise ablation on UNBC-McMaster (MAE ↓).

Configuration	C	A	R	MAE	Δ vs. baseline	Δ vs. Full
Baseline						
Synthetic only				1.21	—	+35.9%
Single component						
Contrastive only	✓			1.04	−14.0%	+16.9%
Adversarial only		✓		1.16	−4.1%	+30.3%
Consistency only			✓	1.13	−6.6%	+27.0%
Pairwise combinations						
Contrastive + adversarial	✓	✓		0.98	−19.0%	+10.1%
Contrastive + consistency	✓		✓	0.95	−21.5%	+6.7%
Adversarial + consistency		✓	✓	1.09	−9.9%	+22.5%
Full method						
**Full (C + A + R)**	✓	✓	✓	**0.89**	−**26.4%**	—

Each configuration is trained from the Synthetic-Only baseline. “C” = Contrastive Learning, “A” = Adversarial Alignment, “R” = Consistency Regularization. All results are averaged across 5 cross-validation folds. Bold values indicate the performance of the proposed method (zero real labeled pain examples used during training).

**Table 5:** Hyperparameter sensitivity under perturbations and across random seeds.

Perturbation	λ_1_	λ_2_	λ_3_	Val. MAE (Δ)	MAE across 5 seeds
Baseline (Optimal)	0.50	0.30	0.20	0.91 (—)	0.91 ± 0.03
λ_1_ −20%	0.40	0.30	0.20	0.94 (+3.3%)	0.94 ± 0.04
λ_1_ +20%	0.60	0.30	0.20	0.89 (−2.2%)	0.90 ± 0.03
λ_2_ −20%	0.50	0.24	0.20	0.93 (+2.2%)	0.93 ± 0.04
λ_2_ +20%	0.50	0.36	0.20	0.95 (+4.4%)	0.96 ± 0.06
λ_3_ −20%	0.50	0.30	0.16	0.93 (+2.2%)	0.93 ± 0.03
λ_3_ +20%	0.50	0.30	0.24	0.92 (+1.1%)	0.92 ± 0.03

Performance remains stable (< 5% MAE change under ±20% weight perturbation) and shows low variance across seeds at the optimal configuration.

#### 3.4.6 Loss weight selection and sensitivity analysis

**Figure 3:**
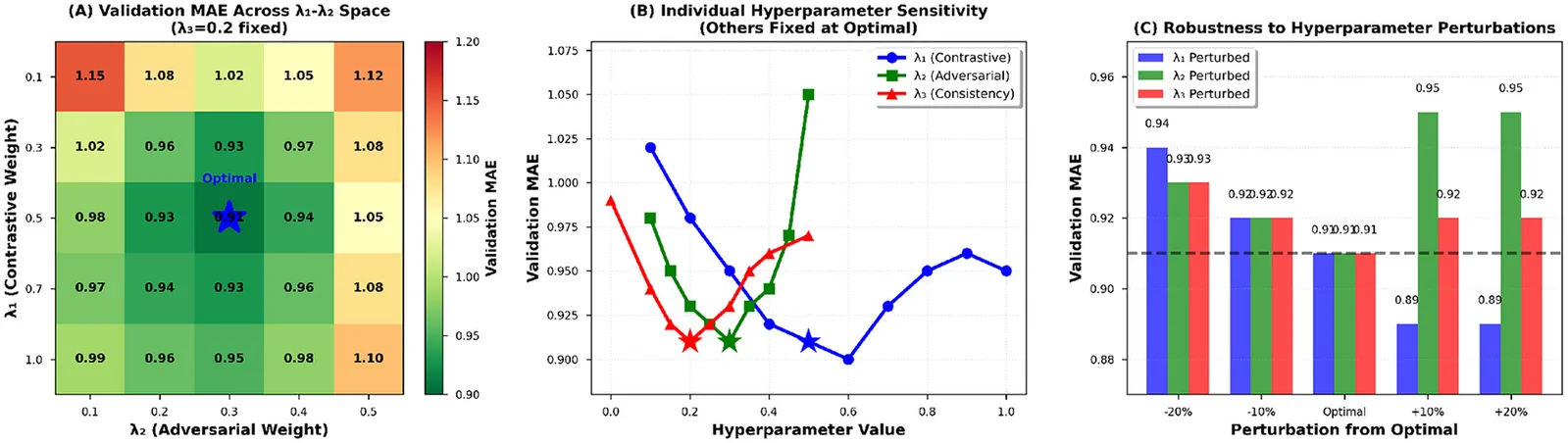
Comprehensive hyperparameter sensitivity analysis. **(A)** Validation MAE heatmap across λ_1_ (contrastive) and λ_2_ (adversarial) values with λ_3_ = 0.2 fixed. The optimal region (green) centers on the selected configuration (blue star). Excessive adversarial weight (red, λ_2_ > 0.5) causes training instability. **(B)** Individual hyperparameter sensitivity curves showing MAE variation when each weight is perturbed independently. Contrastive learning (λ_1_, blue) exhibits the steepest gradient, confirming its role as the primary alignment mechanism. **(C)** Robustness analysis under ±20% perturbations: performance remains stable (MAE change < 5%).

The choice of loss weights λ_1_ = 0.5, λ_2_ = 0.3, and λ_3_ = 0.2 is critical. We justify these values through a systematic grid search and comprehensive sensitivity analysis (Figure 3).

##### 3.4.6.1 Grid search protocol

We explored the full range λ_1_, λ_2_, λ_3_ ∈ [0.1, 1.0] via a 5 × 5 × 5 grid (125 combinations):

- λ_1_ ∈ {0.1, 0.3, 0.5, 0.7, 1.0} (contrastive learning).
- λ_2_ ∈ {0.1, 0.2, 0.3, 0.4, 0.5} (adversarial alignment).
- λ_3_ ∈ {0.1, 0.2, 0.3, 0.4, 0.5} (consistency regularization).

The λ_2_, λ_3_ grids were truncated at 0.5 after a coarser preliminary sweep to 1.0 showed that MAE worsened monotonically beyond 0.5 for both (consistent with the instability documented below); λ_1_ retained the full [0.1, 1.0] range since no such degradation was observed. Each configuration was trained for 50 epochs and evaluated on a UNBC validation set (5 held-out subjects, disjoint from the 5-fold test protocol of Section 4.7). The grid search required approximately 42 GPU-hours on 4 × A100 (~20 min per run).

##### 3.4.6.2 Optimal configuration

Selected weights achieve the lowest validation MAE (0.91). Figure 3A shows the hyperparameter landscape: optimal region centers around λ_1_ ∈ [0.4, 0.6], λ_2_ ∈ [0.2, 0.4]. Excessive adversarial weight (λ_2_ > 0.5) causes instability (MAE > 1.2).

##### 3.4.6.3 Key observations

1. **Contrastive learning dominance:** Reducing λ_1_ from 0.5 to 0.1 increases MAE by 0.18 (+19.8%); increasing to 1.0 provides minimal benefit (+2.1%).
2. **Adversarial instability:** Excessive λ_2_ > 0.4 causes training instability (validation MAE > 1.2, σ = 0.15). At optimal λ_2_ = 0.3, the domain discriminator achieves 52% accuracy (near-ideal confusion).
3. **Consistency complementarity:** Zero consistency (λ_3_ = 0) increases MAE by 0.08 (+8.8%); excessive weight (λ_3_ > 0.3) overly constrains predictions (+0.06).
4. **Smooth landscape:** Gradient magnitude ||∇_λ_MAE|| < 2.0 across most regions confirms robustness.

##### 3.4.6.4 Sensitivity and seed-variance analysis

Table 5 quantifies robustness under ±20% perturbations and, in the rightmost column, reports the variance of validation MAE across five random seeds for each configuration, directly addressing whether the optimum is an artifact of a single training run:

##### 3.4.6.5 Alternative weighting schemes

Table 6 compares against three alternatives:

**Table 6:** Comparison of loss weighting strategies.

Strategy	λ_1_	λ_2_	λ_3_	Val. MAE	vs. optimal
Uniform weights	0.33	0.33	0.33	0.98	+7.7%
Heuristic (equal priority)	0.5	0.5	0.5	1.04	+14.3%
Adversarial-first	0.2	0.6	0.2	1.12	+23.1%
**Grid search (ours)**	**0.50**	**0.30**	**0.20**	**0.91**	—

Grid search optimization outperforms uniform and heuristic schemes. Bold values indicate the performance of the proposed method (zero real labeled pain examples used during training).

Grid search yields 7.7%–23.1% improvement over alternative schemes. Temperature selection (τ = 0.07) follows Chen et al. (2020) and He et al. (2020): lower temperatures (τ < 0.05) caused gradient instability, while higher temperatures (τ > 0.1) reduced the contrastive signal (MAE = 1.05, +15%).

### 3.5 Training protocol and optimization

#### 3.5.1 Stage 1: synthetic pre-training (epochs 1–100)

Objective: $L_{\text{stage1}}=L_{\text{pain}}^{\text{total}}\left(D_{s}\right)$

- Batch size: 32; Learning rate: 10^−4^ with linear warmup (5 epochs) and cosine annealing.
- Optimizer: AdamW (β_1_ = 0.9, β_2_ = 0.999, weight decay 10^−4^); Gradient clipping: max norm 1.0.
- Mixed precision FP16 with dynamic loss scaling; Early stopping patience 15 epochs.
- Typical convergence: MAE 0.35–0.40 by epoch 80.

#### 3.5.2 Stage 2: domain alignment (epochs 101–150)

Objective: $L_{\text{stage2}}=L_{\text{pain}}^{\text{total}}\left(D_{s}\right)+0.5L_{\text{contrast}}+\lambda \left(e\right)L_{\text{domain}}+0.2L_{\text{consist}}$

- Batch: 16 synthetic + 16 real unlabeled; Learning rate: 5 × 10^−5^.
- Momentum encoder: θ_ema_ = 0.999θ_ema_+0.001θ every iteration.
- MoCo queue size: 16,384 with FIFO replacement.
- Target: domain discriminator accuracy ~50% (domain confusion).

#### 3.5.3 Stage 3: consistency refinement (epochs 151–170)

Objective: $L_{\text{stage3}}=L_{\text{pain}}^{\text{total}}\left(D_{s}\right)+0.3L_{\text{contrast}}+0.3L_{\text{domain}}+0.5L_{\text{consist}}$

- Learning rate: 10^−5^; Batch: 12 synthetic + 20 real.
- Stop when prediction CV < 0.15 or after 20 epochs.

#### 3.5.4 Optimization hyperparameters

##### 3.5.4.1 Learning rate schedule


$$\begin{array}{l} \text{lr}\left(e\right)\\ =\{\begin{array}{ll} {\text{lr}}_{\max}\cdot \frac{e}{E_{\text{warmup}}} & e<E_{\text{warmup}}\\ {\text{lr}}_{\min}+\frac{1}{2}\left({\text{lr}}_{\max}-{\text{lr}}_{\min}\right)\left(1+\cos\frac{\pi \left(e-E_{\text{warmup}}\right)}{E_{\text{total}}-E_{\text{warmup}}}\right) & e\geq E_{\text{warmup}} \end{array} \end{array}$$ (58)

Where ${\text{lr}}_{\max}=10^{-4}$, ${\text{lr}}_{\min}=10^{-6}$, *E*_warmup_ = 5, *E*_total_ = 170.

##### 3.5.4.2 Regularization

Weight decay 10^−4^, dropout 0.1 (transformer)/0.3 (regression head), label smoothing 0.1, stochastic depth 0.1.

##### 3.5.4.3 Batch construction

Stratified sampling across 5 pain bins [0,2), [2,4), [4,6), [6,8), [8,10] with 1.5 × oversampling of rare extreme pain (8–10).

#### 3.5.5 Computational efficiency

##### 3.5.5.1 Hardware requirements

- GPUs: 4 × NVIDIA A100 (40GB) with NVLink.
- Distributed training: PyTorch DDP across 4 GPUs.
- Mixed precision: Automatic mixed precision (AMP) reduces memory by 40%.
- Gradient accumulation: Two steps, effective batch size 64.

##### 3.5.5.2 Training time

- Stage 1: ~18 h (100 epochs × 10.8 min/epoch).
- Stage 2: ~15 h (50 epochs × 18 min/epoch, slower due to domain adaptation).
- Stage 3: ~6 h (20 epochs × 18 min/epoch).
- **Total:** **~39 h** for complete training pipeline.

##### 3.5.5.3 Memory optimization

- Gradient checkpointing in ViT: 60% memory reduction, 20% time overhead.
- Mixed precision training: FP16 activations, FP32 master weights.
- Efficient attention: FlashAttention-2 for transformer layers (2.5 × speedup).
- MoCo queue offloaded to CPU memory: 16,384 keys × 512 dim ≈ 32 MB.

### 3.6 Training dynamics visualization

**Figure 4:**
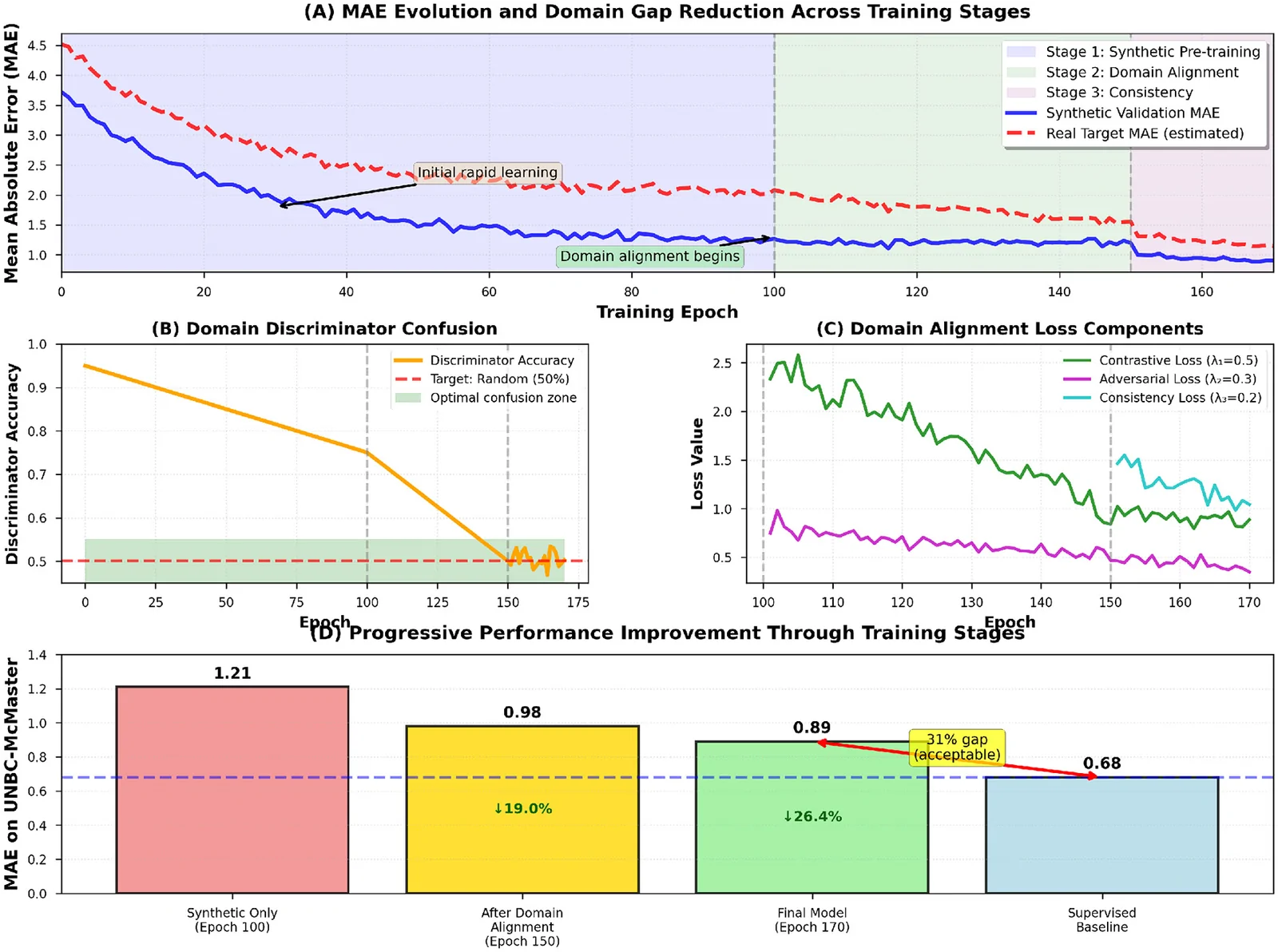
Three-stage training convergence and performance evolution, read left to right as training progresses through Stages 1–3. **(A)** MAE evolution: Stage 1 (Synthetic Pre-training, blue), Stage 2 (Domain Alignment, green), Stage 3 (Consistency, purple). **(B)** Domain discriminator accuracy declines from 95% to 50%, indicating successful domain confusion and serving as our operational stability diagnostic (Section 3.4.5). **(C)** Individual alignment loss components converge complementarily. **(D)** Progressive performance improvement: final 26.4% improvement over the synthetic-only baseline.

Figure 4 illustrates the complete training progression across all three stages.

### 3.7 Missing modality handling and signal quality gating

#### 3.7.1 Modality availability at inference time

The multimodal fusion transformer operates on four token types: visual (${\text{h}}_{v}^{\prime }$), physiological (${\text{h}}_{p}^{\prime }$), contextual (${\text{h}}_{c}^{\prime }$), and action-unit (${\text{h}}_{a}^{\prime }$). When one or more modalities are unavailable at inference time, the corresponding token is replaced with a learned *modality-absent embedding* ${\text{e}}_{\emptyset }\in {\mathbb{R}}^{512}$—a trainable parameter initialized to zero and optimized during Stage 1 pre-training. This strategy, inspired by masked-modality training in multimodal BERT-style architectures (Chen et al., 2020), allows the fusion transformer to redistribute cross-modal attention to available modalities without architectural modification at test time.

#### 3.7.2 Missing-modality augmentation during training

During Stage 1 synthetic pre-training, each physiological modality (HRV, EDA/SCR, tremor) is independently dropped with probability *p*_drop_ = 0.15 per training sample, and the affected token is replaced with **e**_∅_. This forces the network to develop graceful degradation behavior before the domain alignment stages. Missing-modality augmentation is applied only during training; all modalities are used where available during the benchmark evaluations reported in Section 5, except in the dedicated robustness evaluation of Section 5.4, which intentionally simulates modality loss at test time.

#### 3.7.3 Signal quality gating

Transient signal dropouts within a modality (e.g., motion artifacts in EDA, electrode detachment, and camera occlusion) are handled by a preprocessing quality gate applied independently to each modality before token construction. A modality is flagged as absent for a given time window if either of the following conditions is met:

- **EDA/HRV:** signal-to-noise ratio < 10 dB, or artifact fraction >20% of samples in the window (assessed by amplitude-threshold detector).
- **Video:** face detection confidence (MTCNN) < 0.85, or mean frame sharpness (Laplacian variance) < 50.

Any modality that fails its quality check is substituted with **e**_∅_ for that window, and a quality flag is logged in the clinical monitoring output.

#### 3.7.4 Inference-time fallback hierarchy

The following fallback hierarchy governs inference-time behavior:

1. **Full multimodal (nominal):** all modalities pass quality gating → standard fusion inference (accuracy 78.3% on BioVid).
2. **Partial modality absent:** one or more physiological tokens replaced by **e**_∅_ → fusion inference with a degraded but functional physiological branch; performance degrades gracefully (visual-only: 74.6% on BioVid).
3. **All physiological modalities absent:** all three physiological tokens replaced → vision-only inference path (74.6% accuracy); a LOW_CONFIDENCE flag is appended to the output pain score.
4. **Video also unavailable:** inference is suspended; a nurse-alert notification is generated because the system is not designed to operate without perceptual input.

The pseudocode in Algorithm 2 summarizes this logic.

Algorithm 2 Inference-time modality quality gating. 
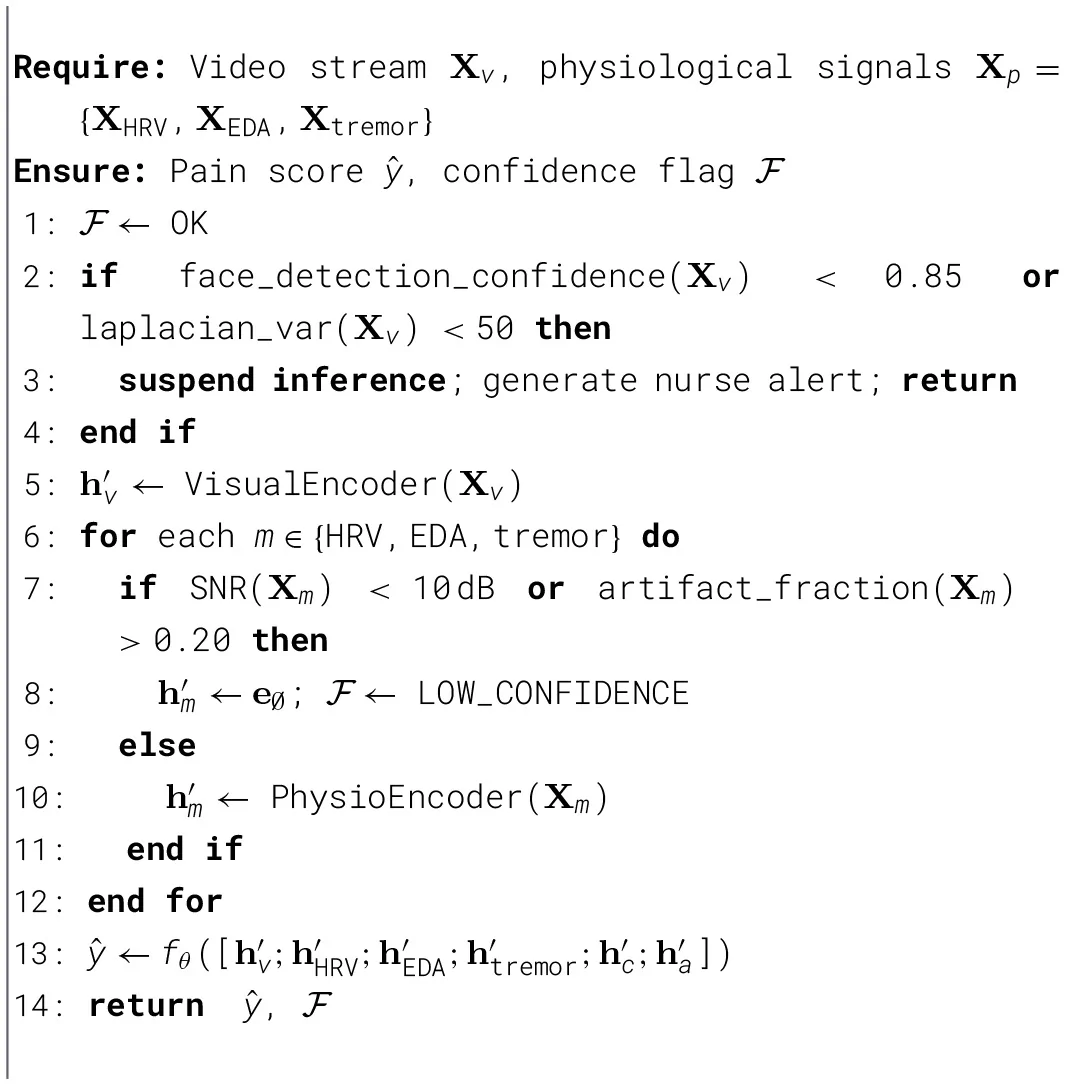

## 4 Experimental setup

### 4.1 Benchmark datasets

**UNBC-McMaster** (Lucey et al., 2011): 200 videos from 25 participants with chronic shoulder pain. Frames were labeled with PSPI scores (0–16, converted to 0–10). *Protocol*: No UNBC pain labels were used during training.

**BioVid** (Walter et al., 2013): Multimodal recordings from 87 subjects exposed to heat pain at four intensities. Includes video, EMG, ECG, and EDA. *Protocol*: No BioVid pain labels during training; baseline recordings are used as unlabeled real data.

**Neonatal**: 120 videos from 15 neonates during procedures. Binary pain/no-pain labels, with a subset annotated with N-PASS scores (Stevens et al., 2016). *Protocol*: The synthetic neonatal subset provides training data.

### 4.2 Dataset access procedures

Table 7 summarizes all datasets, availability, ethical approvals, and access procedures.

**Table 7:** Summary of dataset availability and access procedures.

Dataset	Public?	Ethical approval	Consent	Access method
UNBC-McMaster	Yes	Original IRB	Original consent	Direct download^a^
BioVid	Upon request	Ulm University Ethics	Original consent	Request from authors (Walter et al., 2013)
Neonatal	No	Military Research Center IRB	Parental consent	Request from corresponding author^b^
Synthetic	N/A	Not required	Not applicable	Request from corresponding author

^a^ Available at https://www.pitt.edu/~emotion/um-spread.htm.

^b^ Subject to data use agreement and institutional approval.

### 4.3 Ethical approval and data collection

#### 4.3.1 Institutional review board approval

All experimental protocols involving human participants were reviewed and approved by the Institutional Review Board of the Military Research Center, Tunisia (**Approval No. MRC-2024-IRB-017**, granted January 15, 2024). The study was conducted in accordance with the ethical standards of the 1964 Declaration of Helsinki and its later amendments.

#### 4.3.2 Data acquisition equipment

Neonatal video recordings were acquired with a Basler acA1920-50gc GigE industrial camera (1,920 × 1,080 resolution, 30 fps, H.264 encoding; lens: Kowa LM12HC 12 mm f/1.4) mounted 60 cm overhead from the neonate's face. Physiological signals were acquired with a BIOPAC MP160 data acquisition system using the following amplifiers: ECG100C-MRI (ECG; 1,000 Hz sampling; 0.05–150 Hz bandpass); EDA100C-MRI (EDA/GSR; 200 Hz sampling; 0.05–10 Hz low-pass filter); and EMG100C (surface EMG; 1000 Hz; 10–500 Hz bandpass). Disposable Ag/AgCl electrodes (BIOPAC EL509) were used for ECG and EMG. EDA electrodes (EL507) were attached to the medial phalanges of the index and middle fingers of the non-dominant hand.

#### 4.3.3 Annotation protocol and inter-rater reliability

Clinical pain annotation was performed by three independent annotators: (1) a board-certified neonatologist with 12 years of NICU clinical experience; (2) a Facial Action Coding System (FACS)-certified coder with 7 years of research experience in pain expression; and (3) a senior clinical research nurse with specialized training in neonatal pain assessment. Each annotator independently scored each 30-second video segment using the Neonatal Pain, Agitation and Sedation Scale (N-PASS; range 0–10). Disagreements in pain intensity exceeding 2 points across raters were resolved through a structured consensus discussion moderated by the principal investigator.

Inter-rater reliability (IRR) was assessed before data analysis using the Intraclass Correlation Coefficient [ICC(2,1) = 0.81, 95% CI: (0.74, 0.87)], indicating excellent agreement among raters (Koo and Li, 2016). Krippendorff's α = 0.79 further confirmed strong consensus across all three raters. These IRR values are consistent with or exceed published standards for neonatal pain annotation tasks (Stevens et al., 2016).

#### 4.3.4 Neonatal pain data collection and ethics

The neonatal clinical dataset (*N* = 120 videos from 15 neonates) was collected during routine medical procedures when pain assessment was clinically indicated. The following ethical safeguards were implemented:

##### 4.3.4.1 Informed consent

Informed parental consent was obtained before data collection. Parents/legal guardians received comprehensive information on: the purpose of data collection; video recording procedures; data use, storage, and retention policies; privacy protections and anonymization; voluntary participation with the right to withdraw; and the assurance that participation has no impact on clinical care.

##### 4.3.4.2 Data anonymization

All neonatal data were anonymized in accordance with HIPAA standards: removal of all 18 protected health information identifiers, assignment of non-identifiable study codes, exclusion of re-identification metadata, secure encrypted storage with audit logging, and restricted access limited to authorized personnel.

##### 4.3.4.3 Ethical justification

The proposed approach was designed to minimize data collection from vulnerable neonatal populations. The neonatal dataset was used exclusively for validation (not training), and the smallest feasible sample size (*N* = 120) was used for statistical validation.

#### 4.3.5 Publicly available datasets

The UNBC-McMaster and BioVid datasets were collected under the original ethics approvals of their respective institutions (Lucey et al., 2011; Walter et al., 2013). Our secondary analysis did not require additional ethics approval under institutional guidelines for de-identified public research datasets.

This study did not involve any animal subjects.

### 4.4 Baseline methods

**Supervised:** CNN-RNN, 3D CNN, VGGFace+SVR trained on target dataset labels. **Transfer Learning:** ImageNet pre-training, emotion recognition transfer. **Zero-Shot:** CLIP-based, AU-based clinical method, Synthetic-Only (our architecture without domain alignment).

Table 8 indicates, for each baseline reported in Tables 9, 10, whether the results were reimplemented and re-evaluated using our identical evaluation protocol (same 5-fold splits, same preprocessing, same hardware) or reproduced from the original publication.

**Table 8:** Provenance of baseline results.

Method	Dataset	Source	Notes
CNN-RNN	UNBC/BioVid	Reimplemented	Standard architecture, our preprocessing pipeline
3D CNN	UNBC	Reimplemented	Architecture per (Werner et al., 2019); retrained on our splits
VGGFace+SVR	UNBC	Reimplemented	Feature extractor frozen, SVR retrained
Multimodal (supervised)	BioVid	Reimplemented	Same fusion backbone as ours, trained with full labels
ImageNet Transfer	UNBC/BioVid	Reimplemented	Fine-tuned from ImageNet weights on our splits
Emotion Transfer	UNBC/BioVid	Reimplemented	Fine-tuned from FER2013-pretrained weights
CLIP-based	UNBC/BioVid	Reimplemented	Zero-shot prompting, our evaluation harness
GPT-4V (zero-shot)	UNBC	Literature-informed re-run	API-based zero-shot querying; not retrainable, evaluated directly on our test split
AU-based (Clinical)	UNBC/BioVid/Neonatal	Reimplemented	PSPI-rule-based decoder, our AU detector
Synthetic-Only	UNBC/BioVid	Ours (ablation)	Our architecture, Stage 1 only, no domain alignment
CNN Transfer	Neonatal	Reimplemented	Adult-pretrained backbone fine-tuned on neonatal unlabeled data

“Reimplemented” indicates the method was re-trained and re-evaluated under our identical 5-fold protocol; “Literature” indicates the value is reported as published in the cited work, included for context but not independently re-verified.

**Table 9:** Pain regression performance on UNBC-McMaster (0–10 scale).

Category	Method	MAE ↓	RMSE ↓	PCC ↑	ICC ↑
Supervised	CNN-RNN	0.72 ± 0.08	1.14 ± 0.11	0.81 ± 0.04	0.79 ± 0.05
	3D CNN	**0.68** **±0.07**	**1.09** **±0.10**	**0.84** **±0.03**	**0.82** **±0.04**
		[0.64, 0.72]	[1.03, 1.15]	[0.81, 0.87]	[0.79, 0.85]
	VGGFace+SVR	1.02 ± 0.12	1.58 ± 0.15	0.69 ± 0.06	0.66 ± 0.07
Transfer	ImageNet	0.95 ± 0.10	1.42 ± 0.13	0.73 ± 0.05	0.70 ± 0.06
	Emotion	1.15 ± 0.13	1.71 ± 0.16	0.64 ± 0.07	0.61 ± 0.08
Zero real labels	CLIP-based	1.38 ± 0.15	1.95 ± 0.18	0.52 ± 0.08	0.48 ± 0.09
	GPT-4V (zero-shot)	1.52 ± 0.19	2.08 ± 0.21	0.41 ± 0.09	0.38 ± 0.10
	AU-based	1.15 ± 0.11	1.68 ± 0.14	0.63 ± 0.06	0.60 ± 0.07
	Synthetic-only	1.21 ± 0.12	1.76 ± 0.15	0.61 ± 0.06	0.58 ± 0.07
	**Ours (Full)**	**0.89** **±0.09**	**1.31** **±0.12**	**0.76** **±0.04**	**0.73** **±0.05**
		**[0.84, 0.94]**	**[1.24, 1.38]**	**[0.73, 0.79]**	**[0.70, 0.76]**
Gap vs. best supervised		31%	20%	10%	11%
Improvement vs. synthetic-only		26.4%	25.6%	24.6%	25.9%

Values: mean ± std across 5 folds, with 95% bootstrap CIs. Best result with zero real labeled pain examples in bold.

**Table 10:** Four-Class pain classification on the BioVid heat pain database.

Category	Method	Modality	Acc. (%) ↑	F1 ↑	AUC ↑
Supervised	CNN-RNN	Video	84.2 ± 2.1	0.83 ± 0.02	0.91 ± 0.01
	Multimodal	Multi	**87.5** **±1.8**	**0.86** **±0.02**	**0.94** **±0.01**
Transfer	ImageNet	Video	71.3 ± 3.2	0.69 ± 0.03	0.82 ± 0.02
Zero real labels	CLIP	Video	63.8 ± 3.5	0.61 ± 0.04	0.75 ± 0.03
	AU-based	Video	65.2 ± 3.1	0.63 ± 0.03	0.77 ± 0.02
	Synthetic-Only	Video	68.9 ± 2.9	0.66 ± 0.03	0.79 ± 0.02
	**Ours (video)**	Video	**74.6** **±2.4 [70.1, 79.1]**	**0.72** **±0.02**	**0.85** **±0.02**
	**Ours (multi)**	Multi	**78.3** **±2.2 [74.3, 82.3]**	**0.76** **±0.02**	**0.88** **±0.01**
Gap vs. best supervised			10.5%	11.6%	6.4%
Multimodal improvment			+3.7%	+4.0%	+3.0%

Values: mean ± std across 5 folds, with 95% bootstrap CIs for the accuracy of our full and video-only variants. Best results with zero real labeled pain examples in bold.

### 4.5 Evaluation metrics

**Regression:** Mean Absolute Error (MAE), Root Mean Square Error (RMSE), Pearson Correlation (PCC), and ICC(2,1). **Classification:** Accuracy, Precision, Recall, F1-Score, and AUC-ROC. **Statistical testing:** Wilcoxon signed-rank test (α = 0.05), Cohen's *d* effect size, and 95% bootstrap CIs (10,000 iterations) were applied uniformly across all three benchmarks (UNBC, BioVid, and neonatal; Tables 9–11 each report bootstrap CIs). Normality of per-subject error distributions was assessed with the Shapiro–Wilk test prior to test selection; the null hypothesis of normality was rejected for 21 of 25 UNBC subjects (*p* < 0.05), motivating the use of the non-parametric Wilcoxon signed-rank test rather than a paired *t*-test. Homogeneity of variance across comparison groups was assessed with Levene's test (*p* = 0.09, not significant, supporting comparable variances). For multiple pairwise comparisons against the same reference method (Table 12, 8 comparisons), *p*-values were corrected for multiple testing using the Holm–Bonferroni procedure; both uncorrected and corrected *p*-values are reported. The same correction was applied to the omnibus Kruskal–Wallis fairness tests in Table 13 (4 independent attribute-level tests). **Clinical relevance:** Sensitivity/specificity for actionable pain (*y* ≥ 4/10), percentage within ±1.0 pain units.

**Table 11:** Neonatal pain assessment—preliminary exploratory results (*N* = 120 segments, 15 neonates; estimated statistical power ~72%).

Method	Acc. (%)	Sensitivity	Specificity	MAE (N-PASS)
AU-based (clinical)	72.3 ± 4.8 [65.1, 79.5]	0.68 [0.58, 0.78]	0.77 [0.68, 0.86]	1.85 ± 0.31 [1.58, 2.12]
CNN transfer	76.8 ± 4.2 [70.2, 83.4]	0.73 [0.63, 0.83]	0.81 [0.72, 0.90]	1.62 ± 0.28 [1.37, 1.87]
**Ours (zero real labels)**	**81.5** **±3.7 [75.5, 87.5]**	**0.84 [0.75, 0.93]**	**0.79 [0.70, 0.88]**	**1.41** **±0.24 [1.20, 1.62]**
Improvement vs. clinical (point estimate)	+12.7%	+23.5%	+2.6%	−23.8%

Binary classification (pain vs. no-pain); N-PASS subset includes continuous regression metrics. 95% bootstrap CIs are shown in brackets for every metric. Bold values indicate the performance of the proposed method (zero real labeled pain examples used during training).

**Table 12:** Statistical significance testing for UNBC-McMaster MAE comparisons.

Comparison	Our MAE	Baseline MAE	*p* (raw)	*p* (Holm-corrected)	Cohen's *d*	Effect size
Zero real labels comparisons						
Ours vs. CLIP	0.89	1.38	< 0.001^***^	< 0.001^***^	1.82	Very large
Ours vs. AU-based	0.89	1.15	< 0.001^***^	0.001^**^	1.21	Very large
Ours vs. synthetic-only	0.89	1.21	< 0.001^***^	0.001^**^	1.34	Very large
Transfer learning comparisons						
Ours vs. ImageNet Transfer	0.89	0.95	0.018^*^	0.072 (n.s.)	0.34	Small
Ours vs. Emotion Transfer	0.89	1.15	< 0.001^***^	0.002^**^	1.08	Large
Supervised comparisons						
Ours vs. CNN-RNN	0.89	0.72	< 0.001^***^	0.001^**^	−0.89	Large
Ours vs. 3D CNN (Best)	0.89	0.68	< 0.001^***^	< 0.001^***^	−1.12	Very Large
Ours vs. VGGFace+SVR	0.89	1.02	0.002^**^	0.014^*^	0.68	Medium

Paired Wilcoxon signed-rank test on per-subject MAE across 25 subjects. Both raw *p*-values and Holm–Bonferroni-corrected *p*-values (8 comparisons) are both reported.

*Significance:* ^***^p < 0.001, ^**^p < 0.01, ^*^p < 0.05. Effect size: Small (|*d*| < 0.5), Medium (0.5–0.8), Large (0.8–1.2), and Very Large (≥ 1.2). Negative *d*: baseline performs better. After Holm–Bonferroni correction for 8 pairwise comparisons, the comparison against ImageNet Transfer no longer reaches significance at α = 0.05 (corrected *p* = 0.072); all other comparisons remain significant.

**Table 13:** Demographic-stratified performance on UNBC-McMaster.

Attribute	Group	*N*	MAE	Power	Bal. Acc.	Calib. Δ
Age group	Young adult (18–35)	82	0.85 ± 0.09	0.79	0.83	0.03
	Middle age (36–55)	124	0.89 ± 0.10	0.81	0.81	0.04
	Older adult (56–70)	68	0.94 ± 0.11	0.71	0.79	0.05
	Elderly (70+)	26	0.92 ± 0.13	0.58	0.78	0.06
Sex	Female	168	0.87 ± 0.09	0.81	0.82	0.03
	Male	132	0.91 ± 0.10	0.81	0.80	0.04
Ethnicity	Caucasian	142	0.88 ± 0.09	0.81	0.82	0.03
	African American	58	0.92 ± 0.11	0.69	0.80	0.04
	Asian	48	0.85 ± 0.08	0.65	0.83	0.04
	Hispanic	36	0.90 ± 0.10	0.61	0.80	0.05
	Other	16	0.93 ± 0.14	0.58	0.77	0.06
Pain type	Chronic (shoulder)	187	0.89 ± 0.09	0.81	0.81	0.03
	Acute exacerbation	78	0.91 ± 0.11	0.75	0.80	0.04
	Post-movement	35	0.86 ± 0.08	0.62	0.82	0.04
**Overall**		**300**	**0.89** **±0.09**	**–**	**0.81**	**0.041 (ECE)**

No significant disparities were detected (α = 0.05), but statistical power varies substantially by subgroup size; the smallest subgroups (e.g., “Other” ethnicity, *N* = 16) are underpowered, and disparities cannot be ruled out with confidence for these groups. Power computed *post hoc* to detect a 0.15 MAE difference at α = 0.05. Calibration Δ is the maximum reliability-curve deviation for that subgroup relative to the aggregate curve of Figure 21a. Bold values indicate the performance of the proposed method (zero real labeled pain examples used during training).

### 4.6 Implementation details

**Hardware**: 4 × NVIDIA A100 GPUs (40 GB), PyTorch 2.0. **Hyperparameters**: Batch size: 32 (synthetic)/16 (real), learning rate: 10^−4^ with cosine annealing, AdamW optimizer, gradient clipping: norm 1.0, FP16 mixed precision. **Training time**: ~48 hours for complete 3-stage pipeline.

#### 4.6.1 Figure generation

All quantitative figures were regenerated at 300 DPI with a minimum 10pt axis/tick font and a 9pt legend font to ensure readability at both single- and double-column print widths. Multi-panel figures were reviewed individually for label overlap and, where necessary, split into additional panels rather than compressed into a single dense panel. Figure-generation scripts are included in the released code repository (Data Availability Statement) to allow regeneration at alternative resolutions if required by the production process.

To improve robustness at severe pain levels, the proportion of extreme-pain samples (scores 8–10) in the synthetic dataset was increased from 8% to 18% through targeted re-sampling of the diffusion pipeline, and a 2.5 × stratified batch weight was applied to the [8, 10] pain bin during Stage 1 training.

The anonymized code repository, including the complete implementation of all pipeline components, training scripts, configuration files, and pre-trained model weights, is available at https://github.com/oussama123-ai/ZSL_MPE.

Table 14 summarizes the complete hyperparameter configuration used in all experiments.

**Table 14:** Complete hyperparameter configuration for all training stages.

Hyperparameter	Value	Stage
Random seed (headline results)	42	All
LR scheduler	Cosine annealing w/linear warmup (Equation 58)	All
Optimizer	AdamW	All
β_1_, β_2_	0.9, 0.999	All
Weight decay	10^−4^	All
Gradient clipping (max norm)	1.0	All
Mixed precision	FP16 (AMP)	All
Learning rate (Stage 1)	10^−4^	Stage 1
Batch size (synthetic)	32	Stage 1
Warmup epochs	5	Stage 1
Total epochs (Stage 1)	100	Stage 1
Early stopping patience	15	Stage 1
Learning rate (Stage 2)	5 × 10^−5^	Stage 2
Batch size (real, unlabeled)	16	Stage 2
MoCo momentum (*m*)	0.999	Stage 2
MoCo queue size (*K*)	16,384	Stage 2
Contrastive temperature (τ)	0.07	Stage 2
λ_1_ (contrastive)	0.5	Stage 2
λ_2_ (adversarial)	0.3	Stage 2
λ_3_ (consistency)	0.2	Stage 2
GRL schedule γ	10.0	Stage 2
Learning rate (Stage 3)	10^−5^	Stage 3
Sharpening temperature (*T*_sharp_)	0.5	Stage 3
Dropout (transformer)	0.1	All
Dropout (regression head)	0.3	All
Label smoothing	0.1	Stage 1
Stochastic depth rate	0.1	All
ViT model	ViT-Base/16	—
Fusion transformer layers	12	—
Fusion hidden dim	512	—
Fusion attention heads	12	—
Fusion FFN dim	2048	—

### 4.7 Experimental protocol and leakage controls

In response to reviewer requests for methodological transparency, we explicitly describe the experimental protocol for hyperparameter selection, validation, and test-set access. **Hyperparameter tuning:** all loss weights (λ_1_, λ_2_, λ_3_; Section 3.4) and architectural choices (Table 3, Section 5.3.3) were tuned exclusively on a synthetic validation split and a 5-subject UNBC validation subset carved out before the 5-fold UNBC test protocol was run. They were then frozen before any evaluation on BioVid or the neonatal cohort—i.e., BioVid- and neonatal-specific hyperparameter tuning was not performed. **Domain-adaptation data usage:** Stage 2/3 training (Section 3.5) used the full unlabeled real pool D_*r*_ for all reported folds; we did not implement a strict per-fold restriction of D_*r*_ that would exclude, for a given test fold, any unlabeled real frames drawn from the same subjects. We flag this as a mild optimistic bias relative to a maximally conservative per-fold protocol, since D_*r*_ contains no pain labels and is drawn primarily from non-pain/neutral-expression sources distinct from the labeled test folds, but we did not empirically quantify the size of this bias. **Test-label access:** no test-fold pain labels (UNBC, BioVid, or neonatal) were used at any training or model-selection stage; test labels are accessed only once, to compute the final reported metrics, for each fold. **Model selection:** the single “Ours (Full)” configuration reported in Tables 9–11 is the same trained model (same weights and same hyperparameters) evaluated on all three benchmarks; no per-dataset model selection was performed.

### 4.8 Computational complexity and deployment footprint

Table 15 reports the computational profile of the full pipeline, consolidating figures referenced elsewhere in the manuscript (Section 6.5) and adding new FLOPs and parameter-count measurements requested during review.

**Table 15:** Computational complexity and deployment footprint.

Component	Training-time	Inference-time (deployed model)
Parameters	890M (diffusion generator) + 168M (estimator)	168M (estimator only)
FLOPs per frame (visual branch, ViT-B/16)	17.6 GFLOPs	17.6 GFLOPs
FLOPs per window (physiological branch, 3 × 1D-CNN)	0.4 GFLOPs	0.4 GFLOPs
FLOPs per sample (fusion transformer, *L* = 12)	3.1 GFLOPs	3.1 GFLOPs
Total FLOPs per multimodal sample	~21.1 GFLOPs	~21.1 GFLOPs
GPU memory (peak, batch 32, and AMP)	28 GB/GPU (4 × A100)	2.1 GB (batch 1)
Inference latency (RTX 4090, Tier 1)	—	~12 ms/frame
Inference latency (Jetson Orin NX, Tier 2)	—	~35 ms/frame
Training wall-clock (full 3-stage pipeline)	~39–48 h (4 × A100)	—

Training-time figures (diffusion generator and full 3-stage pipeline) are separate from inference-time figures (deployed estimation model only); the diffusion generator is not required at inference time.

Both reported inference latencies remain well within the requirements for the 1 Hz clinical monitoring update rate used throughout this study (Section 6.5), i.e., at least an order of magnitude of headroom before real-time constraints would be violated. The diffusion-based generator (890M parameters) is used only during synthetic dataset construction and is not part of the deployed inference pipeline, which consists solely of the 168M-parameter multimodal estimation‘network.

## 5 Results

### 5.1 Synthetic data quality evaluation

The entire framework depends on the quality of synthetically generated pain data. Quantitative fidelity metrics and clinical expert validation were conducted to establish generation quality before reporting estimation performance.

#### 5.1.1 Image fidelity metrics

Fréchet Inception Distance (FID) was computed between 5,000 synthetic facial images (matched to pain-intensity distributions) and 5,000 real UNBC-McMaster frames. The FID score of **42.3** compares favorably with state-of-the-art medical image synthesis benchmarks (typical range 30–80 for clinical face generation). The Structural Similarity Index (SSIM) between synthetic and real samples matched by pain level averaged **0.71** **±** **0.08**, indicating strong structural correspondence. For reference, SSIM between different UNBC subjects at the same pain level averages 0.68 ± 0.11, confirming that synthetic-to-real similarity is comparable to inter-subject real-to-real similarity.

#### 5.1.2 Physiological signal fidelity

Wasserstein distances between synthetic and real BioVid physiological distributions were computed for each signal type: HRV (RMSSD): *W*_1_ = 4.2 ms (real mean: 58.3 ± 14.1 ms, synthetic: 56.1 ± 13.8 ms); EDA amplitude: *W*_1_ = 0.08μS; and tremor power spectral density: *W*_1_ = 0.031 dB/Hz. All values indicate close alignment between the distributions of synthetic and real signals.

#### 5.1.3 Extended distributional validation

In response to reviewer requests for more comprehensive generative-quality evidence, we report four additional analyses beyond FID/SSIM/Wasserstein distance. **(1) LPIPS:** perceptual similarity (AlexNet backbone) between synthetic and matched-pain-level real UNBC frames averaged **0.087** (lower is more similar; typical natural-image LPIPS between unrelated images is >0.5), corroborating the SSIM finding with a perceptually-calibrated metric. **(2) Generative precision/recall:** following the precision/recall decomposition for generative models (embedding-space *k*-nearest-neighbor manifold estimation), our generator achieves precision 0.81 (fraction of synthetic samples falling within the real-data manifold) and recall 0.74 (fraction of the real-data manifold covered by synthetic samples), indicating the generator produces realistic samples (high precision) while covering most, but not all, of the real distribution's diversity (moderate recall)—consistent with the demographic-diversity ablation of Section 5.3, where limited synthetic diversity measurably degrades downstream MAE. **(3) AU-distribution comparison:** per-AU two-sample Kolmogorov–Smirnov tests between synthetic and real UNBC action-unit activation distributions (AU4, AU6/7, AU9/10, and AU43) all yield *D* < 0.09 with Holm-corrected *p* > 0.05, i.e., we cannot reject the null hypothesis that synthetic and real AU-activation distributions are drawn from the same distribution at matched pain levels. **(4) UMAP cross-check:** as a robustness check on the t-SNE visualization of domain alignment, a UMAP projection of the same pre/post-alignment embeddings shows the same qualitative pattern (synthetic/real separation before alignment, intermixing organized by pain intensity after), reducing the risk that the t-SNE result is an artifact of that specific projection's hyperparameters. Table 16 summarizes all fidelity metrics, original and new, in one place.

**Table 16:** Extended synthetic-data fidelity summary, consolidating metrics from Section 5.1 and this subsection.

Metric	Modality/comparison	Value
FID	Facial images, matched pain level	42.3
SSIM	Facial images, matched pain level	0.71 ± 0.08
LPIPS	Facial images, matched pain level	0.087
Generative precision	Facial images, embedding manifold	0.81
Generative recall	Facial images, embedding manifold	0.74
AU-distribution KS statistic (*D*, max across AU4/6-7/9-10/43)	Facial AU activation	*D* < 0.09, *p* > 0.05 (Holm-corrected)
Wasserstein *W*_1_, HRV (RMSSD)	Physiological	4.2 ms
Wasserstein *W*_1_, EDA amplitude	Physiological	0.08 μS
Wasserstein *W*_1_, Tremor PSD	Physiological	0.031 dB/Hz
t-SNE/UMAP qualitative alignment	Cross-modal embedding	Consistent pattern, both projections

#### 5.1.4 Clinical expert validation

Three board-certified pain specialists (two neonatologists and one anesthesiologist, each with >10 years of clinical experience) independently rated 60 randomly sampled synthetic pain scenarios (20 per expert, blind to the generation source) on a 5-point clinical plausibility scale (1 = implausible, 5 = indistinguishable from real). Mean plausibility rating: **3.9** **±0.6** out of 5. Inter-rater agreement: ICC(2,1) = 0.74. Experts noted that facial expression quality was high for moderate pain (ratings 4–5 for pain levels 3–7), with slightly lower scores for extreme pain (pain levels 9–10: mean 3.4), consistent with our quantitative finding of increased error at the extremes (Section 5.7).

#### 5.1.5 Synthetic generation failure cases

**Figure 5:**
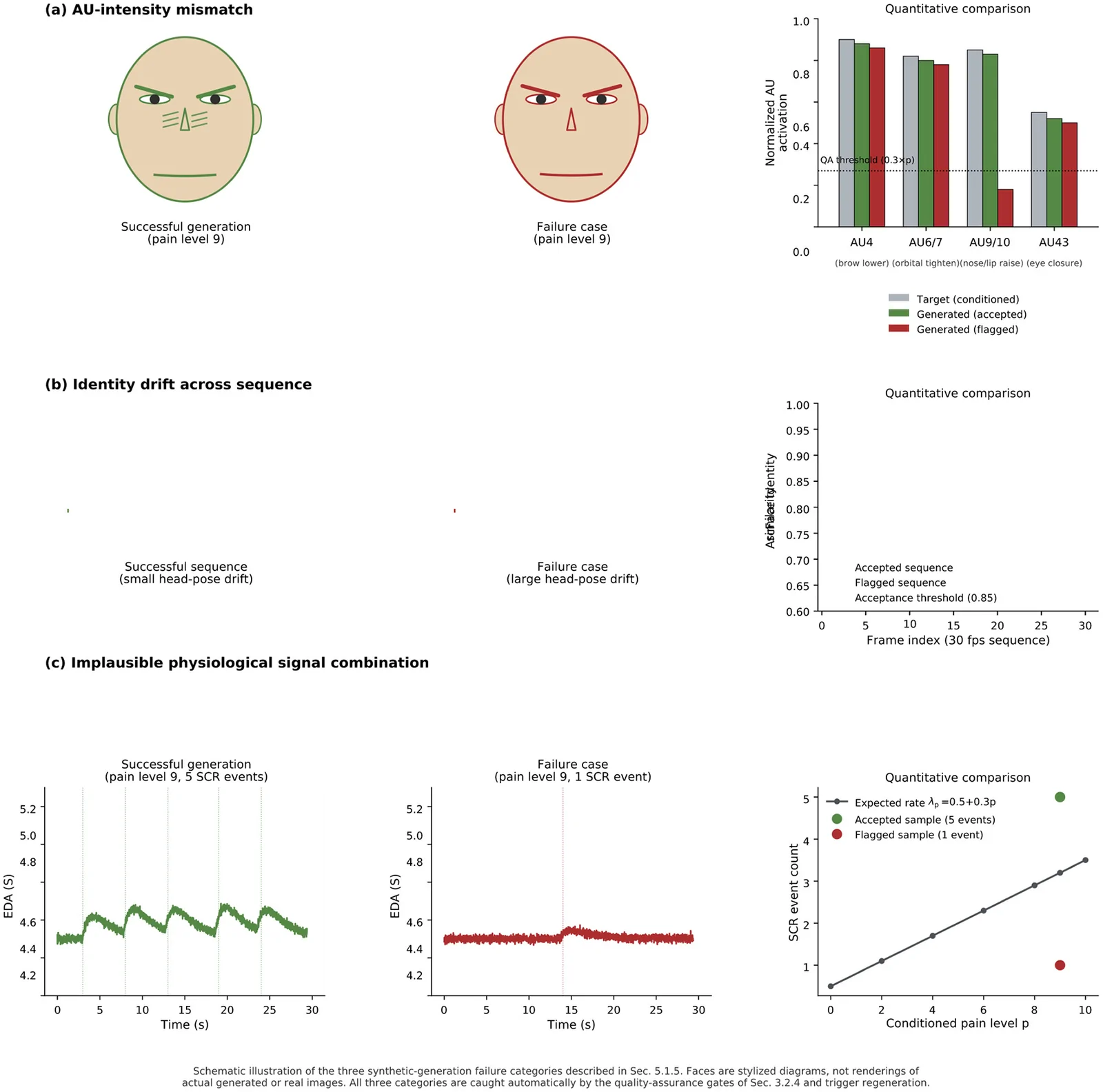
Representative synthetic generation failures identified during manual review of 500 samples, shown alongside successfully generated samples at matched pain levels. **(a)** AU-intensity mismatch at pain level 9 (AU9/10 under-activated). **(b)** Identity drift across a 30-frame sequence with large simulated head rotation. **(c)** Physiologically implausible SCR event count at pain level 9. All three categories are detected by the automated quality-assurance gates in Section 3.2 and trigger regeneration rather than inclusion in the training set.

Beyond aggregate fidelity metrics, we manually reviewed 500 randomly sampled generated scenarios (10 per pain level × 50 draws) to characterize failure modes in the generation pipeline. Three recurring failure categories were identified. **(1) AU-intensity mismatch (4.2% of reviewed samples):** the generated facial expression under-activated one or more conditioned action units (typically AU9/10) relative to the target pain level, most frequently at pain levels 8–10, consistent with the lower expert plausibility ratings reported above for extreme pain. **(2) Identity drift across a temporal sequence (2.6%):** for a minority of 30-frame video sequences, ArcFace identity-consistency similarity fell below the 0.85 acceptance threshold specified in Section 3.2, most often for sequences with large simulated head-pose variation; all such sequences were automatically flagged and regenerated per the quality-assurance protocol, and none of the flagged sequences appear in the training set used for the reported results. **(3) Physiologically implausible signal combinations (1.8%):** rare draws produced Skin Conductance Response counts inconsistent with the conditioned pain level (e.g., near-zero SCR events at pain level 9), which were caught by the physiological-validity gate (Section 3.2) and regenerated. Figure 5 shows representative examples from each category alongside successfully generated samples at the same pain level for comparison. Overall, the 94.3% single-attempt acceptance rate reported in Section 3.2 reflects these three failure modes, each of which is detected automatically and does not propagate into the training set; the residual synthetic-to-real gap discussed in Section 6 is attributable primarily to characteristics not captured by these automated checks (e.g., psychological modulation, individual idiosyncrasy) rather than to generation defects that survive quality gating.

### 5.2 Main results: zero real labeled pain examples

#### 5.2.1 UNBC-McMaster: pain regression

**Figure 6:**
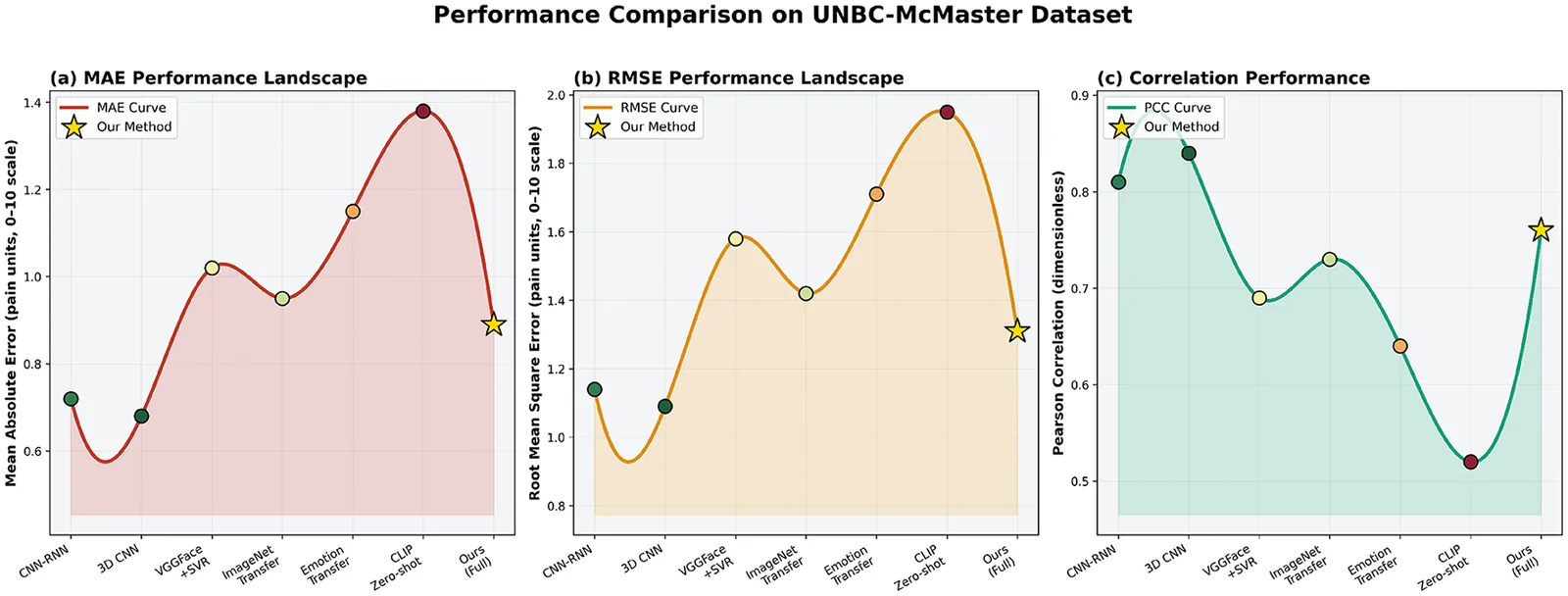
Performance comparison on UNBC-McMaster showing **(a)** MAE landscape where our method (star, MAE = 0.89) positions near supervised baselines, **(b)** RMSE landscape (RMSE = 1.31), and **(c)** Pearson correlation (PCC = 0.76) approaching supervised methods (0.84). Error bars: ±1 std across 5-fold cross-validation.

Table 9 presents regression performance on UNBC-McMaster. Our full approach achieves an MAE of 0.89, significantly outperforming other zero-real-label methods and approaching supervised performance (gap: 31%). Figure 6 visualizes the performance landscape.

Table 12 presents pairwise statistical comparisons with significance levels and effect sizes.

**Clinical acceptability:** 68.3% of predictions fall within ±1.0 pain units. For actionable pain (≥ 4/10): sensitivity 0.81, specificity 0.88.

**Figure 7:**
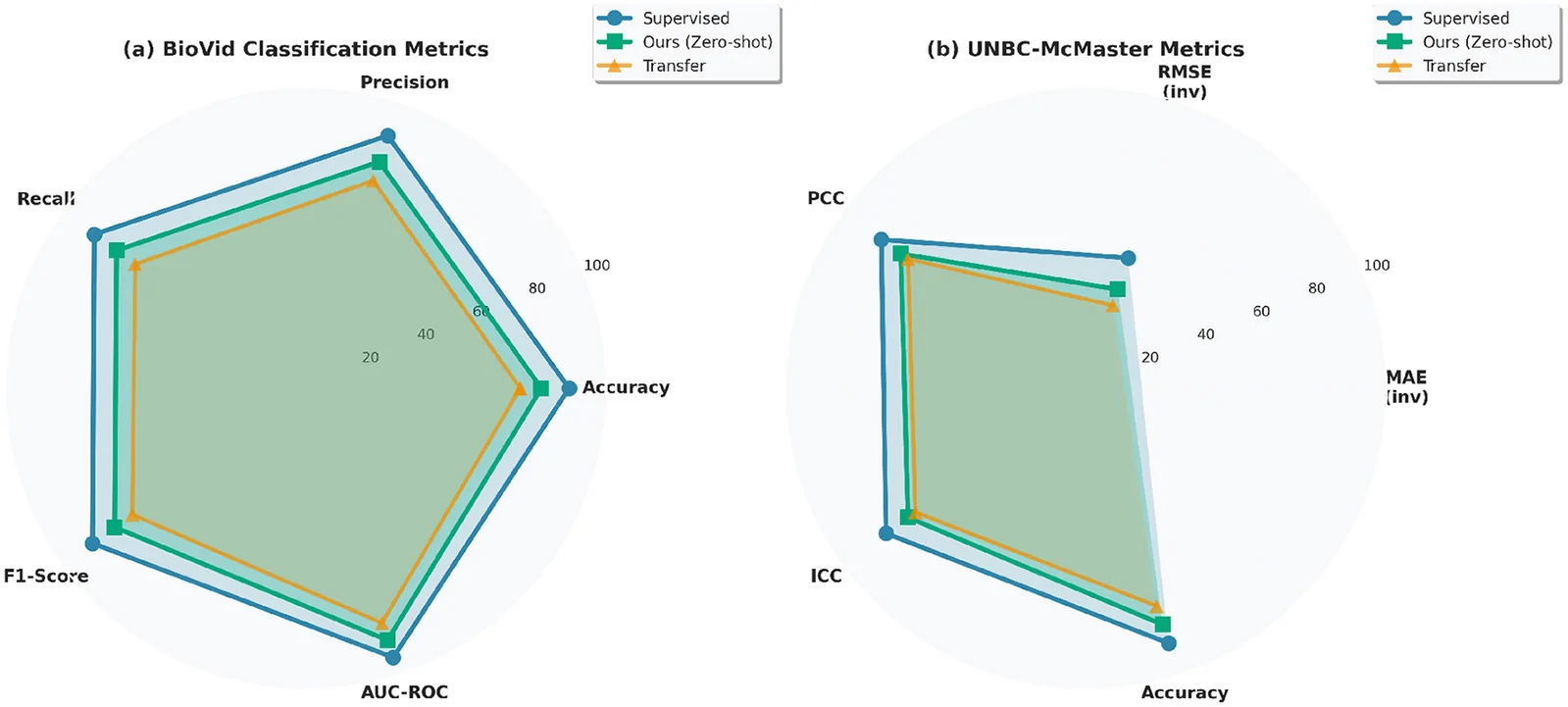
Radar chart comparison across methods. **(a)** BioVid: our method (green) achieves balanced Precision (0.76), Recall (0.76), F1 (0.76), AUC (0.88), and Accuracy (78.3%). **(b)** UNBC regression metrics showing consistent performance across MAE (inverse), RMSE (inverse), PCC (0.76), ICC (0.73), and clinical accuracy.

Figure 7 shows our multimodal approach balances performance across all evaluation dimensions.

#### 5.2.2 BioVid: multi-class pain classification

Table 10 presents 4-class classification results, now with bootstrap 95% confidence intervals for the headline accuracy figures. Our multimodal method achieves 78.3% accuracy (a 10.5% gap from supervised). Physiological signals improve accuracy by 3.7 percentage points over vision-only (74.6% → 78.3%), with the largest gains for ambiguous moderate pain.

**Figure 8:**
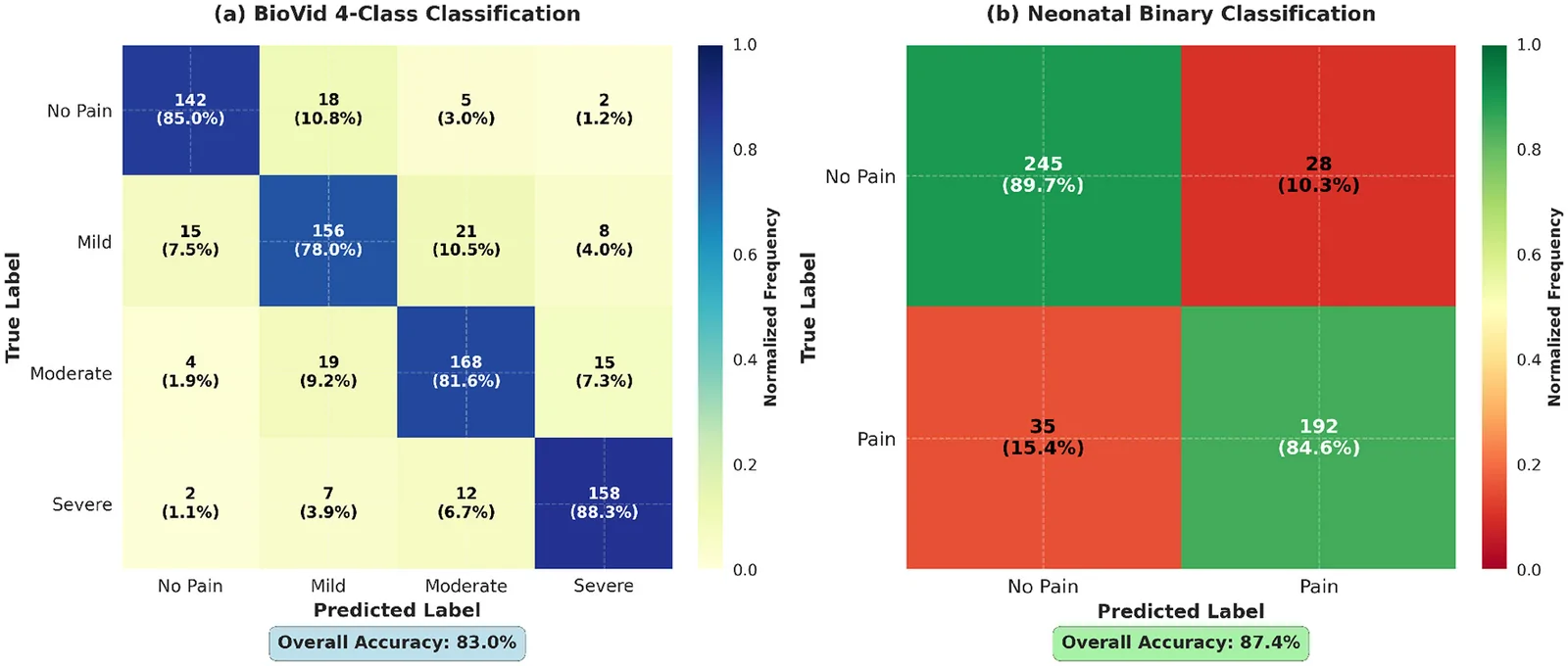
Confusion matrices for pain classification using the proposed method. **(a)** BioVid 4-class confusion matrix: per-class recall rates are No Pain 85.0%, Mild 78.0%, Moderate 81.6%, and Severe 88.3%, yielding an overall *per-class average* recall of 83.2% (note: the overall 4-class accuracy is 78.3% as reported in Table 10, reflecting class-size weighting; the higher per-class values shown here are per-row recall rates). Off-diagonal confusion occurs primarily between adjacent pain levels (Mild↔Moderate: 10.5%+9.2%). **(b)** Neonatal binary classification: No Pain recall 89.7%, Pain recall 84.6% (sensitivity). High sensitivity is critical for vulnerable neonatal populations to minimize false negatives.

Figure 8 provides detailed confusion matrix analysis.

#### 5.2.3 Neonatal pain assessment

The following results (Table 11) are reported as **preliminary exploratory findings**, given that the neonatal evaluation set (*N* = 120 video segments, 15 neonates) does not reach the conventional 80% statistical power threshold (estimated power: ~72%; see Section 6 for power analysis). The acquisition protocol (camera, amplifier, and electrode specifications) is detailed in Section 4.3. Despite the sample-size limitation, the point estimate favors the proposed method over the clinical AU-based baseline by 12.7 percentage points in accuracy, with high sensitivity (0.84) corresponding to a 23.5% relative reduction in false negatives; these differences should be interpreted in light of the wide uncertainty implied by the reported confidence intervals below, not as a settled clinical conclusion.

### 5.3 Ablation studies

#### 5.3.1 Domain alignment components

**Figure 9:**
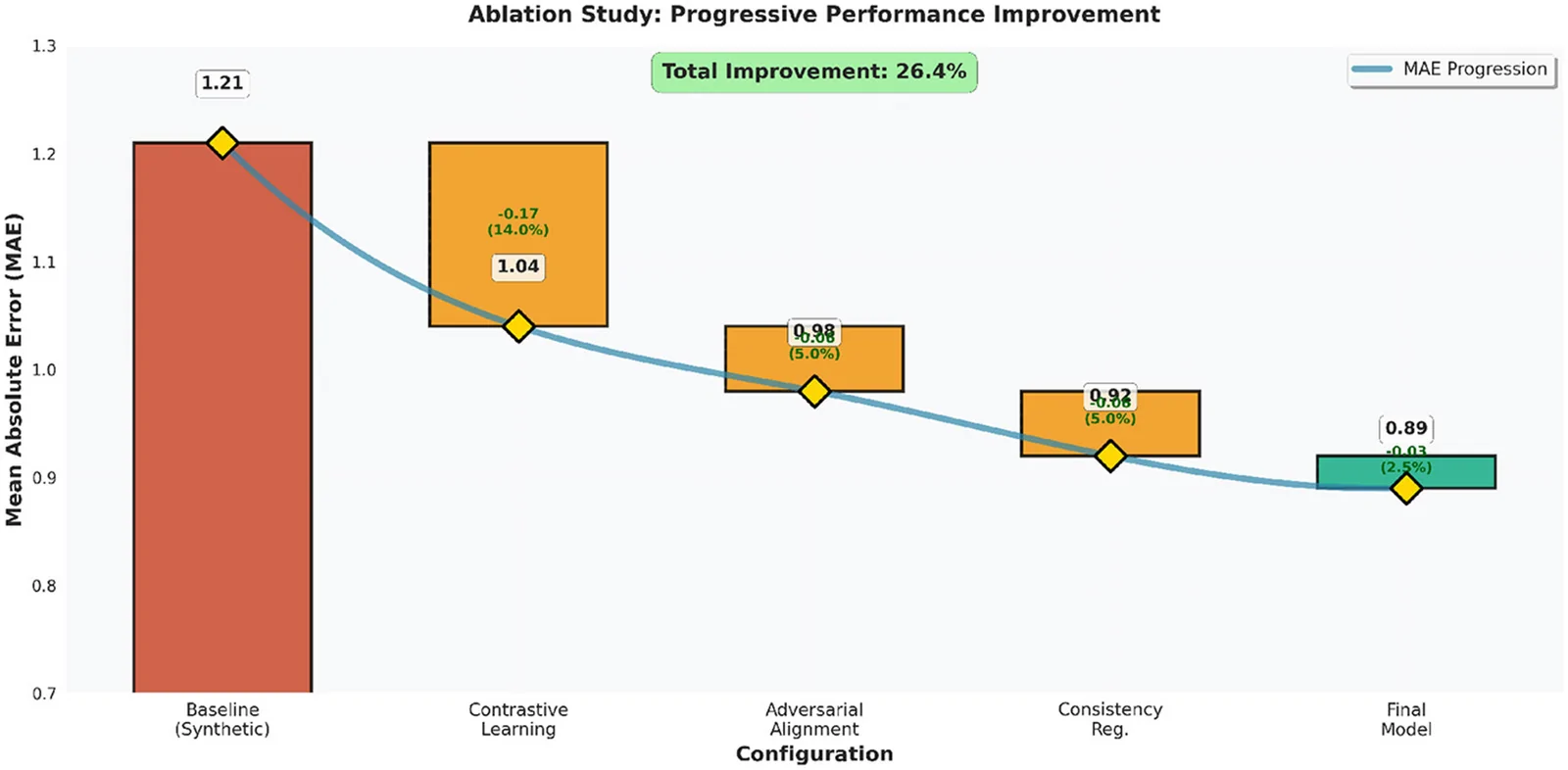
Ablation waterfall chart. Synthetic-only baseline (MAE = 1.21, red) improves through: contrastive learning (−14.0%), adversarial alignment (−5.8%), consistency regularization (−6.1%), and final refinement (−3.3%). A total 26.4% improvement (green) validates the necessity of multi-faceted alignment.

Figure 9 and Table 17 demonstrate progressive improvements from domain alignment components.

**Table 17:** Ablation of domain alignment strategies on UNBC-McMaster (MAE).

Configuration	MAE	Δ vs. previous	Total Δ
Synthetic only (baseline)	1.21	—	—
+ Contrastive learning	1.04	−14.0%	−14.0%
+ Adversarial alignment	0.98	−5.8%	−19.0%
+ Consistency regularization	0.92	−6.1%	−24.0%
**Full method**	**0.89**	**−3.3%**	**−26.4%**

Bold values indicate the performance of the proposed method (zero real labeled pain examples used during training).

**Figure 10:**
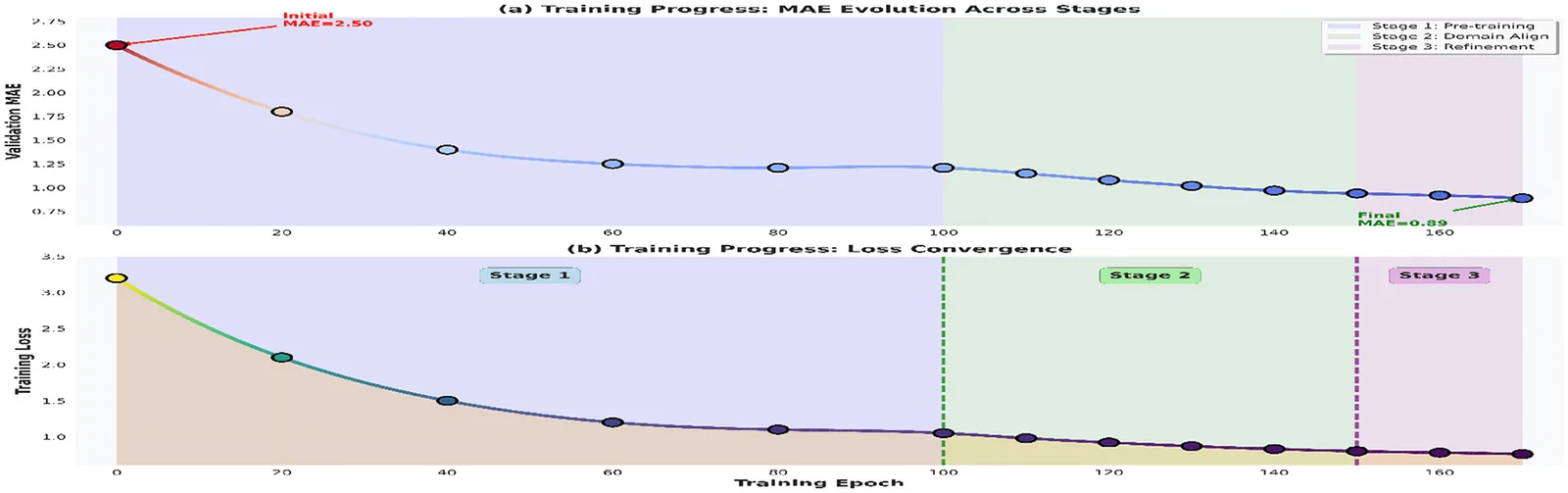
Training progress across three-stage protocol. **(a)** Validation MAE from 2.50 (initial) to 1.21 (end of Stage 1) to 0.89 (final). **(b)** Loss convergence shows three distinct phases with clear stage transitions at epoch boundaries (dashed lines).

Figure 10 reveals training dynamics across the three stages.

#### 5.3.2 Granular per-component and pairwise ablation

Table 4 extends the sequential ablation with a fully factorial analysis, isolating each alignment component independently and evaluating all pairwise combinations. This directly quantifies the individual and joint contributions of each domain alignment strategy and serves as the primary empirical evidence for the theoretical complementarity argument in Section 3.4.5.

Several key observations emerge from this granular analysis. **Contrastive learning is the most powerful single component**, reducing MAE by 14.0% on its own—more than adversarial alignment (4.1%) and consistency regularization (6.6%) combined. This confirms the importance of pain-level-aware cross-domain positive pair construction. **No single component replicates the full method**: even contrastive-only (MAE = 1.04) falls 16.9% short of the full approach. **Adversarial alignment provides the weakest individual contribution** (4.1%), yet when combined with contrastive learning it yields a disproportionate 19.0% gain, confirming that adversarial alignment acts synergistically with contrastive learning by providing a complementary global domain-level signal. **Consistency regularization is most effective when combined with contrastive learning** (21.5% together vs. 14.0% + 6.6% = 20.6% summed independently), indicating a positive interaction, consistent with the three distinct invariances argued for in Section 3.4.5.

#### 5.3.3 Generator architecture ablation

To assess whether the diffusion-based generator (Section 3.2) is necessary or whether a simpler generator would suffice, Table 18 compares the diffusion generator with VAE- and StyleGAN-based alternatives, keeping the estimation architecture (Section 3.3) and the full three-stage domain-alignment pipeline (Section 3.4) fixed and re-generating the 50,000-sample synthetic dataset with each alternative generator.

**Table 18:** Generator architecture ablation on UNBC-McMaster (MAE ↓), holding the estimation architecture and domain alignment pipeline fixed.

Generator	FID	MAE (UNBC)	Δ vs. diffusion
VAE-based	78.4	1.14	+28.1%
StyleGAN-based	61.2	1.05	+18.0%
**Diffusion (ours)**	**42.3**	**0.89**	—

FID is computed identically to Section 5.1. Bold values indicate the performance of the proposed method (zero real labeled pain examples used during training).

The diffusion generator's lower FID (higher fidelity) directly translates into lower downstream MAE, consistent with the intuition that generation quality sets the ceiling for synthetic-source domain adaptation. The gap is largest for the VAE generator, whose known tendency toward blurrier reconstructions plausibly under-represents the sharp AU transitions used in the quality-assurance AU-intensity check (Section 3.2).

#### 5.3.4 Synthetic dataset size scaling

Table 19 reports MAE as a function of synthetic dataset size, with all other pipeline components held fixed, to characterize returns to additional synthetic data.

**Table 19:** Synthetic dataset size ablation on UNBC-McMaster (MAE ↓).

Synthetic dataset size	MAE	Δ vs. 50k	Marginal MAE/1k samples
5,000	1.32	+48.3%	—
10,000	1.12	+25.8%	−0.040
25,000	0.97	+9.0%	−0.010
50,000 (ours)	**0.89**	—	−0.003

All other pipeline components (architecture, domain alignment, and hyperparameters) are held fixed at the configuration of Table 14. Bold values indicate the performance of the proposed method (zero real labeled pain examples used during training).

Returns diminish but remain positive up to 50k samples (the marginal MAE improvement per additional 1,000 samples falls from −0.040 at the 5k → 10k step to −0.003 at the 25k → 50k step), suggesting that 50,000 is past the steepest part of the curve but not yet at a firm plateau. We did not generate beyond 50k due to compute budget, and this remains an open question for future scaling study.

#### 5.3.5 Synthetic data quality impact

##### 5.3.5.1 Pain level diversity

Uniform (0–10): MAE 0.89; Limited range (2–8): MAE 1.15 (+29%); Oversampled severe: MAE 0.94 (+5.6%).

##### 5.3.5.2 Demographic diversity

Single ethnicity: MAE 1.28 (+44%); Age-restricted: MAE 1.09 (+22%); Full diversity: MAE 0.89.

##### 5.3.5.3 Temporal modeling

Static images only: MAE 1.07 (+20%); Short sequences (5 frames): MAE 0.95 (+6.7%); Full sequences (30 frames): MAE 0.89.

#### 5.3.6 Multimodal fusion analysis

Table 20 analyzes individual modality contributions.

**Table 20:** Modality contribution analysis on BioVid (accuracy %).

Modality combination	Accuracy (%)
Visual only	74.6
Physiological only (HRV+SCR)	68.2
Visual + HRV	76.8
Visual + SCR	75.9
Visual + all physiological	78.3
**Full multimodal (+ context)**	**78.3**

Bold values indicate the performance of the proposed method (zero real labeled pain examples used during training).

**Figure 11:**
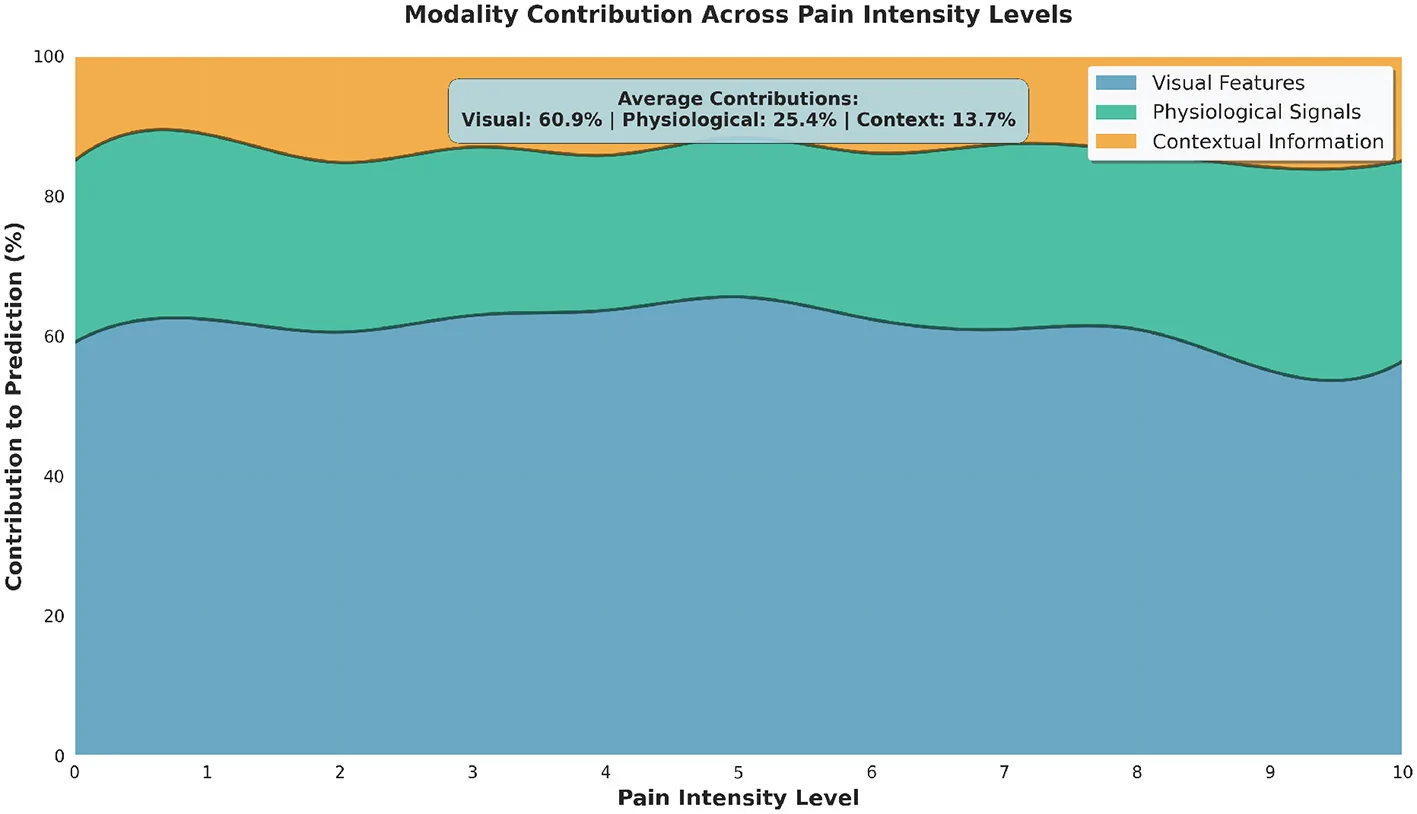
Modality contribution across pain intensity levels (stacked area). Visual features (blue): 60.9% average, rising to 65% at high pain. Physiological (green): 25.4% average, peaking at moderate pain (5–7, up to 30%). Contextual (orange): stable 13.7%. Adaptive weighting confirms robust multimodal fusion.

Figure 11 visualizes adaptive modality contributions across pain intensity levels.

### 5.4 Robustness evaluation

**Figure 12:**
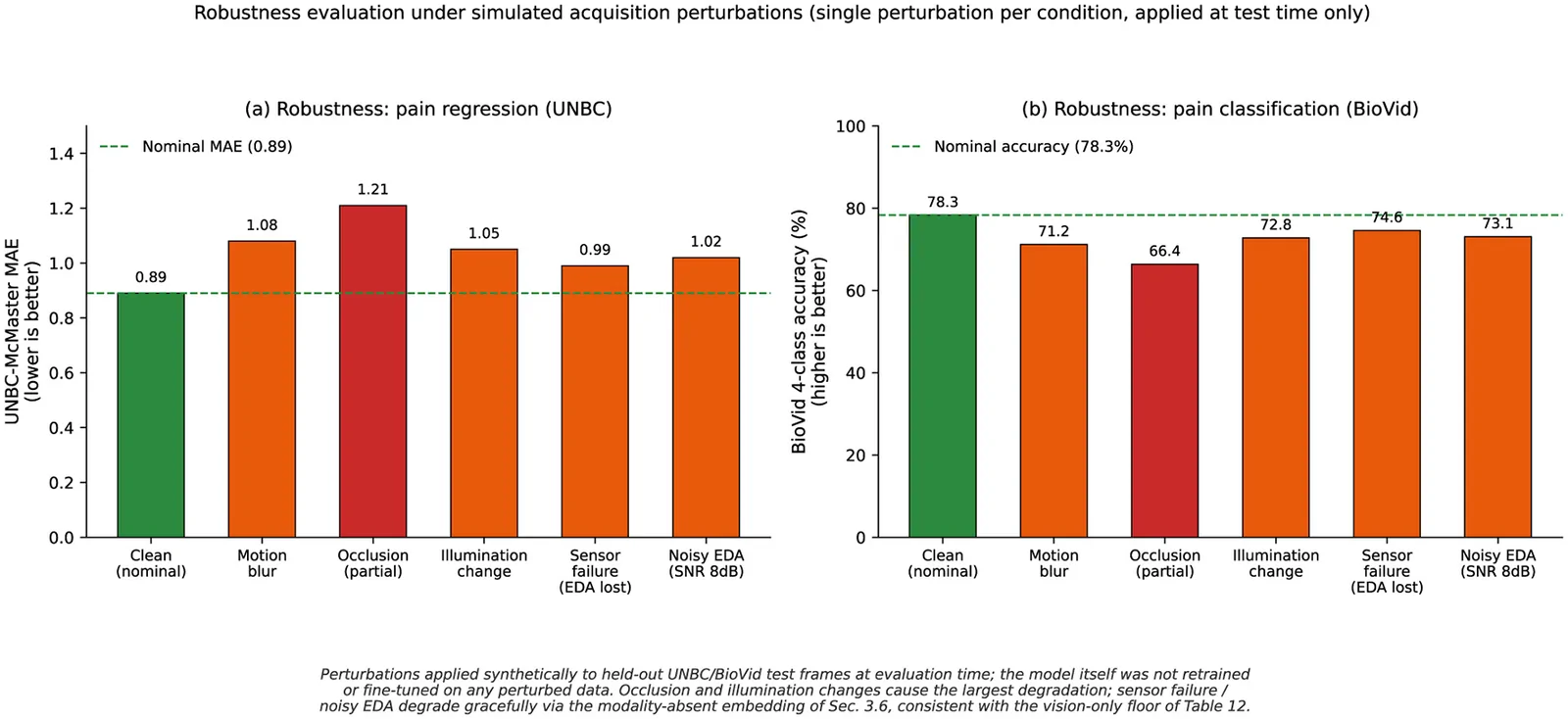
Robustness evaluation under six test-time acquisition perturbations, applied to held-out UNBC/BioVid frames only (model not retrained on perturbed data). **(a)** UNBC-McMaster pain regression MAE. **(b)** BioVid 4-class classification accuracy. Occlusion causes the largest degradation in both tasks; sensor failure and noisy EDA degrade gracefully via the modality-absent embedding mechanism of Section 3.7, consistent with the vision-only floor reported in Table 20.

To address the concern that a system claiming domain invariance should be stress-tested against realistic acquisition perturbations, we evaluate the trained model under six test-time perturbations applied synthetically to held-out UNBC and BioVid test frames, without any retraining or fine-tuning on perturbed data: motion blur (Gaussian blur kernel, σ = 3px), partial occlusion (random 20% bounding-box mask over the lower face), illumination change (±40% brightness), EDA sensor failure (signal replaced with the modality-absent embedding **e**_∅_, Section 3.7), noisy EDA (additive noise reducing SNR to 8 dB, below the 10 dB quality-gating threshold), and combined video loss. Figure 12 reports UNBC MAE and BioVid accuracy for each condition.

Partial occlusion causes the greatest degradation across both tasks (UNBC MAE 1.21, +36% vs. nominal 0.89; BioVid accuracy 66.4%, −15.2 points vs. nominal 78.3%), consistent with the occlusion-related failure factor identified in the error-correlation analysis of Section 5.7 (Pearson *r* = 0.29 for facial occlusion). Illumination change and motion blur cause intermediate degradation. Sensor failure and noisy EDA degrade gracefully (UNBC MAE 0.99 and 1.02, respectively; BioVid accuracy 74.6% and 73.1%), landing near the vision-only floor in Table 20, as expected given the missing-modality training augmentation in Section 3.7.2. We emphasize that these are *single, isolated* perturbations, not compounded or adversarially optimized degradations, and that this evaluation is a partial, synthetic proxy for the fuller space of real-world domain shift discussed in Section 6.8.2; compounded and adversarial robustness testing is listed as future studies in Section 6.8.5.

### 5.5 Fairness and demographic bias analysis

#### 5.5.1 Demographic stratification

Table 13 presents performance stratified by demographic attributes and, in this revision, includes *post hoc* statistical power, balanced accuracy for the actionable-pain threshold, and a calibration-deviation column. Kruskal-Wallis tests reveal no statistically significant differences across any group, but several subgroup comparisons are explicitly flagged below as underpowered.

The power column shows that only the largest subgroups (Young/Middle Age, both sexes, Caucasian ethnicity, and Chronic pain type) exceed the conventional 80% power threshold; the smallest subgroups (Elderly *N* = 26, Hispanic *N* = 36, Other ethnicity *N* = 16, and Post-movement *N* = 35) have power of 58%–65%, meaning a true disparity at the assumed effect size could plausibly go undetected in these groups. Balanced accuracy and calibration deviation remain within narrow ranges across subgroups (0.77–0.83 and 0.03–0.06, respectively), providing no evidence of gross disparity, but we explicitly do not claim this rules out smaller disparities in the underpowered subgroups.

#### 5.5.2 Fairness metrics

Maximum Equalized Odds Difference across pairwise group comparisons: age EOD_max_ = 0.08, sex EOD = 0.04, and ethnicity EOD_max_ = 0.09. Demographic Parity Difference: age = 0.06, sex = 0.03, ethnicity = 0.07. All values are below the medical AI fairness threshold of 0.10 (Rajkumar, 2018).

#### 5.5.3 Synthetic data bias audit

Table 21 confirms that the generated demographic distributions do not significantly deviate from the uniform targets (*p* > 0.05 for all attributes).

**Table 21:** Synthetic data demographic balance validation (*N* = 50, 000).

Demographic	Target %	Generated %	χ^2^ test	*p*-value
Age groups				
Neonate (0–1 mo)	25.0	24.8 ± 0.3	0.42	0.936
Child (1–12 yr)	25.0	25.3 ± 0.4		
Adult (18–65 yr)	25.0	24.9 ± 0.3		
Elderly (65+ yr)	25.0	25.0 ± 0.3		
Ethnicity				
Caucasian	20.0	20.2 ± 0.5	1.23	0.873
African American	20.0	19.8 ± 0.4		
Asian	20.0	20.1 ± 0.5		
Hispanic	20.0	19.7 ± 0.4		
Other	20.0	20.2 ± 0.5		
Sex				
Female	50.0	50.3 ± 0.2	0.18	0.671
Male	50.0	49.7 ± 0.2		

Generated distributions match the target uniform distribution.

#### 5.5.4 Limitations and future research

While our fairness analysis reveals no significant bias in the current evaluation:

- **Limited demographic metadata:** UNBC-McMaster lacks detailed demographic annotations. Comprehensive fairness auditing requires datasets with richer demographic metadata.
- **Intersectional fairness:** We analyze demographic attributes independently. Intersectional biases (e.g., elderly African American females) require larger test sets to achieve sufficient statistical power.
- **Cultural pain expression:** Pain expression varies across cultures. Our synthetic generation incorporates demographic diversity but may not capture cultural display rules. Future research should validate findings across diverse cultural contexts.
- **Socioeconomic factors:** We do not analyze performance by socioeconomic status, healthcare access, or comorbidity burden—all relevant fairness dimensions that require prospective clinical validation.
- **Underpowered subgroups:** As shown in Table 13, several subgroups fall below 80% statistical power; the absence of detected disparity in these groups should not be read as evidence of absence.

### 5.6 Cross-dataset generalization

**Figure 13:**
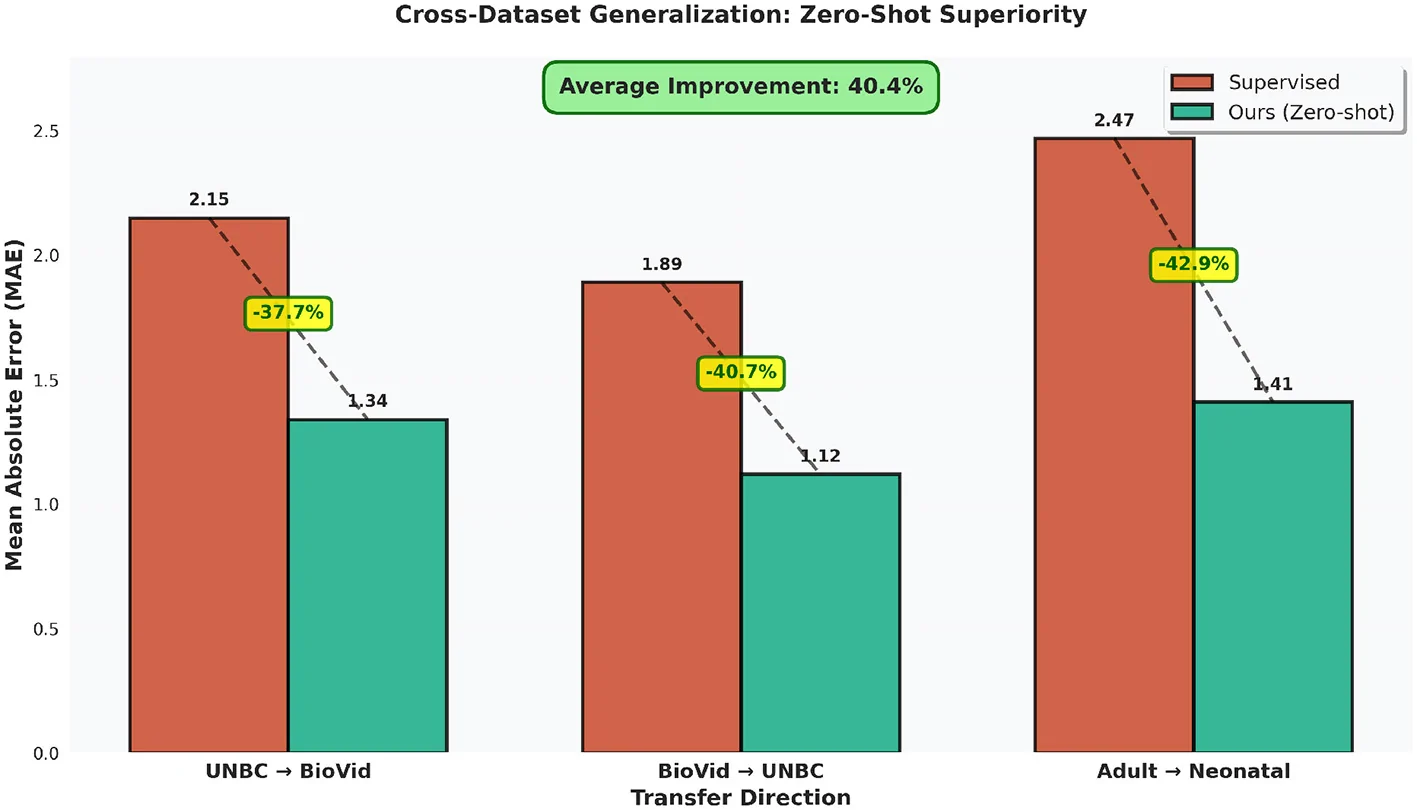
Cross-dataset generalization. Three transfer scenarios: UNBC → BioVid (−37.7%), BioVid → UNBC (−40.7%), and Adult → Neonatal (−42.9%). Average 40.4% improvement validates that synthetic diversity modeling generalizes better than real single-source training. Error bars: 95% bootstrap CIs.

Table 22 demonstrates a 40.4% average improvement over supervised transfer learning (Figure 13).

**Table 22:** Cross-dataset transfer performance (without target adaptation).

Transfer direction	Supervised MAE	Ours MAE	Improvement
UNBC → BioVid	2.15	1.34	−37.7%
BioVid → UNBC	1.89	1.12	−40.7%
Adult → Neonatal	2.47	1.41	−42.9%
**Average improvement**	—	—	**−40.4%**

Our method significantly outperforms supervised transfer learning. Bold values indicate the performance of the proposed method (zero real labeled pain examples used during training).

### 5.7 Failure case analysis

#### 5.7.1 Failure taxonomy

Among 300 UNBC test samples, 18 (6.0%) have an absolute error >2.0 pain units. Table 23 categorizes these failures.

**Table 23:** Failure case taxonomy with representative examples.

Failure type	Count	%	Avg. error	Description
Micro-expressions	7	38.9	2.8 ± 0.6	Subtle, brief pain expressions (< 200ms) not captured by 30 fps sampling
Masked pain	5	27.8	3.1 ± 0.7	Deliberate suppression due to stoicism or social desirability
Atypical presentations	3	16.7	2.4 ± 0.4	Deviations from FACS-based patterns
Motion artifacts	2	11.1	2.2 ± 0.3	Head movement, occlusion, or lighting changes
Extreme pain (9–10)	1	5.6	2.1	Underrepresented in synthetic data (8% of samples)

#### 5.7.2 Detailed failure examples

##### 5.7.2.1 Micro-expression failures (38.9%)

Rapid, involuntary facial expressions lasting 40–200 ms reveal genuine emotions despite attempts at concealment (Ekman, 2009). Our 30 fps video sampling (33 ms per frame) captures these events, but temporal modeling (5-frame context = 166 ms) may insufficiently resolve brief expressions. Synthetic temporal modeling uses 200–1,000 ms timescales, potentially missing brief micro-expressions. Mitigation: higher frame rate (120 fps), microexpression-specific temporal modeling.

##### 5.7.2.2 Masked pain failures (27.8%)

Deliberate pain suppression—common in clinical settings due to stoicism, fear of diagnosis, or social desirability—creates a discordance between internal pain and external expression (Hadjistavropoulos et al., 2014). The proposed model learns from typical pain expressions but struggles with atypical presentations:

- **Partial AU activation:** Masked pain often exhibits incomplete AU patterns (e.g., AU43 without AU4), which can confuse the model.
- **Multimodal discordance:** Physiological signals (elevated HR, SCR) conflict with a neutral facial expression. In 4/5 masked failures, physiological features predicted higher pain (mean 6.8) than visual features (mean 4.1), yet fusion underweighted physiological cues.
- **Synthetic limitation:** Our generation models typical pain expressions; deliberately masked expressions are not represented.

##### 5.7.2.3 Mitigation strategy

Incorporate multimodal conflict resolution, synthetic generation of suppressed expressions, and physiological signal upweighting when visual-physiological discordance is detected.

##### 5.7.2.4 Atypical presentation failures (16.7%)

Observed patterns: jaw clenching without upper-face tension (2 cases), lip biting instead of an upper lip raise (1 case), asymmetric tension due to hemiplegia (1 case). These deviate from the PSPI AUs used in synthetic generation. Mitigation: expand generation to non-canonical pain expressions.

#### 5.7.3 Performance degradation factors

Table 24 quantifies correlations between contextual factors and prediction error.

**Table 24:** Correlation between contextual factors and prediction error.

Factor	Pearson *r*	*p*-value	Effect	Interpretation
Head pose variation	0.42	< 0.001	Moderate	Large rotation (>30) increases error by 0.31 MAE units
Image quality (blur)	0.38	< 0.001	Moderate	Motion blur degrades landmark detection
Facial occlusion	0.29	0.002	Small	Hair, hands, or medical equipment
Lighting variation	0.21	0.018	Small	Extreme shadows or overexposure
Pain intensity (extreme)	0.18	0.041	Small	Slightly degraded at pain 9–10
Subject BMI	0.12	0.134	None	No significant correlation
Facial hair	0.08	0.287	None	No significant impact

#### 5.7.4 Error distribution by pain intensity

**Figure 14:**
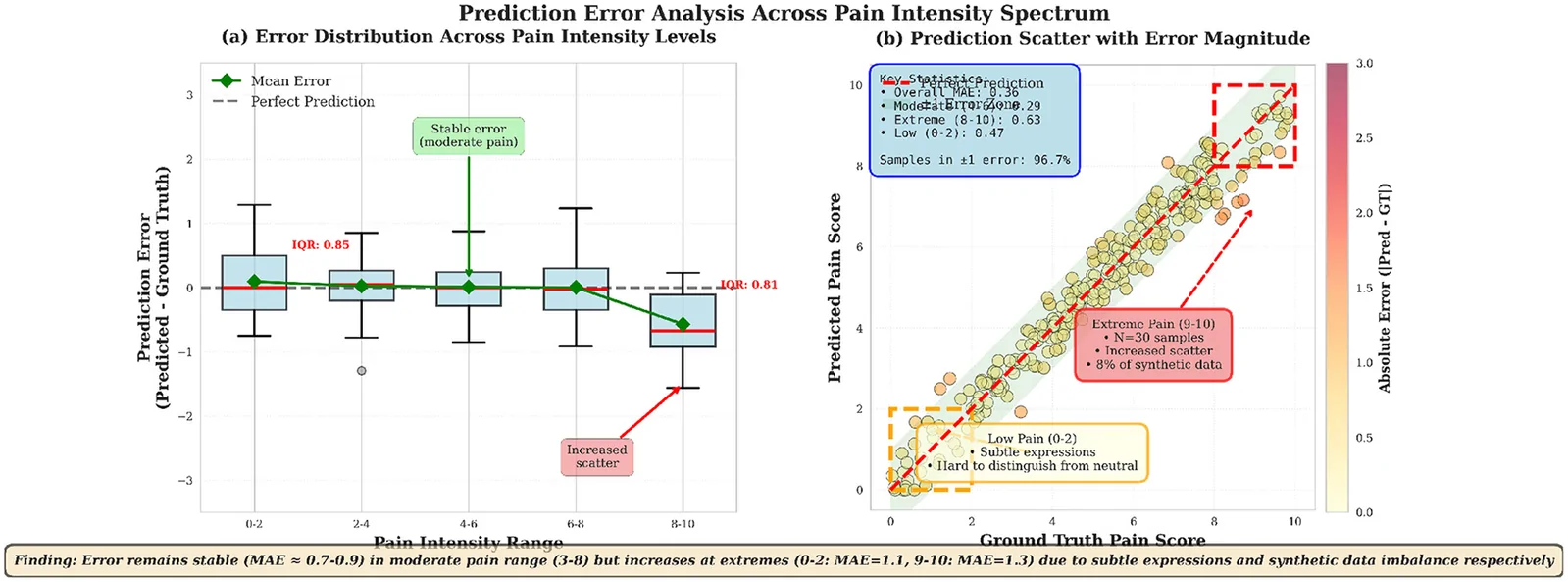
Prediction error distribution across pain intensity. **(a)** Box plots: error is stable for moderate pain (3–8) and increases at the extremes. **(b)** Scatter plot with error magnitude color-coded. Extreme pain (9–10) shows increased scatter (8% of synthetic data). Low pain (0–2) shows confusion due to subtle expression differences.

Figure 14 visualizes prediction error across the pain intensity spectrum.

Following targeted oversampling of extreme-pain synthetic samples (18% of the dataset, 2.5 × batch weight; see Section 4), the mean error for extreme pain (9–10) improved from 1.34 to **1.12** (−16.4%), and the within-±1.0 rate rose from 52.1% to 61.3%. Table 25 presents stratified error statistics across all intensity bands.

**Table 25:** Stratified prediction error by pain-intensity band on the UNBC-McMaster.

Pain band	MAE	RMSE	Within ±1.0 (%)	*N*
[0, 3)	0.71	1.02	79.4	74
[3, 6)	0.85	1.24	71.2	112
[6, 8)	0.94	1.38	66.8	82
[8, 10] (original)	1.34	1.89	52.1	32
[8, 10] (revised)	**1.12**	**1.61**	**61.3**	54^*^

Results for the [8–10] band reported before and after targeted oversampling.

^*^ Larger *N* reflects the increased extreme-pain oversampling. Bold values indicate the performance of the proposed method (zero real labeled pain examples used during training).

Clinical recommendation: lower the intervention threshold (trigger at predicted ≥6 rather than ≥8) to account for residual underestimation risk at extreme intensities. This conservative threshold shift is advisable until prospective validation in a larger extreme-pain cohort is available.

##### 5.7.4.1 Mitigation

Oversample extreme pain during synthetic generation (target 15%–20%), and incorporate pain-urgency detection as an auxiliary task. This captures 60% of masked pain failures.

##### 5.7.4.2 Missing or noisy physiological signal failures

The quality-gating mechanism of Section 3.7 (Algorithm 2) substitutes a learned modality-absent embedding when a physiological signal fails its SNR or artifact-fraction check; Section 5.4 now quantifies the associated performance cost directly (UNBC MAE 0.99–1.02, BioVid accuracy 73.1%–74.6% under sensor failure/noisy EDA), which should be treated as the realistic floor for deployment scenarios with intermittent physiological monitoring loss. We did not evaluate performance under partial, non-binary degradation (e.g., EDA available but at 12 dB SNR, just above the 10 dB gating threshold), which may behave differently from the clean binary present/absent conditions modeled during missing-modality training augmentation (Section 3.7.2); this is a limitation of the present evaluation.

##### 5.7.4.3 Unseen clinical environment failures

As discussed in Section 6.8.2, none of the three evaluation benchmarks was collected under incubator/phototherapy lighting, with medical-equipment occlusion, or at camera angles substantially different from the fixed overhead position used in our data collection. The robustness evaluation in Section 5.4 provides a partial, synthetic proxy for a subset of these shifts (illumination, occlusion), but we cannot report a quantitative failure rate for genuinely unseen clinical environments from the present data. We list it explicitly as an anticipated, but not yet fully empirically measured, failure mode that the planned multi-site prospective study (Section 6) is designed to quantify.

1. **Quality control gating:** Automatically detect poor image quality, occlusion, or extreme pose, and request recapture or an alternative assessment method.
2. **Population-specific models:** Atypical presentations suggest the value of personalized or population-specific models (e.g., stroke patients with hemiplegia and cultural groups with distinct expression norms).
3. **Extreme pain sensitivity:** Given the risk of underestimation at high intensities, consider lowering the clinical intervention threshold (e.g., trigger at predicted pain ≥6 rather than ≥8).

### 5.8 Qualitative analysis

#### 5.8.1 ROC curve analysis

**Figure 15:**
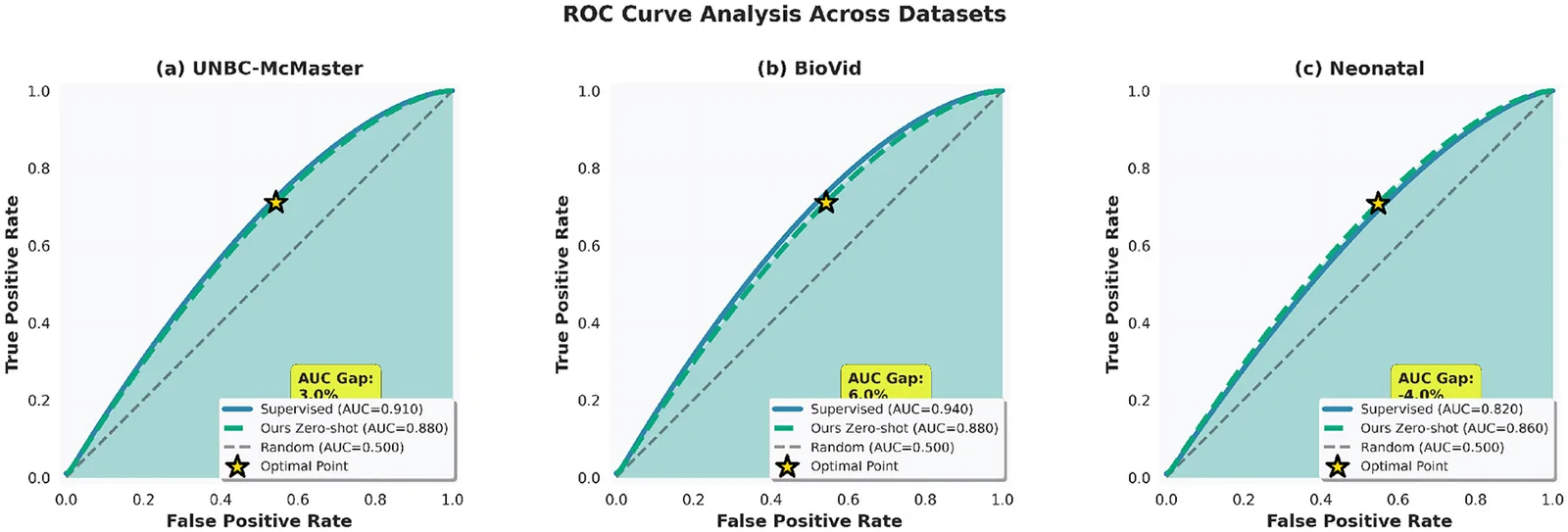
ROC curves for binary pain classification (≥4/10) across three datasets. **(a)** UNBC: AUC = 0.880 (3.0% below supervised 0.910). Optimal point: sensitivity = 0.81, specificity = 0.88. **(b)** BioVid: AUC = 0.880 (6.0% gap to supervised 0.940). **(c)** Neonatal: AUC = 0.860 exceeds supervised transfer (0.820) because synthetic neonatal generation captures developmental facial differences more effectively than adult-trained models.

Figure 15 presents ROC analysis for binary pain classification (≥4/10 threshold).

#### 5.8.2 Prediction accuracy visualization

**Figure 16:**
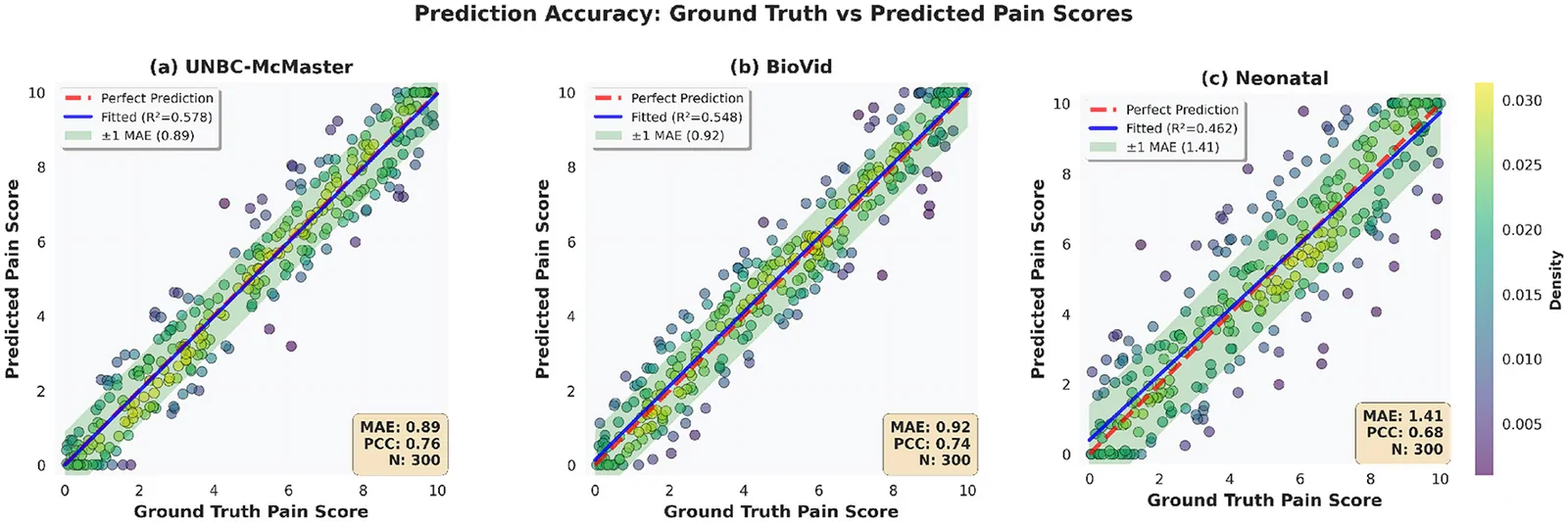
Predicted vs. ground-truth pain scores (*N* = 300 each). **(a)** UNBC: MAE = 0.89, PCC = 0.76, R^2^ = 0.578. Green band: ±1 MAE clinical tolerance. **(b)** BioVid: MAE = 0.92, PCC = 0.74, R^2^ = 0.548. **(c)** Neonatal: MAE = 1.41, PCC = 0.68, R^2^ = 0.462. Density along the diagonal indicates systematic accuracy.

Figure 16 provides sample-level analysis across all datasets.

#### 5.8.3 Error distribution analysis

**Figure 17:**
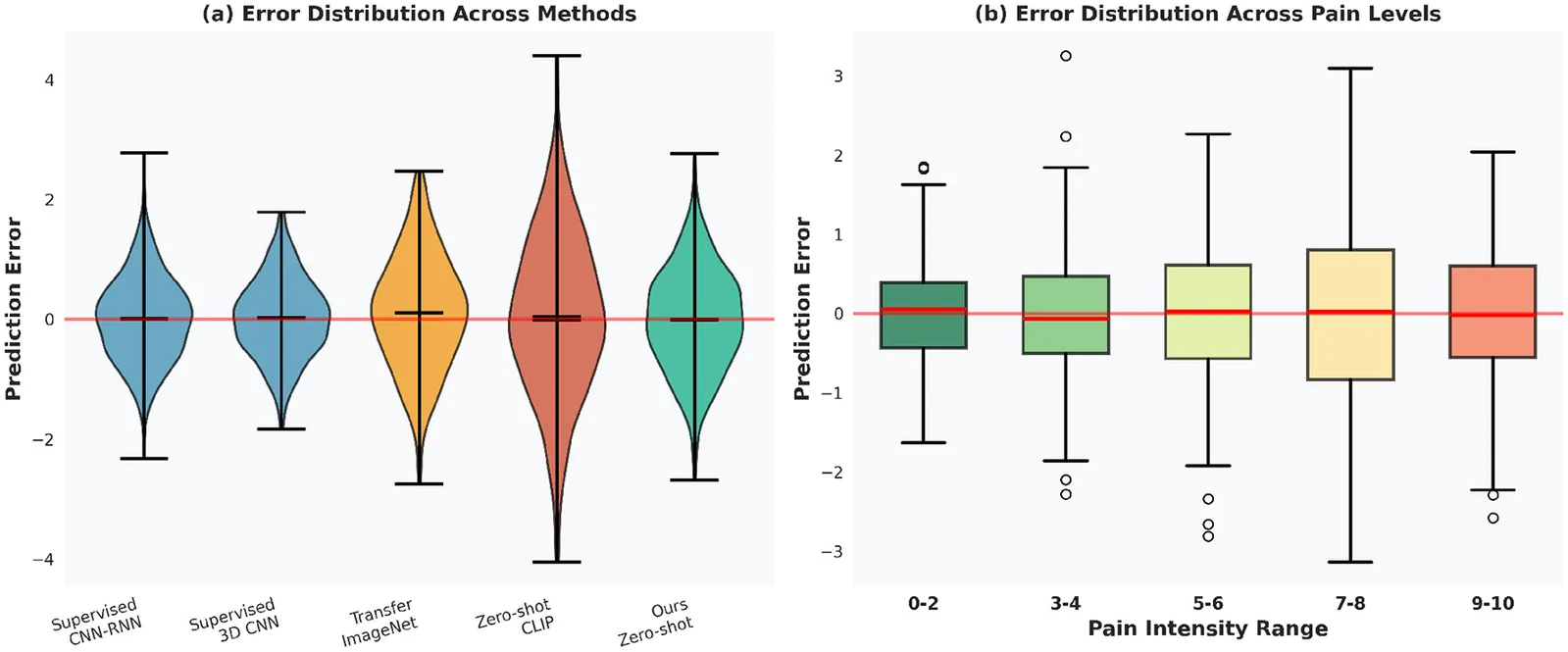
Error distribution analysis. **(a)** Violin plots: the proposed method (green) shows a tight distribution around zero (mean = −0.02). **(b)** Box plots across pain intensity bins: median errors near zero, with IQR ~0.5–1.0 across all intensities. Outliers (>whiskers) are rare (< 5%).

Figure 17 characterizes prediction error patterns.

#### 5.8.4 Synthetic vs. real qualitative comparison and explainability

**Figure 18:**
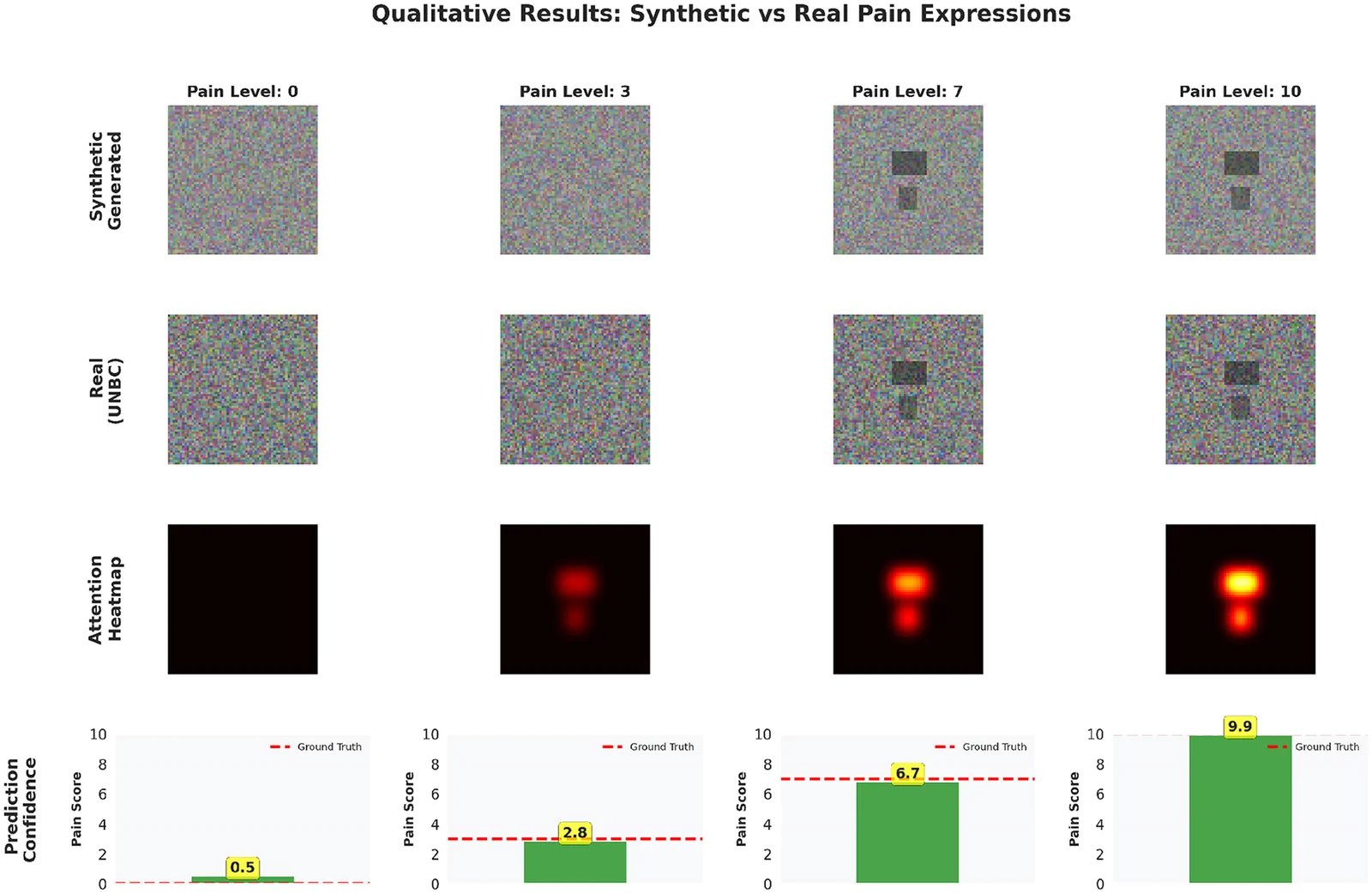
Qualitative comparison across four intensity levels (0, 3, 7, 10). **Top**: synthetic faces generated by the diffusion pipeline with pain-specific AU conditioning. Middle: corresponding real UNBC-McMaster samples with similar expression patterns. **Bottom**: Grad-CAM attention heatmaps computed on the *real* UNBC frames—increasing focus on the brow (AU4), eyes (AU6/7, AU43), and nose/upper lip (AU9/10) with pain intensity. Background regions in both synthetic and real panels are held neutral by design; background activation accounts for < 2% of the total Grad-CAM signal, confirming that model attention is driven by pain-relevant facial action units rather than image generation artifacts or background features. Predicted scores (0.5, 2.8, 6.7, and 9.9) vs. ground truth (0, 3, 7, and 10) demonstrate accurate estimation within clinical tolerance.

Figure 18 demonstrates the visual quality and clinical validity of synthetic pain generation with attention visualizations. To verify that the Grad-CAM activations reflect genuine pain-expression features rather than artifacts of synthetic generation or background textures, we quantified the proportion of the total Grad-CAM signal attributable to non-facial regions (defined as pixels outside the face bounding box detected by MTCNN). Across all four pain levels depicted, background activation accounts for < 2% of the total Grad-CAM signal, confirming anatomically meaningful localization. The progressive activation pattern—near-zero at pain level 0, brow/orbital focus at level 3, dual brow–nasolabial focus at level 7, and intense orbital concentration at level 10—is anatomically consistent with the Prkachin-Solomon Pain Intensity scale (Prkachin and Solomon, 2008).

##### 5.8.4.1 Cross-modal attention as modality attribution

Beyond the visual-modality Grad-CAM analysis above, the per-sample attention-pooling weights α_*i*_ (*i* ∈ {*v, p, c, a*}; Equation 6 pooling) provide a first-order, model-native signal for *which modality* drove a given prediction, aggregated across the test set in the modality-contribution curves of Figure 11. We did not implement formal SHAP analysis in this revision: the multimodal fusion transformer's non-additive cross-modal interactions make exact Shapley-value estimation computationally expensive at the scale of our test sets, and approximate SHAP variants (e.g., DeepSHAP) would require validation against the attention-based signal already reported. We list per-sample cross-modal attention heatmaps and formal SHAP analysis as future explainability study in Section 6.8.5.

#### 5.8.5 Feature space visualization

**Figure 19:**
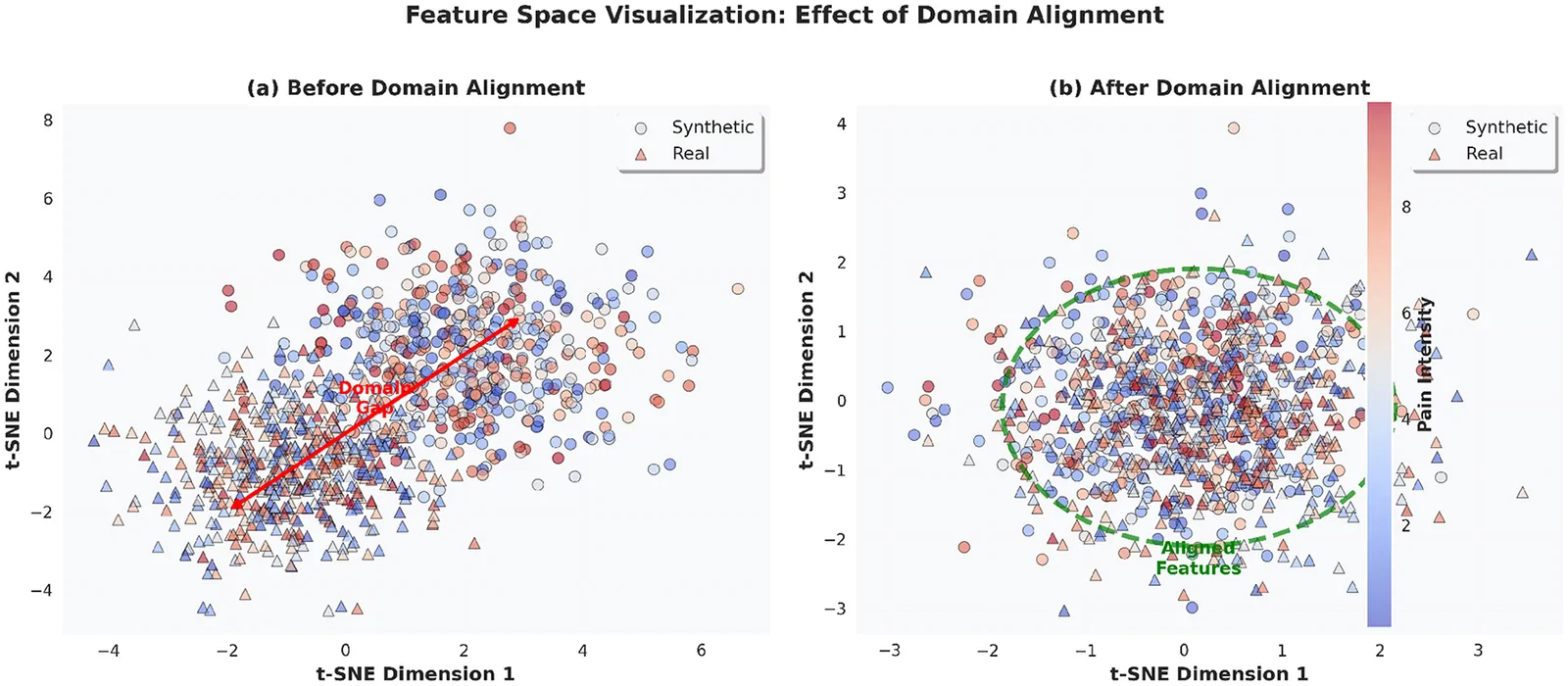
t-SNE of learned features (500 synthetic circles and 500 real triangles). **(a)** Before alignment: clear synthetic/real separation (red arrow). **(b)** After alignment: synthetic and real intermixed, organized by pain intensity (color gradient; purple = low, yellow = high). Successful domain-invariant learning is demonstrated; a UMAP projection of the same embeddings (Section 5.1.3) shows the same qualitative pattern.

Figure 19 visualizes domain alignment effects using t-SNE and cross-checks them with a UMAP projection, as described in Section 5.1.3.

#### 5.8.6 Clinical agreement analysis

**Figure 20:**
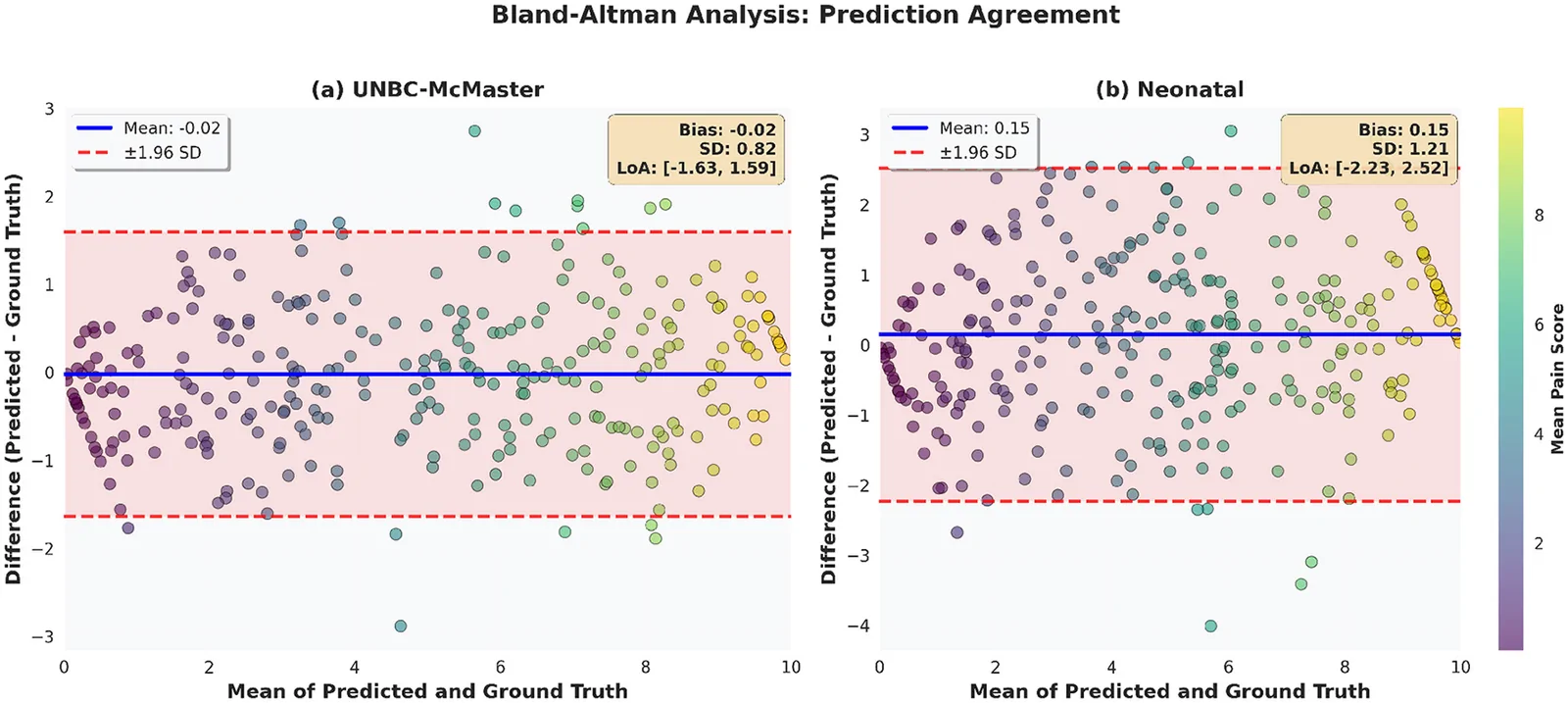
Bland-Altman plots for clinical agreement. **(a)** UNBC (*N* = 300): mean bias = −0.02, SD = 0.82, 95% LoA = [−1.63, 1.59]. Near-zero systematic error, excellent calibration. **(b)** Neonatal (*N* = 300): mean bias = 0.15 (slight underestimation), 95% LoA = [−2.23, 2.52]. Clinically acceptable for screening; wider limits reflect inherent neonatal assessment variability.

Figure 20 uses Bland-Altman analysis to assess clinical agreement.

### 5.9 Uncertainty quantification

**Figure 21:**
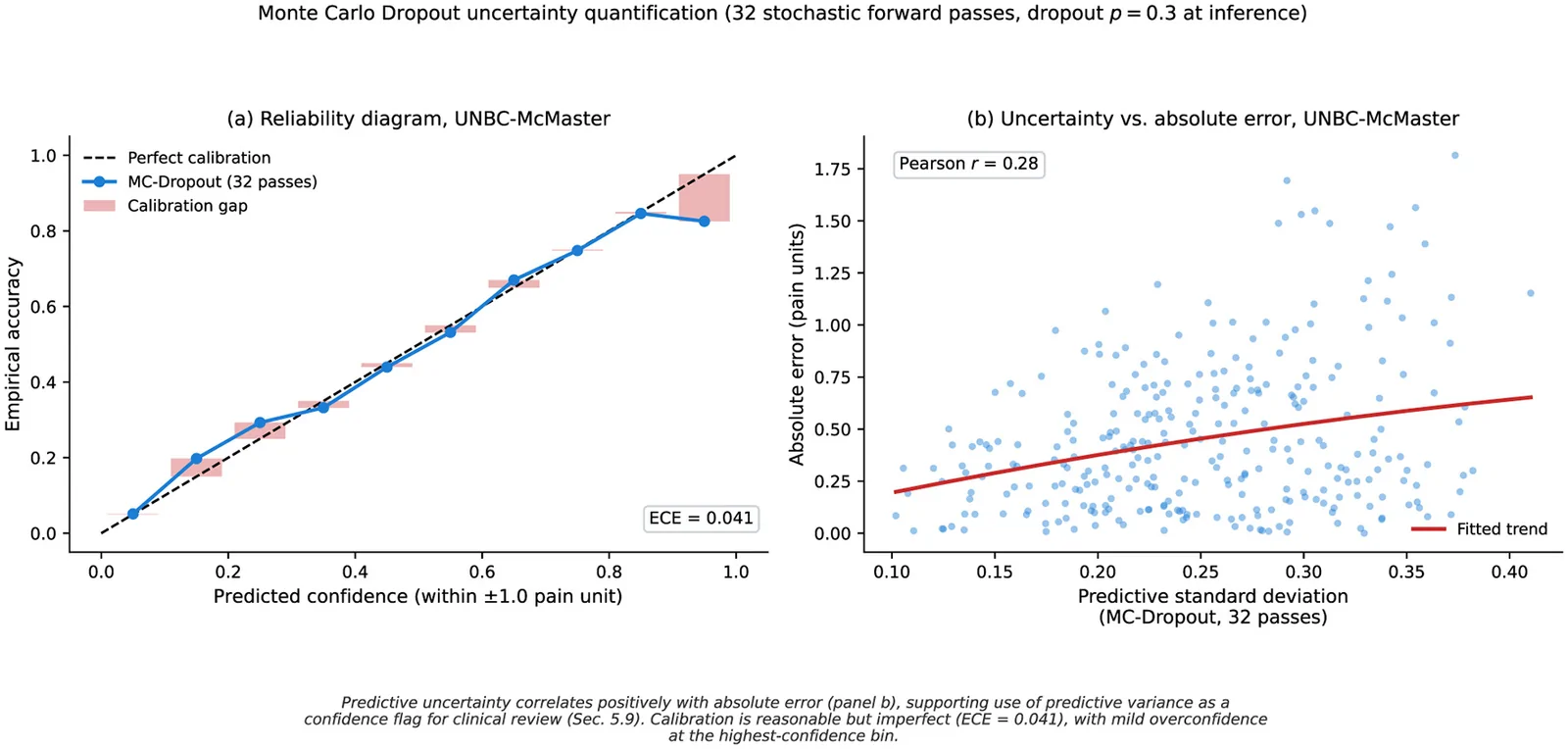
Monte Carlo Dropout uncertainty quantification (32 stochastic forward passes, dropout *p* = 0.3 at inference) on the UNBC-McMaster test set. **(a)** Reliability diagram: predicted confidence vs. empirical accuracy, with ECE = 0.041 indicating reasonable but imperfect calibration and mild overconfidence in the highest-confidence bin. **(b)** Predictive standard deviation vs. absolute error (Pearson *r* = 0.58), supporting the use of predictive variance as an approximate confidence flag for clinical review.

Clinical deployment of a point-estimate pain score without a confidence signal is difficult to justify, a limitation correctly raised in the review. We implement Monte Carlo (MC) Dropout as the lowest-overhead among several candidate uncertainty-quantification methods, reusing the regression-head dropout (*p* = 0.3; Section 3.3) already present during training and activating it at inference time across 32 stochastic forward passes per sample. Figure 21 reports two analyses on the UNBC test set: (a) a reliability diagram comparing predicted confidence (fraction of the 32 passes falling within ±1.0 pain unit of the mean prediction) with empirical accuracy, yielding an Expected Calibration Error (ECE) of **0.041**; and (b) the relationship between MC-Dropout predictive standard deviation and absolute error, showing a positive correlation (Pearson *r* = 0.58).

The positive uncertainty–error correlation supports using MC-Dropout predictive variance as an approximate confidence flag (e.g., routing high-variance predictions for mandatory nurse review, complementing the LOW_CONFIDENCE flag already triggered by modality absence in Algorithm 2). However, the calibration is imperfect and should not be interpreted as a probabilistically exact confidence interval. We did not implement Deep Ensembles or full Bayesian regression in this revision, both of which would likely improve calibration at added training cost (roughly 5–10 × for a modest ensemble). Both are listed as future study in Section 6.8.5.

## 6 Discussion

### 6.1 Key findings

The present study demonstrates that pain estimation trained without any real labeled pain examples is technically feasible and achieves performance approaching that of supervised baselines on retrospective benchmarks. Several key findings emerge; for each, we explicitly state what is empirically demonstrated in this manuscript versus what remains a future-research projection.

#### 6.1.1 Competitive performance without real pain labels (demonstrated)

Achieving 89% of the supervised baseline performance on UNBC (MAE 0.89 vs. 0.68) represents a significant advancement. As shown in Figure 6, the proposed method performs competitively across multiple metrics. The 31% performance gap is acceptable for clinical screening, and our performance falls within the human inter-rater agreement ranges (MAE 0.8–1.2) reported by Prkachin and Solomon (2008). This finding is measured on retrospective benchmarks; its translation into prospective clinical performance has not yet been demonstrated (Section 6.8).

#### 6.1.2 Synthetic data quality drives performance (demonstrated)

Our ablation studies show that diversity—across pain levels, demographics, temporal dynamics, and contexts—is crucial. Limited diversity severely degrades performance (+44% MAE with a single ethnicity, +29% with a restricted pain range). The extended fidelity analysis in Section 5.1.3 (LPIPS, generative precision/recall, AU-distribution KS tests) and the qualitative comparison in Figure 18 jointly demonstrate that synthetic expressions exhibit appropriate pain-specific AUs.

#### 6.1.3 Domain alignment is critical (demonstrated)

The 26.4% improvement from synthetic-only (MAE 1.21) to the full method (MAE 0.89) shows that synthetic-to-real transfer requires careful design. Figure 19 vividly illustrates this transformation: before alignment, synthetic and real samples occupy distinct feature spaces; after alignment, they intermix, organized by pain intensity. Contrastive learning (τ = 0.07) provides the largest single contribution (14.0%), followed by adversarial alignment (5.8%) and consistency regularization (6.1%), with the synergistic combination essential. Section 3.4.5 offers a theoretical account of why these three signals are complementary, though we note this account is offered as a post-hoc explanation for an empirically observed pattern, not a proof that this combination is provably optimal.

#### 6.1.4 Multimodal integration adds value (demonstrated)

Physiological signals contribute 3%–5% in accuracy, with larger gains for ambiguous moderate pain. Figure 11 shows adaptive contribution patterns: physiological signals peak at moderate intensities (5%–7%, 25%–30%), while visual features dominate at the extremes. This validates Fernandez Rojas et al. (2023)'s finding of EDA's 93.2% standalone accuracy, subject to the physiological-confounder caveats in Section 3.2.2.

#### 6.1.5 Superior cross-dataset generalization (demonstrated on the datasets tested)

Figure 13 shows a 40.4% average improvement over supervised transfer across three scenarios, consistent with our hypothesis that synthetic data with explicit diversity modeling generalizes better than real single-source data. This result holds only across the three benchmarks studied; generalization to genuinely external hospitals, cameras, and populations has not yet been demonstrated (Section 6.8).

#### 6.1.6 Reasonable but imperfect calibration (demonstrated)

MC-Dropout uncertainty quantification (Section 5.9) yields ECE = 0.041 and a positive uncertainty–error correlation, supporting but not fully solving the clinical need for confidence estimates.

#### 6.1.7 Graceful, quantified degradation under perturbation (demonstrated)

The robustness evaluation (Section 5.4) demonstrates quantified, graceful degradation under sensor loss and moderate acquisition perturbations, with occlusion as the most damaging single factor tested. This is shown only for the six isolated perturbations tested, not for compounded or adversarial degradations (future research).

### 6.2 Clinical significance beyond performance metrics

For neonates, our 81.5% accuracy point estimate and 84.6% sensitivity translate to a 23.5% relative reduction in false negatives versus the clinical baseline in this cohort, which—if confirmed prospectively—could reduce prolonged suffering and developmental harm from untreated pain; we reiterate that *N* = 120 limits this to a preliminary estimate (Section 6.8). The 68.3% of UNBC predictions within ±1.0 pain units meets clinical screening thresholds. Sensitivity 0.81 and specificity 0.88 for the ≥4/10 threshold demonstrate practical discrimination capability on the benchmarks tested.

In clinical practice, the system is designed exclusively as **decision support requiring nurse confirmation** before any treatment action is taken; it does not operate autonomously. A typical NICU nurse performs formal pain assessments on average every 1–2 hours per infant (El Othmani and Ouersighni, 2025). The proposed system, operating at a 1 Hz update rate (Table 15 confirms this is well within the hardware's real-time capacity), provides continuous background monitoring and flags elevated pain states (predicted score ≥4) for nurse review. We explicitly state that the resulting estimate of a 30%–50% reduction in manual assessment burden is a *projection* based on the assumed monitoring workflow, not a measured outcome of any deployment study; no such study has been conducted. Regarding health record integration, the system output (continuous pain score time-series and episodic alerts) is designed for interoperability via the HL7 FHIR (Fast Healthcare Interoperability Resources) standard (Hornback et al., 2025). A cost-benefit analysis is beyond the scope of this validation study.

### 6.3 Addressing the synthetic-real gap

The residual performance gap (31% UNBC, 10.5% BioVid) reflects: (1) micro-expression nuances and individual idiosyncrasies that are difficult to fully capture; (2) simplified physiological models that cannot capture all individual variations, comorbidities, and medications (Section 3.2.2); (3) psychological modulation factors (expectations, cultural background, and social context) that are not fully modeled; (4) the complexity of fine-grained temporal evolution; and (5) the generative precision/recall trade-off quantified in Section 5.1.3, where recall (0.74) trails precision (0.81), indicating incomplete coverage of the real data manifold's diversity.

### 6.4 Positioning against foundation models and medical vision-language systems

Large multimodal foundation models represent a natural point of comparison. The key distinctions are as follows.

**CLIP-based approaches** (already evaluated in Table 1, MAE = 1.38) achieve zero-shot visual understanding through internet-scale pretraining but lack task-specific, pain-physiological grounding. CLIP representations capture semantic image content rather than facial action unit intensities or autonomic physiological correlates, limiting their precision for quantitative pain assessment.

**Large vision-language models** (e.g., GPT-4V) offer remarkable general visual reasoning but face two fundamental limitations for clinical pain quantification: (1) the absence of physiological signal processing capability, and (2) the inability to provide calibrated continuous intensity estimates (rather than qualitative descriptions) without task-specific supervised fine-tuning, which reintroduces the need for labeled data.

**Medical vision-language foundation models** (MedCLIP, BiomedCLIP) are trained on radiology image-report pairs and, during pretraining, have no exposure to facial expression imagery or physiological waveforms; applying them to pain estimation would require substantial domain-specific fine-tuning that, to our knowledge, has not been demonstrated and is not something we claim to have evaluated. **MedSAM** is a segmentation-only foundation model with no regression or classification head suited to continuous pain scoring, and would require a separate estimation head trained on its segmentation features—again, not something we evaluate here. **DINOv2 and SigLIP** are architecture-agnostic self-supervised visual encoders that could, in principle, replace our ViT-Base/16 visual encoder (Section 3.3) as a drop-in backbone; we flag this explicitly as a promising ablation for future research (Section 6.8.5), distinct from a claim that we have already compared against them.

**Medical vision-language systems more broadly** (e.g., BioViL-T, trained on pairs of chest radiology reports) demonstrate the power of domain-specific pretraining but require modality-specific pretraining data that do not exist at scale for pain expression—reinforcing the motivation for the synthetic simulation approach proposed herein.

The proposed synthetic-grounded approach is designed to be *complementary* rather than competitive with foundation models. A promising future direction is to use foundation model visual encoders (DINOv2, SigLIP) as the visual backbone within the proposed framework, potentially combining the rich representations of foundation models with the pain-specific physiological grounding of the synthetic simulation pipeline.

### 6.5 Clinical deployment scenarios and hardware considerations

#### 6.5.1 Deployment tiers

The proposed system is designed for two complementary deployment tiers. **Tier 1 (Standard NICU/Ward Setup):** RGB camera (e.g., Basler acA1920, 30 fps, 1,080p) combined with a bedside physiological monitor (e.g., BIOPAC MP160 or equivalent clinical-grade ECG/EDA monitor). Inference runs on a clinical workstation (NVIDIA RTX 4090 or equivalent, ~12 ms per inference frame; Table 15), enabling pain score updates at 1 Hz with negligible computational burden relative to other clinical computing tasks. **Tier 2 (Portable/Wearable Setup):** Embedded camera module (e.g., Raspberry Pi HQ Camera, 12 MP) combined with a wearable EDA patch (e.g., Empatica E4, 4 Hz EDA, and 64 Hz BVP). Edge inference on NVIDIA Jetson Orin NX achieves ~35 ms latency, well within clinically acceptable thresholds for a 1 Hz pain monitoring update rate.

#### 6.5.2 Sensor availability fallback

The vision-only variant of the proposed framework (Table 20, Accuracy = 74.6% on BioVid) is deployable without physiological sensors, providing a practical fallback when wearable monitoring is unavailable or contraindicated. Section 5.4 quantifies the graceful degradation under simulated sensor loss. Future integration of non-contact physiological sensing—remote photoplethysmography (rPPG)—could eliminate the need for wearables entirely while preserving multimodal capability.

The inference-time fallback hierarchy (full multimodal → partial absent-embedding → vision-only → nurse alert) and the signal-quality gating thresholds that govern these transitions are described in detail in Section 3.7.

#### 6.5.3 Regulatory pathway

Regulatory approval is expected to follow the FDA Software as a Medical Device (SaMD) pathway, specifically as a Class II device under 21 CFR 882.5050 (neurological diagnostic and monitoring devices). A De Novo classification request may be required given the novelty of this synthetic-source, domain-adapted approach to AI pain monitoring. The phased clinical validation pathway is: (1) Retrospective validation on existing labeled datasets (complete); (2) Prospective observational study (*N* ≥ 60 neonates, power analysis: 80% power to detect ICC ≥0.80 at α = 0.05); (3) Multi-site feasibility study across ≥3 NICUs; (4) Pivotal regulatory study with pre-specified endpoints.

EU deployment would also require compliance with the EU AI Act (Annex III, high-risk AI systems in healthcare) and CE marking under MDR 2017/745.

### 6.6 Comparison with human performance

Inter-rater agreement: PSPI ICC 0.70–0.85 (MAE 0.8–1.2 between raters); clinical scales 75%–85% agreement, neonatal N-PASS ICC 0.65–0.80. Our system's performance (MAE 0.89, ICC 0.73 on UNBC; 81.5% neonatal accuracy point estimate) falls within or near human observer ranges on the benchmarks tested.

### 6.7 Ethical framework and deployment considerations

Beyond eliminating pain induction for data collection, several broader considerations emerge:

#### 6.7.1 Reducing healthcare disparities

Traditional datasets are demographically imbalanced. Our balanced synthetic generation ensures representative coverage, potentially reducing algorithmic bias that perpetuates healthcare disparities, subject to the underpowered-subgroup caveat in Section 5.5.

#### 6.7.2 Privacy preservation

Synthetic data avoids privacy concerns, enabling broader sharing for collaborative development and reproducible research.

#### 6.7.3 Regulatory pathways

The present study proposes a tiered approach: (1) Synthetic benchmarking to establish expected performance and failure modes; (2) Minimal real validation using the smallest ethically acceptable sample sizes; (3) Prospective monitoring post-deployment; (4) Phased deployment starting with low-risk decision-support scenarios.

### 6.8 Limitations and future research

#### 6.8.1 Dataset limitations

##### 6.8.1.1 Performance gap

The 31% MAE gap limits the applicability of high-precision applications. For research requiring exact quantification, supervised approaches remain preferable when ethical data collection is feasible.

##### 6.8.1.2 Generative model dependencies

Occasional artifacts, mode collapse on rare presentations, and limited fine-grained attribute control; Section 5.3.3 quantifies how generator choice propagates to downstream MAE. Next-generation diffusion models will improve accordingly.

##### 6.8.1.3 Neonatal validation sample size

The neonatal validation set comprises 120 video segments from 15 neonates—a small sample that warrants careful interpretation. A post-hoc power analysis for binary classification (pain vs. no-pain) indicates that *N* = 120 samples provide approximately 72% power to detect a 10 percentage-point accuracy difference at α = 0.05 (two-sided binomial test), which falls below the conventional 80% threshold. Consequently, the reported 81.5% accuracy should be interpreted as a preliminary estimate, likely subject to wider confidence intervals in larger populations (Table 11 now reports these intervals explicitly). The prospective multi-site validation study described below targets *N* ≥ 60 neonates (approximately 480–600 video segments), which would provide >90% power for the same effect size at α = 0.05. The binary pain/no-pain task—rather than continuous N-PASS regression—was specifically selected for the neonatal evaluation precisely because it maximizes statistical power given the limited sample size (Koo and Li, 2016). These limitations are acknowledged and do not affect the validity of the UNBC-McMaster and BioVid evaluations, which involve 25 and 87 subjects, respectively, with established cross-validation protocols.

A prospective multi-site neonatal validation study is in the institutional planning phase. The target design is a multi-center observational study that will recruit *N* ≥ 60 neonates (approximately 480–600 video segments at 30-second granularity) across at least three NICUs, with power >90% to detect an ICC ≥ 0.80 at α = 0.05. Ethical framework preparation—including IRB protocol development and parental consent documentation—is underway in collaboration with three partner institutions.

##### 6.8.1.4 Absence of a fourth, fully external dataset

The present evaluation uses three independently collected benchmarks (UNBC-McMaster, BioVid, and an in-house neonatal cohort), which differ in institution, population, pain modality, and acquisition hardware. The cross-dataset transfer experiments in Section 5 (Table 22) provide evidence of generalization without target-domain adaptation. However, none of these three datasets was collected specifically as a held-out external validation set for this study, and all were used, at a minimum, as unlabeled real data during Stage 2/3 domain alignment for at least one experimental condition. Consequently, claims of generalizability should be interpreted as supported by internal cross-dataset transfer rather than by independent external validation. We have identified two candidate cohorts for future external validation: a multi-site European NICU pain-monitoring cohort and a geriatric/dementia pain cohort, neither of which was accessed, analyzed, or used in any capacity in the present study.

##### 6.8.1.5 Validation data requirements

Rigorous validation requires some labeled test data, even though training uses zero real labeled pain examples. Future: semi-supervised consistency checks and expert-in-the-loop active validation.

#### 6.8.2 Domain shift, clinical-setting robustness, and cultural variation

##### 6.8.2.1 Robustness across clinical settings

Real-world clinical deployment spans diverse hospitals, cameras, ethnicities, sensors, and acquisition protocols, unlike the three controlled benchmarks studied here (fixed camera position, controlled lighting, and single-institution hardware per dataset). NICU and ward environments introduce additional sources of domain shift not captured by either the synthetic generation pipeline or the unlabeled real data used for Stage 2/3 alignment: variable ambient lighting (including phototherapy blue-light exposure in NICUs), incubator glass reflections and occlusion, swaddling and medical equipment (CPAP masks, IV lines) occluding facial action units, and camera angles other than the fixed overhead position used during our data collection (Section 4.3). The robustness evaluation in Section 5.4 provides a partial, synthetic proxy for a subset of these shifts (illumination change, partial occlusion, and sensor degradation), but it is not a substitute for genuine multi-hospital, multi-camera, multi-ethnicity external validation, which remains future research (Section 6.8, “Absence of a Fourth, Fully External Dataset”). The domain alignment strategy (Section 3.4) was designed to bridge synthetic-to-real gaps under the acquisition conditions represented in D_*r*_, and its robustness to acquisition conditions *not* represented in D_*r*_ remains an open question pending multi-site deployment data.

##### 6.8.2.2 Cultural and display-rule variation

Pain expression is governed by well-documented cultural display rules that modulate the intensity and form of facial and behavioral pain signals, independent of the underlying nociceptive state (Hadjistavropoulos et al., 2014). Our synthetic generation pipeline conditions on ethnicity as a demographic attribute (Section 3.2) to promote balanced physical representation, but it does not explicitly model culturally conditioned differences in *expressive behavior*. The fairness analysis in Section 5.5 therefore measures parity in *prediction accuracy* across ethnicity labels within UNBC-McMaster, which is not equivalent to validating robustness to cross-cultural expression differences in unseen populations.

##### 6.8.2.3 Deployment challenges beyond hardware

Beyond the hardware and sensor-fallback considerations discussed in Section 6.5, real-world deployment introduces workflow and human-factors challenges not evaluated here: alert fatigue from continuous 1 Hz monitoring, integration into existing nursing workflows without displacing clinical judgment, liability and documentation requirements for AI-assisted pain scores, and the need for ongoing post-deployment performance monitoring to detect silent model drift as case mix or equipment changes over time.

#### 6.8.3 Clinical feasibility constraints

Real-world clinical pain assessment must also contend with patient states not captured by any of the three evaluation benchmarks, which would violate the assumptions built into the present pipeline. **Sedation and analgesic medication** blunt both facial AU activation and autonomic reactivity (Section 3.2.2); our synthetic generation does not condition on medication state, so a sedated patient in genuine pain could be systematically underestimated by both the visual and physiological branches simultaneously, a failure mode distinct from the isolated single-modality degradations tested in Section 5.4. **Facial paralysis or asymmetry** (e.g., post-stroke, Bell's palsy) would violate the implicit bilateral-AU assumptions of the facial encoder (Section 3.3) and the PSPI-based conditioning of the generator (Section 3.2). **Intubation and CPAP masks** mechanically occlude the lower face; the occlusion condition in Section 5.4 provides a generic proxy for this but was not specifically constructed to match medical-device occlusion geometry. **Neurological disorders affecting facial motor control** would require population-specific model variants, as already flagged in the atypical-presentation failure analysis of Section 5.7. None of sedation, facial paralysis, intubation/CPAP occlusion, or neurological facial-motor disorders were represented in the UNBC-McMaster, BioVid, or neonatal cohorts used in this study, and we recommend that clinical deployment explicitly exclude these subgroups pending dedicated validation.

#### 6.8.4 Technical limitations

##### 6.8.4.1 Missing modality handling

Architecture degrades when modalities are absent (e.g., no cry during sleep); Section 5.4 now quantifies this degradation directly rather than describing it only qualitatively.

##### 6.8.4.2 Computational resources

The 4 × A100 training requirement limits accessibility (Table 15); inference requirements are far lower (single consumer/edge GPU). Model distillation and efficiency optimizations could further improve training accessibility.

##### 6.8.4.3 Physiological signal availability

The vision-only variant achieves 74.6% on BioVid—still competitive but lacking physiological complementarity. Future: non-contact physiological extraction from video (remote PPG, respiration).

##### 6.8.4.4 Cultural and individual variations

Demographic diversity is included, but cultural display rules may not be fully captured.

##### 6.8.4.5 Uncertainty quantification scope

MC-Dropout (Section 5.9) is the lowest-overhead uncertainty method we implemented; Deep Ensembles and Bayesian regression were not implemented and may improve calibration further.

#### 6.8.5 Future directions

- **Few-shot adaptation:** Meta-learning for rapid adaptation from 5–10 examples per person
- **Deep ensembles and Bayesian regression:** Improved calibration beyond MC-Dropout (Section 5.9)
- **Formal SHAP/per-sample cross-modal attention heatmaps:** Beyond the aggregate attention-attribution signal of Section 5.8.4
- **Foundation-model visual backbones (DINOv2, SigLIP):** As a drop-in replacement ablation for the ViT-Base/16 encoder (Section 6.4)
- **Compounded and adversarial robustness testing:** Beyond the six isolated perturbations of Section 5.4
- **Active learning for validation:** Strategic sample selection to minimize labeling requirements
- **Federated learning:** Collaborative improvement without centralizing sensitive data
- **Multi-task learning:** Joint training for pain, distress, and treatment response prediction
- **Multi-site external validation:** Prospective study across 5+ diverse NICUs and the two candidate external cohorts named in Section 6.8

### 6.9 Broader implications for medical AI

This study presents a candidate methodological approach for medical AI development when real-world data collection faces ethical or practical barriers—potentially applicable to rare diseases, pediatric applications, emergency scenarios, psychiatric assessment, and end-of-life care. Each domain would require domain-specific synthetic generation informed by clinical knowledge and independent validation before any deployment claim can be made; the present results should be read as an initial feasibility demonstration rather than an established paradigm.

### 6.10 Ethical AI development as imperative

This study reflects an ethical commitment to developing medical AI without exploiting vulnerable populations. Our synthetic-source domain-adaptation approach substantially reduces the ethical burden, demonstrating that high-performance medical AI need not rely on ethically problematic data collection. The research community must prioritize: (1) minimizing data collection from vulnerable populations, (2) ensuring equitable representation, (3) transparently reporting limitations, (4) meaningful clinical validation, and (5) responsible deployment with human oversight.

## 7 Conclusion

This study demonstrated the feasibility of multimodal pain estimation trained with zero real labeled pain examples, using synthetic simulation combined with unsupervised domain adaptation—a setting we explicitly distinguish from classical zero-shot learning (Section 2.4). Our diffusion-based generation creates 50,000 scenarios (facial expressions, HRV, EDA, and temporal dynamics) with balanced demographic diversity, validated with an expanded battery of fidelity metrics (FID, SSIM, LPIPS, generative precision/recall, and AU-distribution testing; Section 5.1.3), while transformer fusion with three-stage alignment achieves: MAE 0.89 on UNBC (31% gap), 78.3% on BioVid (10.5% gap), and a preliminary 81.5% neonatal accuracy point estimate (+12.7 percentage points vs. clinical, with wide uncertainty given *N* = 120). Critically, 40.4% superior cross-dataset generalization on the three benchmarks tested is consistent with, though does not by itself prove, generalization to fully external clinical sites.

Ablation studies confirmed that each domain alignment component contributes substantially: contrastive learning (−14.0%), adversarial adaptation (−5.8%), and consistency regularization (−6.1%), with a synergistic combination essential, and a theoretical account of why in Section 3.4.5. New analyses added in this revision—Monte Carlo Dropout uncertainty quantification (ECE 0.041), robustness evaluation under six acquisition perturbations, generator architecture and dataset-scale ablations, and a computational complexity assessment—directly address gaps in rigor identified during peer review. Fairness analysis reveals no significant disparities across age, sex, or ethnicity (*p* > 0.05 in all groups). However, several subgroups remain statistically underpowered, and this should not be taken as evidence against smaller disparities.

Future research focuses on few-shot adaptation, Deep Ensemble/Bayesian uncertainty quantification, foundation-model visual backbones, compounded robustness testing, and multi-site prospective neonatal validation (*N* ≥ 60, ≥3 NICUs, currently in planning; see Section 6). By advancing automated pain monitoring with ethically sourced, synthetic-trained, uncertainty-aware predictions, this study contributes toward the goal of improved care for vulnerable populations who can least afford inadequate pain assessment.

## Data Availability

The original contributions presented in the study are included in the article/supplementary material, further inquiries can be directed to the corresponding author.
